# High Resolution Mapping of Bactericidal Monoclonal Antibody Binding Epitopes on *Staphylococcus aureus* Antigen MntC

**DOI:** 10.1371/journal.ppat.1005908

**Published:** 2016-09-30

**Authors:** Alexey V. Gribenko, Kevin Parris, Lidia Mosyak, Sheng Li, Luke Handke, Julio C. Hawkins, Elena Severina, Yury V. Matsuka, Annaliesa S. Anderson

**Affiliations:** 1 Pfizer Vaccine Research and Development, Pearl River, New York, United States of America; 2 Pfizer Protein Engineering and Production, Cambridge, Massachusetts, United States of America; 3 Department of Medicine, University of California at San Diego, La Jolla, California, United States of America; National Jewish Health, UNITED STATES

## Abstract

The *Staphylococcus aureus* manganese transporter protein MntC is under investigation as a component of a prophylactic *S*.*aureus* vaccine. Passive immunization with monoclonal antibodies mAB 305-78-7 and mAB 305-101-8 produced using MntC was shown to significantly reduce *S*. *aureus* burden in an infant rat model of infection. Earlier interference mapping suggested that a total of 23 monoclonal antibodies generated against MntC could be subdivided into three interference groups, representing three independent immunogenic regions. In the current work binding epitopes for selected representatives of each of these interference groups (mAB 305-72-5 – group 1, mAB 305-78-7 – group 2, and mAB 305-101-8 – group 3) were mapped using Hydrogen-Deuterium Exchange Mass Spectrometry (DXMS). All of the identified epitopes are discontinuous, with binding surface formed by structural elements that are separated within the primary sequence of the protein but adjacent in the context of the three-dimensional structure. The approach was validated by co-crystallizing the Fab fragment of one of the antibodies (mAB 305-78-7) with MntC and solving the three-dimensional structure of the complex. X-ray results themselves and localization of the mAB 305-78-7 epitope were further validated using antibody binding experiments with MntC variants containing substitutions of key amino acid residues. These results provided insight into the antigenic properties of MntC and how these properties may play a role in protecting the hostagainst *S*. *aureus* infection by preventing the capture and transport of Mn^2+^, a key element that the pathogen uses to evade host immunity.

## Author Summary

*Staphylococcus aureus* protein MntC is a metal-binding protein of the ABC-type transporter involved in the acquisition of an essential nutrient, Mn^2+^, by the pathogen. An earlier study demonstrated that use of MntC as an antigen in experimental vaccine can provide protection against staphylococcal infections in animals and identified three groups of protective monoclonal antibodies induced by the protein. In the current work we employed Deuterium-Hydrogen Exchange Mass Spectrometry (DXMS) to determine binding sites of selected representatives from each of those three groups. DXMS results were further validated using X-ray crystallography, site-directed mutagenesis and functional studies. Locations of the binding sites and results of the functional studies were used to draw conclusion on molecular mechanisms of protection afforded by MntC: antibodies belonging to two of the groups are predicted to interfere with Mn^2+^ transfer from the protein to the transmembrane channel pore, while the third group of the antibodies is expected to interfere with Mn^2+^ binding to MntC itself. The net result in both cases is impaired Mn^2+^ transport across the bacterial membrane and increased susceptibility of the bacterium to the oxidative stress, likely due to the reduced activity of superoxide dismutase which requires Mn^2+^ as an essential co-factor for activity.

## Introduction

**Fig 1:**
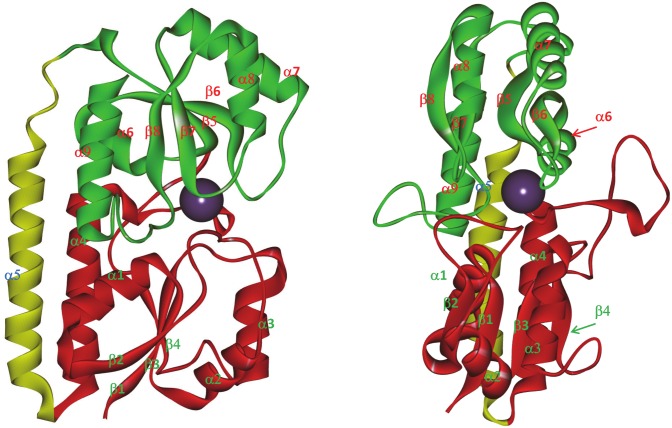
Three-dimensional structure of *S*. *aureus* MntC. The figure is rendered using Accelrys Discovery Studio Visualizer 2.5 (PDB ID 4K3V). The N-terminal domain of the protein is shown in red, the C-terminal domain is shown in green, and the connecting “backbone” α-helix is shown in yellow. The purple ball represents the Mn^2+^ ion in the binding site. Individual structural elements are labeled to illustrate the numbering convention used throughout the article.

*Staphylococcus aureus* protein MntC is the ligand-binding component of the ABC-type manganese transporter MntABC, which is at least partially responsible for the organism’s resistance to the oxidative stress [1,2]. The protein is expressed during early stages of infection [3] and binds manganese with high affinity [4]. Active vaccination with recombinant *S*. *aureus* MntC reduced bacterial burden in a murine bacteremia model of infection [3], making MntC a promising vaccine candidate. As such, MntC is a key component of an experimental four-antigen (SA4Ag) *S*. *aureus* vaccine which is currently undergoing clinical trials (US government registry, trials #NCT01364571, #NCT01643941 and #NCT02364596 –completed, trials #NCT02388165 and #NCT02492958 –ongoing). We have recently solved the 3-dimensional structure of MntC by X-ray crystallography (PDB ID 4K3V, Fig 1) and provided detailed biophysical characterization of the protein [4]. Furthermore, in an earlier work from our laboratories [3] we reported generation of 23 monoclonal antibodies against MntC, with some of them being protective in an infant rat model of infection and being capable of inducing respiratory burst activity of neutrophils. Based on BIAcore binding interference patterns, these antibodies were subdivided into three interference groups [3]. In the current report we identified binding epitopes for selected representatives of each of those groups.

Monoclonal antibodies are powerful tools for biomedical research and biotherapeutics. Their ability to recognize and bind specific targets with very high affinity is invaluable for a wide variety of *in vitro* and *in vivo* applications [5]. In vaccine research and development monoclonal antibodies are used, (i) to identify epitopes that elicit functional immune responses, (ii) to study surface expression of antigens on clinical isolates *in vivo* and *in vitro*, (iii) to assess preservation of the functionally important epitope(s), (iv) in antigen identity testing, and (v) in product quality control. Understanding the mechanisms of the antibody-antigen interaction via epitope mapping is of utmost importance in biopharmaceutical research and development, and in biomedical research in general. The knowledge gained from monoclonal antibody epitope mapping can augment targeted vaccine development [6,7], and aid in the development of new therapies. Furthermore, these studies can increase the understanding of the molecular mechanisms of existing therapies and shed light on the pathogenic mechanisms utilized by these disease-causing agents.

One of the common strategies for monoclonal antibody epitope identification is synthesis of overlapping linear peptides derived from the antigen amino acid sequence, with subsequent identification of the peptides that bind to the antibody of interest [8]. A somewhat similar strategy involves attachment of the peptides to a solid support, creating peptide arrays [9], followed by probing of the arrays with the antibody of interest. While straightforward, these approaches are limited to the identification of linear epitopes. As such, they are not particularly useful for identification of protective epitopes, the vast majority of which are conformational [10,11]. The same challenge hampers epitope mapping using phage display (recently reviewed by Pande and co-workers [12]). Limited proteolysis followed by mass spectrometry [13–15] has the potential to identify conformational epitopes, however the resolution of this technique is quite limited. The only two approaches currently capable of providing residue-specific description of the antibody-binding epitopes are X-ray crystallography and NMR spectroscopy. Technical challenges associated with these methods include potential difficulties in co-crystallization of the antigen-antibody complexes for X-ray crystallographic analysis and antigen molecular mass restrictions for NMR spectroscopy. The need to produce Fab fragments of the antibodies, requirements for extremely pure material and low throughput further limit efficiency of the two techniques.

Hydrogen-deuterium exchange mass spectrometry (DXMS, also abbreviated as HDX-MS) is emerging as a method of choice for monoclonal antibody epitope mapping. Distinct advantages of DXMS over many of the methods described above have been thoroughly reviewed elsewhere [16], and will not be discussed here in detail. Briefly, DXMS is not limited to linear epitopes, provides higher resolution than proteolytic methods, does not require knowledge of the three-dimensional structure of the antigen, and provides higher throughput than X-ray crystallography without the same sample requirements (i.e., amount of material and high sample purity). The resolution of the method, however, may be lower than X-ray crystallography, although single amino acid resolution can be achieved in principle through method optimization, proper selection of proteolytic enzymes, digestion conditions or simultaneous fitting of the exchange data obtained from multiple overlapping peptides [17]. Basic principles of the technique have been described in several recently published review articles [18–20], and will not be repeated here. DXMS was used in numerous studies to identify monoclonal antibody binding epitopes on diverse targets, ranging from food allergens to vaccine antigens [16,21–30], as well as in several protein folding studies aimed at understanding of the folding intermediate structures [31,32] or identification of the core regions during amyloid formation [33]. Direct comparisons of DXMS results to the epitope mapping “gold standard” method of X-ray crystallography are still fairly scarce, however.

In the current work we employed DXMS to identify binding epitopes of selected bactericidal monoclonal antibodies raised against MntC [3]. DXMS results were validated by co-crystallizing MntC with the Fab fragment of one of these antibodies, mAB 305-78-7. The results were further confirmed in mAB 305-78-7 binding studies using MntC variants with site-directed amino acid substitutions at selected key residues involved in the epitope formation. Experimental data reported here provide useful insights into the mechanisms of protection afforded by the anti-MntC antibodies. It should be noted that an earlier work by Ahuja and co-workers [34] identified one of the protective epitopes on MntC. The results reported here identifed two more immunogenic regions on the protein and showed that the third antibody under study shares epitope with that of FabC1 identified by Ahuja et al.

## Materials and Methods

### DXMS

General operational procedures and DXMS apparatus have been previously described in detail [35–39]. Optimization of proteolytic conditions and digest mapping of MntC have been reported elsewhere [4]. MntC and monoclonal antibodies were extensively dialyzed against PBS, pH 7.4 and concentrated to ~10 mg/mL using Amicon concentrators with molecular weight cutoff of 10 kDa. D_2_O-based PBS was prepared by diluting 10x PBS stock (Cellgro, cat.# 46-013-CM) with 100% D_2_O, thereby bringing deuterium oxide concentration to 90%. MntC was combined with the antibodies in a 1.5:1 molar ratio (MntC:antibody, i.e., 1.5 moles of MntC per 2 moles of binding sites) to ensure that no unbound protein would remain in solution and incubated on ice for 60 minutes. Hydrogen-deuterium exchange was initiated by adding 4.3 volumes of cold D_2_O-based PBS to 1 volume of the antigen-antibody mixture (final D_2_O concentration was, therefore, 73%). After 10, 100, 1,000, 10,000 and 100,000 second incubation on ice, 64 μL samples were mixed with 96 μL of ice-cold quenching buffer (0.8% formic acid, 16.6% glycerol, 0.08 M Gdm-HCl) to stop the exchange. Aliquots of 30 μL were frozen on dry ice and stored at -80°C until further use. Control samples (without antibodies) were prepared in the same way, except that they were initially diluted with H_2_O-based PBS to obtain desired protein concentration (15 μg total protein per 30 μL of the final frozen aliquot) before initiating the exchange. To prepare non-deuterated MntC, 40 μL of 10 mg/mL protein stock was combined with 387 μL of H_2_O-based PBS and “quenched” as described above for the deuterated samples. Fully deuterated samples were prepared by incubating MntC (1.25 mg/mL final concentration) in 0.5% formic acid, 73% D_2_O for 48 hours at room temperature and quenched, as described above. Proteolytic digestion, HPLC separation, mass spectrometric analysis and peptide identification were done exactly as previously described [4,40]. The amount of deuterium accumulating on each peptide at different time points with or without the antibody present was determined from changes in molecular weight of the corresponding peptide (calculated from the peak centroids) as a function of time

$$\#D=D_{\text{max}}\cdot \frac{Mw(T)-Mw(ND)}{Mw(FD)-Mw(ND)}$$ (1)

where #D is the number of deuterons exchanged at each time point, D_max_ is the maximum number of hydrogens that can be exchanged for deuterium on the peptide (equal to the number of residues minus number of prolines minus 2 [41]), Mw(T) is the centroid peak value of the peptide at time T, Mw(ND) is the centroid peak value of the non-deuterated peptide, Mw(FD) is the centroid peak value of the fully deuterated peptide. Antibody binding epitopes were identified from the peptides showing decreased deuterium accumulation in the presence of the antibody. Back-exchange was not taken into account, since all samples were prepared identically and absolute deuterium accumulation numbers are not necessary to identify the epitopes.

### Complex co-crystallization and three-dimensional structure analysis

Cloning, expression and purification of recombinant MntC, as well as generation of the monoclonal antibody 305-78-7, have been previously described [3]. The mAB 305-78-7 Fab fragment was generated using Thermo Scientific’s Pierce mouse IgG_1_ Fab and F(ab’)_2_ preparation kit (catalog number 44980), according to the kit manufacturer’s instructions. The macromolecular complex between MntC and the Fab fragment of mAB 305-78-7 was generated by combining equimolar amounts of MntC (40 mM Tris pH 7.5, 0.3 M NaCl) and the Fab fragment (TBS pH 8.0). The resulting solution was passed through a size exclusion column (Superdex 200 (10/300)) equilibrated in 10 mM HEPES pH 7.5 and 150 mM NaCl. The sample was concentrated to 5.16 mg/mL and crystals of the complex containing MntC and the Fab fragment of mAB 305-78-7 were grown by the hanging drop method using 1 M LiCl, 0.1 M MES, pH 6.0 and 20% PEG 6K as the precipitating reagents. The crystals were cryopreserved by adding 20% glycerol to the precipitating buffer and snap freezing in liquid nitrogen. The X-ray diffraction data were collected to 1.8 Å resolution at the Argonne National Laboratory (APS) using the 22-ID beamline (SER-CAT, Southeast regional collaborative access team) and then reduced and scaled with HKL2000 [42]. Initial phases were estimated by molecular replacement using the Fab fragment from PDB entry 1QGC as the input model in PHASER [43]. Molecular replacement yielded a single solution and the model was subjected to several rounds of rebuilding in COOT [44,45] alternating with subsequent minimization using BUSTER [46]. The quality of the model was judged by the decrease in R-factors. Refinement converged after several rebuilding cycles to an R-factor of 20.7% and Rfree of 23.6%. Crystallographic data collection and refinement statistics are summarized in S1 Table. The final model contains protein residues 16–113 and 118–292 for MntC, residues 1–212 for the Fab light chain, residues 1–126 and 134–213 for the Fab heavy chain, one glycerol molecule, one metal ion, and 197 water molecules. MntC residues 114–117 and Fab heavy chain residues 127–133 were not detected in the electron density maps due to disordering.

### Cloning, expression and purification of recombinant mutant MntC proteins

All oligonucleotides were synthesized, and, when necessary, HPLC-purified by IDT (Coralville, IA). Q5 High-Fidelity DNA Polymerase for PCR and all restriction endonucleases were purchased from New England Biolabs (Ipswich, MA) and were used according to manufacturer’s recommendations.

pLH94, a vector for expression of the MntC H234F E247L K254M variant protein with no 6xHis-tag (used in the ITC experiments), was generated by two rounds of site-directed mutagenesis. To introduce MntC H234F and K254M substitutions into pLP1215, a vector for expression of wild-type MntC, mutagenesis was performed with the QuikChange Lightning Multi Kit (Agilent Technologies, Santa Clara, CA) according to the manufacturer’s instructions. The QuikChange Primer Design Program (Agilent Technologies) was used to generate sequences of mutagenic oligonucleotides oLH553, oLH556, and oLH557 (S2 Table), which were used in the first round of mutagenesis. The mutagenesis reaction was transformed into *E*. *coli* XL-10 Gold cells (Agilent Technologies). The presence of mutations yielding the MntC H234F K254M allele and the absence of secondary mutations was confirmed by DNA sequencing. This resulting plasmid, pLH91, was subjected to an additional round of mutagenesis with the QuikChange II XL kit (Agilent Technologies). The kit was used according to the manufacturer’s instructions, and mutagenic oligonucleotides used in the reaction (oLH569 and oLH570, S2 Table), were designed with the QuikChange Primer Design Program. The mutagenesis reaction was transformed into *E*. *coli* XL-10 Gold cells. The presence of mutations yielding the MntC H234F E247L K254M allele and the absence of secondary mutations were confirmed by DNA sequencing. Recombinant protein was expressed and purified as described earlier [3].

Two additional primers (S2 Table) were used in generating vectors encoding histidine-tagged proteins subsequently used in Octet analysis. Wild-type, single mutant, and combination mutant MntC alleles were amplified by PCR with primers pLP1215 NdeI_S and pLP1215 BamHI_AS (S2 Table), and the resulting fragments were cloned into pCR-Blunt II-TOPO vector (Life Technologies, Carlsbad, CA). These inserts were excised with an *Nde*I/*Bam*HI digest, gel-purified, and cloned into pET-19b at the *Nde*I/*Bam*HI sites in-frame with the N-terminal His-tag encoded by the vector. The presence of the desired mutations and the absence of secondary mutations were confirmed by DNA sequencing. The final expression constructs were transformed into *E*. *coli* BL21(DE3) chemically competent cells (Thermo Fisher Scientific, Waltham, MA). The expression host strains were grown in Terrific Broth (500 mL, 37°C, 225 rpm shaking) until the cultures reached exponential phase. IPTG was added to a final concentration of 1 mM, and the cells were harvested 4 hours later. Cells were lysed with a French pressure cell press, and recombinant MntC proteins were purified with HisPur Ni-NTA Agarose (Thermo Fisher Scientific) in batch mode followed by size-exclusion chromatography.

### Circular dichroism spectroscopy

The folding state of MntC-pLH94 was confirmed using far- and near-UV CD spectroscopy. Far- and near-UV CD spectra were collected on a Jasco J-810 spectropolarimeter in rectangular quartz cuvettes with path lengths of 1 mm and 1 cm, respectively. Temperature was controlled using Jasco Peltier-type PTC-423S cell holder. Far-UV CD spectra were recorded in PBS, pH 7.4 at the protein concentration of 0.12 mg/mL. Spectral interval– 200–260 nm, step size– 0.1 nm, bandwidth– 3 nm, experimental temperature– 20°C, scanning speed– 50 nm/min, 5 accumulations were averaged for each spectrum. Near-UV CD spectra were recorded in 50 mM Na cacodylate pH 7.0, 150 mM NaCl. Spectral interval of the near-UV CD data– 250–320 nm, other scan parameters were the same as in the far-UV CD experiments. Data were corrected by subtracting spectra of the corresponding buffers and smoothed using adjacent neighbor averaging of 21 points.

### Isothermal Titration Calorimetry (ITC)

All of the isothermal titration calorimetry experiments were done on a VP-ITC instrument (Microcal, Northampton, MA). Mn^2+^ binding to the wild type MntC versus MntC-pLH-94 was characterized in 50 mM Na cacodylate pH 7.0, 150 mM NaCl. 32.5 μM solution of wild type MntC or 31.8 μM solution of MntC-pLH94 were titrated with a 0.33 mM solution of MnCl_2_ at 37°C. An initial 2-μL injection was followed by 8-μL injections at 240 second intervals until no heat exchanges were observed. Injection rate was 0.5 μL/sec. Reference power was set to 15 μcal/sec. To characterize MntC interactions with mAB 305-78-7, 3.3 μM solutions of the antibody in 50 mM Na cacodylate pH 7.0, 150 mM NaCl were titrated with 112–143 μM solutions of either wild type MntC or MntC-pLH94 in the same buffer. Experiments were run at 37°C. An initial 2 μL injection was followed by 6 μL injections at 240 second intervals until no heat exchanges were observed. Mn^2+^ binding to MntC bound to the monoclonal antibodies was characterized in 50 mM Tris-HCl, 150 mM NaCl, pH 7.4. 11–24 μM solutions of MntC were initially saturated with the Fab fragment of mAB 305-78-7 or intact antibodies 305-72-5 and 305-101-8 (saturation was monitored by ITC) and then titrated with 0.254 mM MnCl_2_ to saturation. Mn^2+^ and antibody binding data were fit to the “single class of binding sites” model using data analysis software provided by the ITC manufacturer to extract thermodynamic binding parameters.

### Titrations of monoclonal antibodies 305-78-7 and 305-101-8 with synthetic peptides derived from the epitope sequences

Peptides with sequences SVDKKAMESLSEETKKDIFGEVY (corresponding to MntC residues 240–261 with a tyrosine residue added at the C-terminus for UV absorbance measurements) and EINTEKQGTPEQMY (corresponding to MntC residues 218–220 with a tyrosine residue added at the C-terminus for UV absorbance measurements) were chemically synthesized via Thermo Scientific Custom Peptide Synthesis Service. Monoclonal antibodies 305-78-7 and 305-101-8 were dialyzed against 1x PBS, pH 7.4 and titrated with the corresponding peptides dissolved in the same buffer or full length MntC protein dialyzed against the same buffer at 25°C. When appropriate, data were fit to the binding model describing interaction with a single class of binding sites, as described above.

### Enzyme-Linked Immunosorbent Assay (ELISA)

Microtiter plate wells were coated overnight with 2 μg/mL of wild type MntC, MntC-pLH94 or BSA control (100 μL/well). Wells were subsequently blocked with 200 μL /well of 2% Nonfat Dry Milk in PBS, pH 7.4, 0.05% Tween-20 (blocking solution) for 60 min at room temperature. Serial dilutions of mAB 305-78-7 (100 μL each) were added to the wells and incubated at 37°C for 2 hours. Plates were then washed 3 times with the blocking solution (200 μL /well) and incubated for 60 minutes at 37°C with alkaline phosphatase-conjugated goat anti-mouse IgG (1:3000 dilution, 100 μL /well). Immunocomplexes were detected via absorbance measurements at 405 nm using 1-Step pNPP (“Pierce”, #37621) chromogenic substrate (100 μL /well). Dissociation constants were determined from fitting of the absorbance data to the equation

$$A=\frac{A_{\text{max}}[L]}{K_{d}+[L]}$$ (2)

where *A* is absorbance at ligand concentration *[L]*, *A*_*max*_ is absorbance at saturation and *K*_*d*_ is the dissociation constant.

### Bio-Layer Interferometry (Octet) assessment of mAB 305-78-7 binding to recombinant mutant MntC proteins

Binding affinity of mAB 305-78-7 to recombinant mutant MntC proteins was measured with a ForteBio Octet HTX instrument (Pall Life Sciences, Menlo Park, CA). Experiments were conducted in 96-well black opaque plates (Greiner Bio-One, Monroe, NC) with 30°C incubation temperature and 1000 rpm agitation. To ensure proper immersion of the biosensors, each well contained a final volume of 200 μL. Anti-murine IgG Fv biosensors (AMQ, Pall Life Sciences) were saturated with mAB 305-78-7 and then incubated with two-fold serial dilutions of recombinant MntC proteins. Duplicate measurements were captured within each experiment by reusing the reagent samples with fresh biosensor tips. Kinetic measurements were repeated two times in independent experiments to determine average values. Data acquisition and analysis was performed with Octet software version 8.2 (Pall Life Sciences). Buffer-subtracted kinetic data were globally fitted with a 2:1 Langmuir model to obtain a dissociation rate constant (*K*_*D*_ = *k*_off_/*k*_on_).

### Accession number

Structural coordinates of MntC-Fab 305-78-7 complex have been submitted to the Protein Data Bank with the accession code 5HDQ.

## Results

### mAB epitope mapping using DXMS

**Fig 2:**
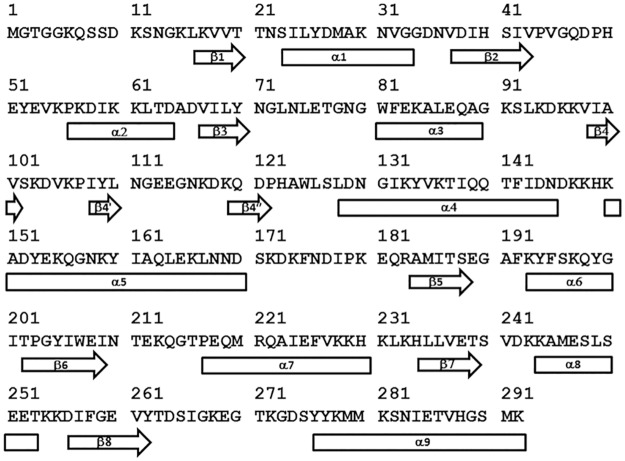
Amino acid sequence of MntC used in this study. Shown is the sequence of MntC with amino acid numbering used to identify specific structural elements referred to in this study. Secondary structural elements (rectangles – α-helices, block arrows – β-sheets) are mapped onto the sequence, based on the published three-dimensional structure of MntC (4K3V) [4]. Short β-sheets formed by residues 108–110 and 119–122 are defined in only one of the two MntC molecules in the crystallographic unit, hence they are designated β4’ and β4”, respectively, in order to maintain consistent labeling between the two molecules of the unit.

**Fig 3:**
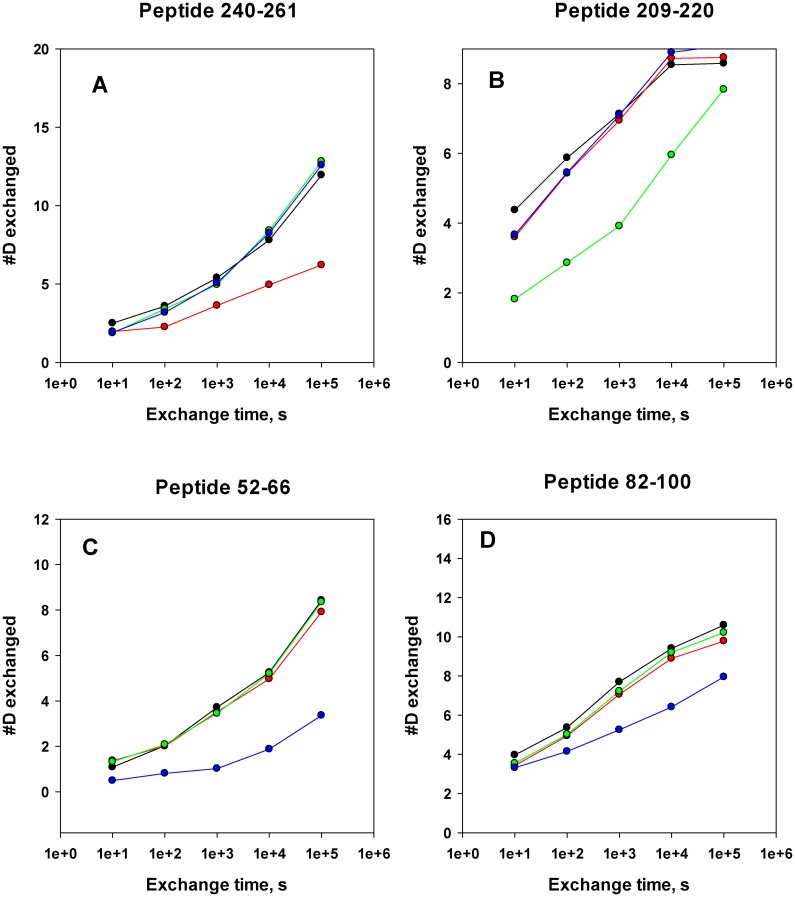
Typical deuterium accumulation plots used to identify monoclonal antibody binding epitopes on MntC. Black symbols and lines—MntC alone, red symbols and lines—deuterium accumulation on MntC in the presence of mAB 305-78-7, green symbols and lines—deuterium accumulation on MntC in the presence of mAB 305-101-8, blue symbols and lines—deuterium accumulation on MntC in the presence of mAB 305-72-5. Peptide 240–261 (panel A) includes residues recognized by mAB 305-78-7, peptide 209–220 (panel B) includes major part of the mAB 305-101-8 epitope, peptides 52–66 and 82–100 (panels C and D) include residues recognized by mAB 305-72-5. The plots are scaled to the maximum number of deuterons that can exchange onto a given peptide. Deuterium accumulation plots for the corresponding peptides from all of the experiments are shown on every panel for comparison.

Proteolytic digest mapping results (a necessary prerequisite of DXMS experiments) have been published [4] and will not be described here. Hydrogen exchange data are presented as deuterium accumulation plots. Number of deuterons exchanged onto the amide backbone within a given peptide is calculated as described in “Materials and Methods” and plotted as a function of incubation time in D_2_O. Each individual peptide is identified by the position of the first and last residue of that peptide within the amino acid sequence of the intact protein. Protein sequence aligned with the corresponding secondary structural elements is shown in Fig 2. Examples of typical results of the on-exchange DXMS experiments used to map monoclonal antibody binding epitopes are presented as deuterium accumulation plots in Fig 3 and S1–S3 Figs.

Antibody binding epitopes are identified through comparison of the deuterium accumulation plots for each peptide obtained with and without the antibody present. Conclusions on decreased deuterium accumulation in the presence of the antibodies (or lack thereof) were drawn based on the combination of multiple criteria: (1) significant difference in deuterium accumulation (>10% of the maximum number of deuterons that can be exchanged onto a given peptide) between the protein alone and the protein-antibody complex, (2) qualitative agreement between data from multiple time points for the same peptide, (3) quantitative agreement between data obtained for multiple charge states of the same peptide (when available), and (4) consistency of the data obtained from multiple overlapping peptides covering the same part of the protein sequence. It should be noted that each peptide “reports” on the exchange that is happening starting with amino acid #3 of that peptide, since the exchange on the two N-terminal amino acids is instantaneous and is not informative [41]. For example, peptide 259–276 reports on residues 261–276, since no information can be obtained for residues 259–260.

In some cases, it appears that a peptide may be exchanging more protons than physically possible. One has to keep in mind, that the fully deuterated sample used to determine Mw(FD) from Eq 1 was prepared at acidic pH, while individual time point samples were allowed to exchange at neutral pH. It is possible that conformation of the unfolded polypeptide chain at neutral pH is somewhat different than at acidic pH, with greater solvent accessibility of the backbone and, correspondingly, higher deuterium accumulation on the most flexible structural segments at neutral pH. This would produce apparent exchange levels greater than 100%. The issue can be disregarded, however, since the epitopes are identified from differences in deuterium exchange on the same structural segment with and without antibody present, rather than from the absolute levels of deuterium accumulation.

**Fig 4:**
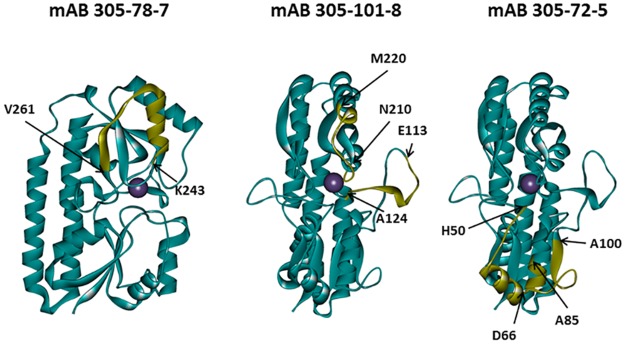
Mapping of the monoclonal antibody binding epitopes derived from DXMS experiments onto the three-dimensional structure of MntC. Epitopes are highlighted in yellow with positions of the first and last residues of each segment indicated. MntC molecule orientations are the same, as shown in Fig 1.

Functional antibody mAB 305-78-7 belongs to the interference group 2 (of the three interference groups identified in an earlier work [3]). A total of 128 common peptides could be identified between the digest maps of MntC alone versus MntC bound to the antibody (presence of more than one charge state for some of the peptides brings the total number of deuterium accumulation plots used in the analysis to 177). These peptides cover 100% of MntC sequence. Comparisons of the deuterium accumulation plots obtained in these experiments are shown in Fig 3 and S1 Fig. There is no difference in deuterium accumulation evident in any of the peptides up to (and including) the peptide 226–242. Starting with peptide 226–247, a small but persistent difference is observed in all peptides extending beyond amino acid D242. This difference becomes noticeably greater as the C-termini of the peptides extend beyond amino acid L249 (i.e., starting with the peptide 239–258, which is the first peptide to include amino acid S250), and then essentially disappears once N-termini reach amino acid V261 and beyond (peptides 259–275 and 259–276 are the first to include V261). Based on these data, we can conclude that the functional monoclonal antibody mAB 305-78-7 binding epitope is located between K243 and E260. Mapping these residues onto the three-dimensional structure of MntC (Fig 4) shows that the epitope involves α-helix α8, β-sheet β8 and a short loop connecting these two structural elements.

Another functional monoclonal antibody that we mapped using DXMS, mAB 305-101-8, belongs to the interference group 3 [3]. We identified a total of 132 peptides that are common between MntC and MntC bound to 305-101-8 (178 deuterium accumulation plots, which include more than one charge state for some of the peptides). Similar to the mAB 305-78-7 experiment, these peptides cover 100% of the protein sequence. Comparisons of the deuterium accumulation plots are shown in Fig 3 and S2 Fig. A small but consistent decrease in deuterium accumulation in the presence of the antibody is observed between amino acids 113–124 (peptides that include these amino acids are 111–124 through 111–126). A far greater decrease can be detected between residues N210 and M220 (peptides 208–220 through 217–220). Mapping these two fragments onto the MntC crystal structure (Fig 4) shows that they can be in close proximity, given high flexibility of the long loop encoded by amino acids 113–124, which connects β-sheet β4 and α-helix α4. Residues within this loop are not even observed in one of the two MntC copies in the asymmetric unit of the X-ray structure 4K3V [4] or in the structure of MntC-Fab 305-78-7 complex described below. Residues 210–220 encode another loop that connects β6 and α7, along with the first turn of the helix α7. Based on our DXMS data and X-ray structure, we can conclude that mAB 305-101-8 binding epitope maps between residues N210-M220, with an additional point of contact possibly provided by the tip of the loop connecting β4 and α4 (Fig 4). It should be noted that the epitope appears to overlap with the recently identified epitope of the monoclonal antibody fragment FabC1, which was shown to prevent MntC interaction with the MntB membrane importer[34].

The third functional monoclonal antibody that we mapped, mAB 305-72-5, belongs to the interference group 1, as defined by Anderson and co-workers[3]. A total of 63 common peptides (84 deuterium accumulation plots) were identified after proteolytic digestion of MntC alone and MntC in complex with the antibody. These peptides cover 93% of the sequence: no peptides representing the N-terminal 16 amino acids and a short fragment between amino acids 221–225 could be identified in the experiment with MntC bound to the antibody. However, the antibody binding epitope appears to be outside of either of these two regions. Comparisons of the deuterium accumulation plots used to map the mAB 305-72-5 epitope are shown in Fig 3 and S3 Fig. The first difference in deuterium accumulation in MntC vs. MntC bound to the antibody can be observed at peptides 28–51 and 37–51. This apparent 1–2 deuteron difference is the same between 28–51 and 37–51, suggesting that the epitope lies beyond amino acid 39 (which is the first amino acid of the 37–51 peptide, where exchange can be detected by DXMS). The next series of peptides (52–66 and 53–66) show apparent difference of ~ 5 deuterons. Coupled with the peptide 37–51 results, a possible explanation is that the epitope covers the last few residues of peptide 37–51 and the first few residues of peptide 52–66. The epitope structure, however, appears to be more complex: residues 69–81 show no difference in deuterium accumulation (peptides 67–74 and 75–81), however an apparent ~2 deuteron difference re-appears within the peptides extending beyond W81 to amino acid residue A100. The difference is the same up to at least peptide 83–100, suggesting that additional points of contact lie between amino acids 85–100. Taking all of these results together, we can conclude that the mAB 305-72-5 binding epitope is conformational and that this deuterium exchange pattern can only be explained by the antibody binding at the continuous surface formed by residues that are located somewhere between H50-D66 and A85-A100. Mapping these residues onto the X-ray structure of MntC (Fig 4) illustrates that this surface is likely formed by α-helix 2 and the last turn of α-helix 3.

### Three-dimensional structure of the MntC-Fab 305-78-7 complex solved by X-ray crystallography

**Fig 5:**
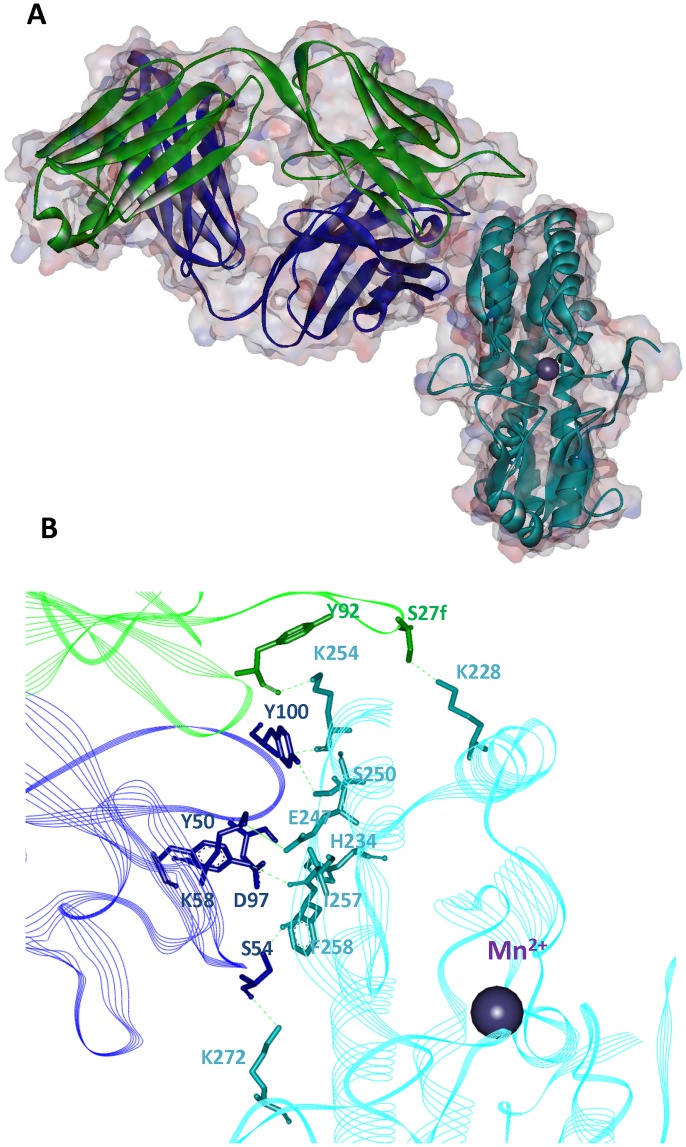
Three-dimensional structure of the MntC-Fab 305-78-7 complex. Overview of the complex structure (panel A). MntC backbone is shown in light blue color, backbone of the heavy chain of the Fab fragment is shown in dark blue, backbone of the light chain of the Fab fragment is shown in green. Expanded view of the interaction interface (panel B). MntC residues and backbone—light blue, heavy chain of the Fab fragment—dark blue, light chain of the Fab fragment—green. Residues involved in salt bridge formation and hydrogen bonding are shown in the stick representation. Hydrogen bonds are shown as dashed lines, and the salt bridge between E247 of MntC and K58 of the heavy chain is shown as a solid line.

Crystallographic analysis of the MntC-Fab 305-78-7 complex was used to validate epitope mapping results obtained via DXMS. The complex was co-crystallized and the structure was solved to 1.8 Å resolution (Fig 5A). MntC amino acid residues located within 4 Å of the heavy and light chains of the Fab fragment that are part of the binding interface (but not necessarily forming hydrogen bonding or salt bridge interactions) are listed in Table 1. According to the X-ray data, mAB 305-78-7 binds to the C-terminal lobe of MntC. The interface is primarily formed by the α-helix α8 and β-sheet β8. Fab residues potentially forming hydrogen bonds with MntC are highlighted in Table 1 and shown in Fig 5B. In addition, E247 of MntC may be forming a salt bridge with K58 on the heavy chain of the antibody.

**Table 1:** MntC and mAB 305-78-7 amino acid residues involved in the formation of the binding interface. All MntC residues that are listed in the table (left column) are located within 4 Å of either heavy or light chain residues of the antibody (shown in the right column). The residues were identified using Accelrys DS Visualizer 4.5. MntC residues forming hydrogen bonds with the antibody are H234, K254, D256, K272. MntC residue E247 forms a salt bridge with the antibody residue K58 on the heavy chain.

MntC Residues	Fab residues
K228	Light chain, S27f
H234	Heavy chain, D27
V237	Heavy chain, Y57
K243	Heavy chain, Y57
E247	Heavy chain, Y57
	Heavy chain, K58
S250	Heavy chain, Y100
E251	Heavy chain, Y100
E252	Light chain, Y27d
T253	Light chain, Y27d
K254	Heavy chain, G99
	Heavy chain, Y100
	Light chain, Y27d
	Light chain, Y32
	Light chain, Y92
	Light chain, S93
K255	Heavy chain, Y100
D256	Heavy chain, Y50
	Heavy chain, G99
	Heavy chain, Y100
	Light chain, Y94
I257	Heavy chain, Y50
	Heavy chain, Y56
F258	Heavy chain, N52
	Heavy chain, S53
	Heavy chain, S54
	Heavy chain, Y56
	Heavy chain, D97
G259	Heavy chain, S54
	Heavy chain, Y56
E260	Heavy chain, Y56
K272	Heavy chain, S54

### Disruption of mAB 305-78-7 binding epitope on MntC via site-directed mutagenesis

Amino acids involved in MntC-antibody contacts were identified from the crystal structure of the complex described above. A total of four MntC side chains form hydrogen bonding interactions with the antibody (H234, K254, D256 and K272), while E247 potentially forms a salt bridge with K58 on the heavy chain (Table 1). In an attempt to validate our epitope mapping results, while at the same time minimizing potential structural disruptions, we targeted only two residues involved in hydrogen bonding (H234 and K254) and salt bridge-forming E247 for replacement. Alternative amino acids were chosen to minimize effects of substitutions on side chain geometry, while completely eliminating chemical functionality of the side chains. Mutated MntC variant MntC-pLH94 was designed to contain three amino acid substitutions: H234F, E247L and K254M.

Folding state of MntC-pLH94 was confirmed via far- and near-UV CD spectroscopy. CD spectra of MntC-pLH94 are shown in S4 Fig, with CD spectra of the wild type MntC shown for comparison. According to these data, amino acid substitutions H234F, E247L and K254M had no detectable effect on secondary and tertiary structure of the protein: CD spectra of the wild type protein and MntC-pLH94 are essentially identical. To provide additional evidence for the lack of global structural change induced by the amino acid substitutions H234F, E247L and K254M, we conducted ITC experiments with MntC-pLH94 to monitor Mn^2+^ binding to the protein. A decrease or lack of Mn^2+^ binding as a result of these amino acid substitutions distal from the Mn^2+^ binding site would indicate that global protein structure has been significantly disrupted by the mutations. The results of the ITC experiments are shown in S5 Fig and binding parameters are listed in the S3 Table. Thermodynamic parameters of Mn^2+^ binding by MntC-pLH94 are essentially identical to those of the wild type protein, providing additional evidence that the H234F, E247L and K254M substitutions had no effect on the global structure of MntC.

**Fig 6:**
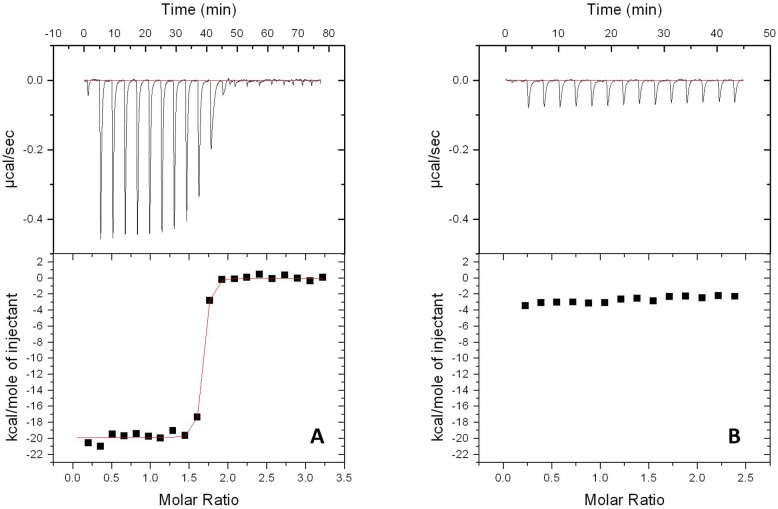
Binding of wild type MntC and MntC-pLH94 to mAB 305-78-7 monitored by ITC. Binding studies conducted with wild type MntC (panel A) and MntC-pLH94 (panel B) are illustrated. Upper panels show experimental heat flow and lower panels show the integrated heat of each individual injection (symbols) with the fit of the experimental data obtained with wild type MntC shown as red line on the left side of the figure. No binding was observed with MntC-pLH94, so no fitting is possible.

**Fig 7:**
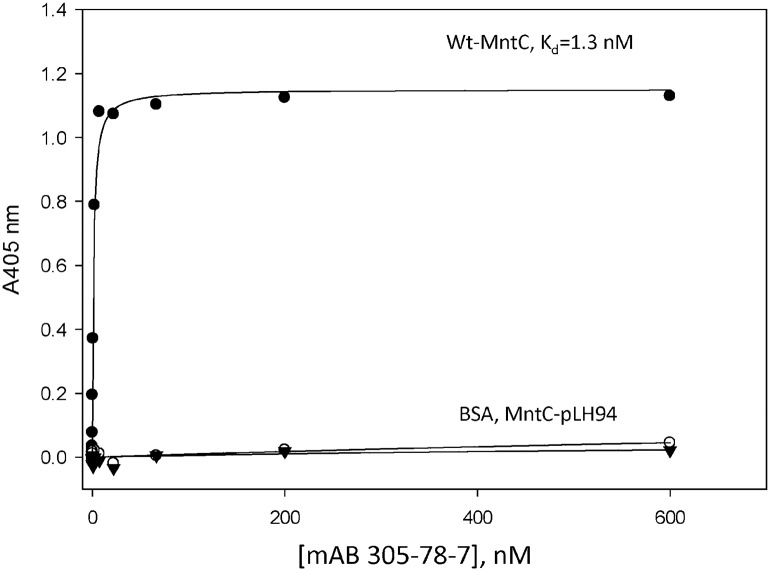
Binding of wild type MntC and MntC-pLH94 to mAB 305-78-7 analyzed using ELISA. Binding studies conducted with wild type MntC (black circles), MntC-pLH94 (black triangles), or BSA as a nonspecific control (open circles) are illustrated. The solid line represents the fit of the experimental data to the binding Eq 2.

Binding of mAB 305-78-7 to MntC-pLH94 was tested using ITC and Enzyme-Linked Immunosorbent Assay (ELISA), as described in Materials and Methods. ITC experiments showed that binding of the wild type MntC to mAB 305-78-7 was stoichiometric (Fig 6A), indicating very high affinity of the interaction. The apparent K_d_ was 1.2 nM, and the binding enthalpy was -19.9 kcal/mol. No binding was observed in the case of MntC-pLH94 (Fig 6B), indicating that amino acid substitutions H234F, E247L and K254M have indeed disrupted the mAB 305-78-7 binding epitope on MntC, as anticipated from the X-ray structure and DXMS data. Additional evidence for disruption of the MntC:mAB 305-78-7 interaction is provided by the ELISA results shown in Fig 7. The dissociation constant of wild type MntC binding to the antibody is in excellent agreement with the ITC result. In contrast, MntC-pLH94 results are essentially identical to those obtained with an unrelated control protein (BSA), once again confirming that the amino acid substitutions H234F, E247L and K254M within the mAB 305-78-7 epitope have indeed disrupted binding.

**Fig 8:**
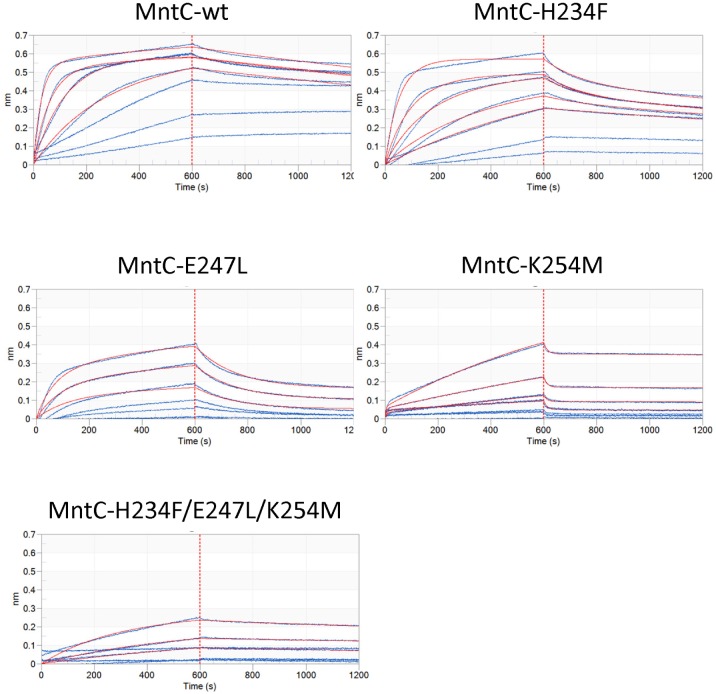
Bio-Layer Interferometry characterization of mAB 305-78-7 binding to MntC variants. Y-axes—magnitude of the interference patterns shifts in nanometers, X-axes—experiment time in seconds. 0 to 600 s—association phase of the experiment, 601–1200 s—dissociation phase of the experiment (separated from the association phase by dashed red lines for clarity). Blue lines—experimental traces of individual antigen concentrations, red lines—fitting results.

To confirm that individual residue-residue interactions seen in the crystal structure indeed play roles in mAB 305-78-7 binding to MntC in solution and to characterize individual contributions of H234, E247L and K254 to the complex formation, we have additionally cloned and expressed a series of MntC variants containing substitutions at those positions. No structural characterization was done for those variants, since no structural changes could be detected in the triple mutant described above, therefore it is reasonable to expect that single mutants would have no effect either. Binding of these mutants to the antibody was characterized using Bio-Layer Interferometry on an Octet HTX instrument, as described in “Materials and Methods”. The results are shown in Table 2 and Fig 8, with Octet data for the triple mutant H234/E247L/K254M included as reference. As can be seen from the results, all of these amino acid substitutions affected binding of mAB 305-78-7 to MntC, albeit to a different degree. K254M substitution disrupted binding almost to the same degree as the three substitutions combined—no rate constants can be derived from the fits of experimental data. Binding of mAB 305-78-7 to the other two mutants, H234F and E247L, can still be detected, however it is clear from Fig 8 that both variants are characterized by significantly increased dissociation rate constants, as compared to the wild type, while the association rate constants remained relatively unaffected. This would translate to the reduced binding affinity, although this is not immediately obvious from Table 2 due to significant experimental variation. Taken together, these results suggest that X-ray crystallography has correctly identified amino acid residues involved in MntC-mAB 305-78-7 interaction.

**Table 2:** Results of the Bio-Layer Interferometry measurements. Rate constants are averages of two independent experiments (as described in “Materials and Methods”). k_on_—association rate constants, k_off_—dissociation rate constants, K_d_—equilibrium dissociation constants. ND—not determined (poor fit of experimental data).

MntC variant	k_on_, M^-1^ s^-1^	k_off_, s^-1^	K_d_, M
Wild type	(2.2±1.4)x10^5^	(2.4±0.7)x10^-4^	(1.2±0.4)x10^-9^
H234F	(2.1±0.6)x10^5^	(3.3±2.8)x10^-4^	(1.4±0.9)x10^-9^
E247L	(3.0±2.8)x10^5^	(7.4±1.4)x10^-3^	(41±34)x10^-9^
K254M	ND	ND	ND
H234F/E247L/K254M	ND	ND	ND

### Manganese binding by MntC complexed with the antibodies

**Fig 9:**
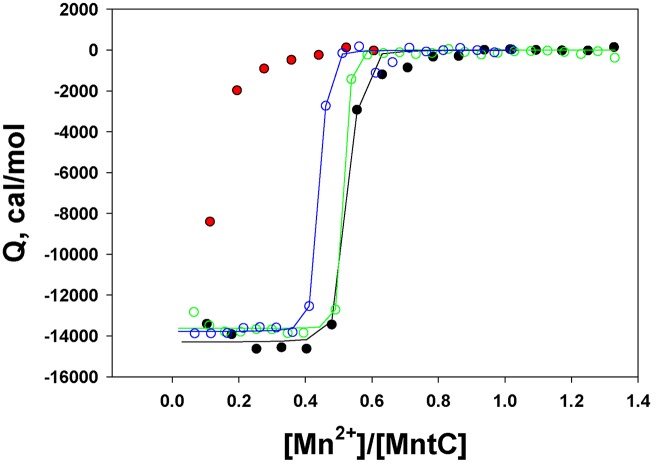
Mn^2+^ titrations of MntC bound to the monoclonal antibodies. The titrations were monitored by ITC, as described in “Materials and Methods”. Black symbols and lines—Mn^2+^ titration of the isolated MntC (control), green symbols and lines—Mn^2+^ titration of MntC bound to mAB 305-72-5, blue symbols and lines—Mn^2+^ titration of MntC bound to mAB 305-101-8, red symbols—Mn^2+^ titration of MntC bound to the Fab fragment of mAB 305-78-7. Binding stoichiometry in all experiments (except for MntC bound to Fab 305-78-8) was 0.42–0.49 Mn^2+^ ions bound per molecule of MntC, consistent with the suggestion that recombinant MntC is partially occupied by an irreversibly bound metal[4]. Binding enthalpies were also comparable (~ -14 kcal/mol).

In an attempt to understand potential mechanisms of MntC-induced resistance to the *S*. *aureus* infection, we studied Mn^2+^ binding to the protein complexed with the antibodies. The results are shown in Fig 9. Because Mn^2+^ binding to MntC is stoichiometric under experimental conditions used, we will not draw conclusions on the association constants derived from these experiments. It should be noted, however, that stoichiometries and binding enthalpies in the experiments with MntC bound to mAB 305-72-5 or mAB 305-101-8 are very comparable to those derived from the control experiment with free MntC, suggesting that these two antibodies do not affect Mn^2+^ binding by the protein. This is not the case with MntC bound to the Fab fragment of mAB 305-78-7. Although the first Mn^2+^ injection does produce a noticeable heat exchange (Fig 9, red symbols), it is clear that mAB 305-78-7 interferes with manganese binding to the protein and no binding parameters can be derived. The ITC titration data suggest that protection mechanism afforded by the anti-MntC monoclonal antibodies can be, at least in part, explained by the interference with Mn^2+^ binding to this metal-binding component of the MntABC transporter and thus preventing Mn^2+^ transport into the bacterial cytoplasm.

## Discussion

In the current work, we mapped binding epitopes of the representative monoclonal antibodies from different interference groups reported earlier [3]. As expected for the protective antibodies [10,11], all three epitopes were conformational. The DXMS data clearly show that the mAB 305-72-5 binding epitope (interference group 1) involves two discontinuous segments. In the case of the other two antibodies one could argue (based strictly on DXMS results) that the epitopes could be linear: Our DXMS data did not provide sufficient resolution in those two cases. The X-ray structure of MntC in complex with mAB 305-78-7 (interference group 2), however, shows that there are several other isolated residues outside of the linear peptide 243–260 that are involved in the interaction, confirming that mAB 305-78-7 epitope is discontinuous, as well. To provide additional evidence for the conformational nature of the epitopes recognized by the antibodies mAB 305-78-7 and mAB 305-101-8 (interference group 3), we conducted ITC titrations of the antibodies under study with synthetic linear peptides derived from the amino acid sequences of the structural segments identified using DXMS. No binding could be detected in these experiments (S6 Fig). These results confirm that the unique spatial arrangement of the residues dictated by the 3-dimensional folding of the identified peptide sequences is important for the antibody binding.

**Fig 10:**
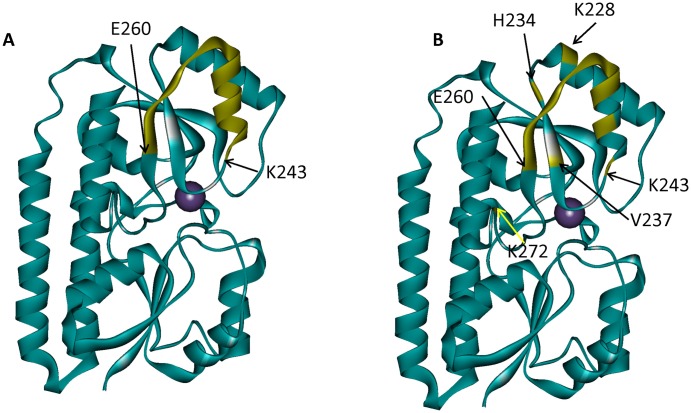
Comparison of mAB 305-78-7 binding epitopes identified using DXMS and X-ray crystallography. *A*–DXMS results. Peptide K243-E260 which shows pronounced decrease in deuterium accumulation in the presence of the antibody is highlighted on MntC structure in yellow. *B*–X-Ray crystallography results. MntC residues located within 4 Å of the antibody (listed in Table 1) are highlighted on MntC structure in yellow.

Detailed comparison of the mAB 305-78-7 epitope mapped via DXMS and X-ray shows that both methods produce very similar results: the major binding surface is formed by the α-helix α8, β-sheet β8, and the loop connecting these two structural elements (Fig 10). Based on the available DXMS data, we concluded that the epitope is located between residues 243 and 260. Analysis of the X-ray structure leads to essentially the same conclusion: residues 243, 247 and 250–260 are all parts of the structural segment identified using DXMS. In addition, the X-ray structure shows that residues 228, 234, 237 and 272 may provide additional interactions that stabilize the complex. The DXMS data do show some differences in deuterium accumulation in the peptides that include those four residues, but we deemed those differences too small and inconsistent to say with confidence that these peptides originated from the part of the protein that is involved in the antibody binding. This observation illustrates the power of X-ray crystallography to provide residue-specific information with regards to the composition of the binding interface. However, DXMS data were obtained at a fraction of the cost and effort required for crystallographic analysis, yet still provided largely comparable results.

The MntC variant pLH94 (H234F, E247L/K254M) was designed to further confirm mAB 305-78-7 epitope identification. We have chosen two residues that could potentially form intermolecular hydrogen bonds (H234, and K254), along with a residue that appears to form an intermolecular salt bridge (E247). Our goal was to minimize the number of amino acid substitutions to minimize any potential disruption or alteration of MntC structure, hence only three out of five residues with side chains involved in intermolecular interactions were selected. Substitutions at these three positions were sufficient to completely disrupt binding, as evidenced by the ITC and ELISA results (Figs 6 and 7). To ensure that MntC-pLH94 was unable to bind mAB 305-78-7 due to the targeted replacements and not due to the disruption of the structure, we assessed secondary and tertiary structure of the mutant protein using CD spectroscopy. According to the CD data (S4 Fig), amino acid substitutions H234F, E247L and K254M had no detectable effect on secondary and tertiary structure of the protein (changes in spectral intensity observed between ~260–275 nm in the near-UV spectrum likely arise due to the introduction of an additional phenylalanine residue as a result of H234F substitution and do not necessarily reflect global structural changes), since CD spectra of the mutant are identical to the spectra of the wild type protein. In addition, MntC-pLH94 is capable of Mn^2+^ binding with the same thermodynamic parameters as the wild type protein (S3 Table). Taken together the CD, ITC and ELISA results show that amino acid substitutions H234F, E247L and K254M had little to no effect on the global structure of the protein, while at the same time disrupted mAB 305-78-7 binding to the protein. Finally, results from the Bio-Layer Interferometry experiments demonstrated that each of those residues contributes to the interaction with the antibody, although to a different degree. Results from these experiments, therefore, confirm that mAB 305-78-7 binding epitope has been correctly identified.

The results reported in this work shed light on the molecular mechanism of protection afforded by the anti-MntC antibodies. Another report from our laboratories [1] demonstrated that mutations in MntC gene make invasive *S*.*aureus* strains susceptible to the oxidative stress. Considering that manganese, which is the metal transported by MntC, is an essential co-factor of superoxide dismutases, we hypothesized that antibody binding could inhibit MntC-Mn^2+^ interaction or interfere with Mn^2+^ transport through the channel pore, making bacteria more susceptible to the attack by neutrophils. A recently published paper by Ahuja and co-workers [34] provided evidence that monoclonal antibody fragment FabC1 indeed interferes with Mn^2+^ transfer from MntC to the importer MntB. FabC1 and our monoclonal antibody mAB-101-8 have overlapping epitopes, thus, they both belong to the interference group 3 as defined by Anderson et al.[3]: residues 210–220 that show decreased deuterium accumulation in the presence of mAB-101-8 in our experiments include part of the α-helix 3 from the C-terminal lobe of MntC (which corresponds to the α-helix 7 in our nomenclature) shown to be involved in FabC1 binding [34]. Thus it is very likely that mAB 305-101-8 (and any other antibodies belonging to the interference group 3) also sterically interferes with MntC-MntB interaction, preventing Mn^2+^ release into the channel pore. mAB-305-72-5 binding epitope lies on the same face of MntC molecule as that of mAB-101-8, although on the N-terminal lobe of the structure. Binding of the antibody to this site therefore would also be expected to sterically interfere with MntC-MntB interaction, similar to mAB 305-101-8 or FabC1 interaction, once again preventing Mn^2+^ transfer into the channel pore.

Our X-ray crystallography data published earlier [4] showed that two molecules of MntC are present in the asymmetric unit of the crystal, with one of the molecules completely missing electron density for residues 240–258. DXMS and X-ray crystallography data reported here identified these residues as part of mAB 305-78-7 binding epitope. Disordering of these residues made Mn^2+^ binding site solvent accessible, while folding of these residues into an alpha-helix in another molecule of the asymmetric unit blocked solvent accessibility. It is tempting to speculate that binding of mAB 305-78-7 to residues 240–258 would stabilize the alpha-helix and, correspondingly, prevent binding of Mn^2+^ to the protein. This is indeed what seems to be happening. ITC titration of MntC bound to the Fab fragment of 305-78-7 (Fig 9) showed that the antibody strongly interferes with (if not completely abolishes) Mn^2+^ binding to the protein—initial heat exchange could arise from Mn^2+^ binding to a small fraction of the free protein in equilibrium with MntC-Fab complex.

Taken together, experimental data reported here, along with results from Anderson et al.[3], Handke et al.[1] and Ahuja et al.[34] provide a plausible description of immunogenic properties of MntC and explain potential mechanism of protection afforded by the MntC-induced antibodies. Interference mapping [3] established that there are three non-interfering groups of the monoclonal mABs. DXMS and X-ray crystallography mapping of the epitopes recognized by selected representatives of each groups allowed us and others [34] to hypothesize that there are at least two potential mechanisms that can explain protection afforded by these antibodies. mAB’s belonging to the interference group 2 (e.g., mAB 305-78-7) block Mn^2+^ binding to MntC, making it impossible for *S*.*aureus* to acquire this critical metal. Antibodies belonging to the interference groups 1 (e.g., mAB 305-72-5) and 3 (e.g., mAB 305-101-8 and FabC1) prevent transport of the Mn^2+^ ions that were already acquired across the bacterial membrane. The net result of the impaired manganese transport would be reduced activity of superoxide dismutase and increased susceptibility of the pathogen to oxidative stress that the organism will encounter after opsonophagocytosis by the neutrophils. The protective antibodies that belong to these interference groups will effectively starve the bacterium of Mn^2+^ that it needs for survival.

In conclusion, we would like to add that the results reported in this work provide deeper understanding of the MntC antigen mechanism of action within SA4Ag. Investigational clinical studies demonstrated that antibodies that compete with these functional monoclonal antibodies are not detected in humans without documented *S*.*aureus* infection, however they are rapidly generated as a result upon infection indicating that the antigen is expressed and exposed early in infection [47]. Identification of the protective epitopes reported in this study is therefore useful for the characterization of potential protective antibody responses induced by vaccines that are in clinical development.

## Supporting Information

S1 Fig Comparison of the deuterium accumulation plots obtained with MntC alone or in complex with mAB 305-78-7. X-axis—time, y-axis—number of deuterons exchanged at a given time point. The plots are scaled to the maximum number of deuterons that can be exchanged onto the backbone of the peptide. Blue symbols and lines—deuterium accumulation on MntC alone, red symbols and lines—deuterium accumulation on MntC in the presence of the antibody. When available, multiple plots shown for the same peptide correspond to the different charge states of the peptide.
(PDF)

S2 Fig Comparison of the deuterium accumulation plots obtained with MntC alone or in complex with mAB 305-101-8. X-axis—time, y-axis—number of deuterons exchanged at a given time point. The plots are scaled to the maximum number of deuterons that can be exchanged onto the backbone of the peptide. Blue symbols and lines—deuterium accumulation on MntC alone, red symbols and lines—deuterium accumulation on MntC in the presence of the antibody. When available, multiple plots shown for the same peptide correspond to the different charge states of the peptide.
(PDF)

S3 Fig Comparison of the deuterium accumulation plots obtained with MntC alone or in complex with mAB 305-72-5. X-axis—time, y-axis—number of deuterons exchanged at a given time point. The plots are scaled to the maximum number of deuterons that can be exchanged onto the backbone of the peptide. Blue symbols and lines—deuterium accumulation on MntC alone, red symbols and lines—deuterium accumulation on MntC in the presence of the antibody. When available, multiple plots shown for the same peptide correspond to the different charge states of the peptide.
(PDF)

S4 Fig Circular Dichroism Spectra of wild type MntC and MntC-pLH94. Far-UV (panel A) and near-UV (panel B) CD spectra. Black—spectra of the wild type protein, red—spectra of MntC-pLH94.
(PDF)

S5 Fig Binding of Mn^2+^ to wild type MntC and MntC-pLH94 monitored by ITC. Mn^2+^ binding studies conducted with wild type MntC (Panel A) and MntC-pLH94 (Panel B) are illustrated. Upper panels show experimental heat flow and lower panels show the integrated heat of each individual injection (symbols). Solid lines in the lower panels show fits of the experimental data to the “single class of binding sites” model.
(PDF)

S6 Fig Titration of the monoclonal antibodies 305-78-7 and 305-101-8 with synthetic peptides derived from the sequences of the identified epitopes. *Panel A*–experiments with mAB 305-78-7, *panel B*–experiments with mAB 305-101-8. Open circles: integrated heats of the corresponding peptide injections into the ITC cell containing an appropriate antibody, filled circles—integrated heats of the full length MntC injections into the ITC cell containing either mAB 305-78-7 or mAB 305-101-8. Solid lines—fits to the “single class of binding sites” model. mAB 305-78-7 titration with the synthetic peptide was aborted after 7 injections when it became evident that no heat exchange and, therefore, no interaction is taking place.
(PDF)

S1 Table Crystalographic data collection and refinement statistics. (PDF)

S2 Table Sequences of oligonucleotides used in the study. (PDF)

S3 Table Thermodynamic parameters of Mn^2+^ binding to the wild type MntC and MntC-pLH94. N—interaction stoichiometry, K_a_—association constant, ΔH—enthalpy change upon binding, ΔS—entropy change upon binding, K_d_—dissociation constant.
(PDF)
