# Supplementary material for: High Resolution Mapping of Bactericidal Monoclonal Antibody Binding Epitopes on Staphylococcus aureus Antigen MntC
Source: PLoS Pathog. 2016 Sep 30;12(9):e1005908. doi: 10.1371/journal.ppat.1005908 (PMC5045189; doi:10.1371/journal.ppat.1005908)
Supplement: S2 Fig — X-axis—time, y-axis—number of deuterons exchanged at a given time point. The plots are scaled to the maximum number of deuterons that can be exchanged onto the backbone of the peptide. Blue symbols and lines—deuterium accumulation on MntC alone, red symbols and lines—deuterium accumulation on MntC in the presence of the antibody. When available, multiple plots shown for the same peptide correspond to the different charge states of the peptide. (PDF) [file ppat.1005908.s002.pdf]

SUPPLEMENTARY FIGURE 2

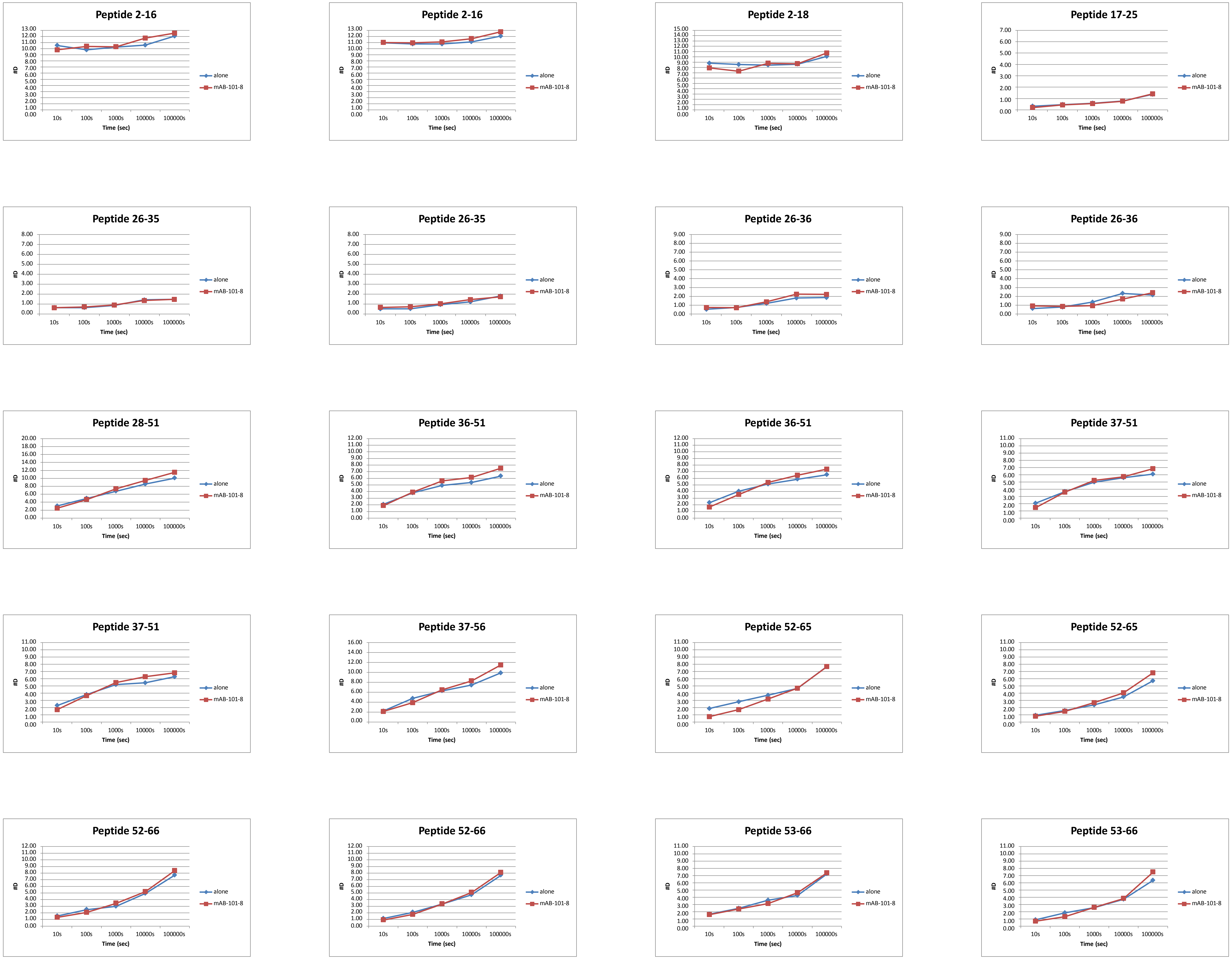

SUPPLEMENTARY FIGURE 2 (CONTINUED)

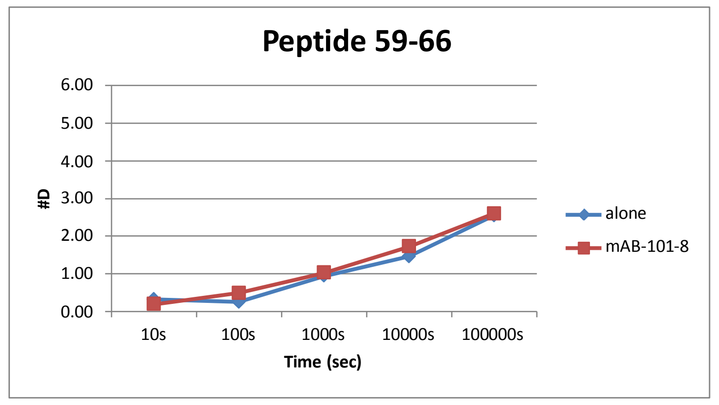

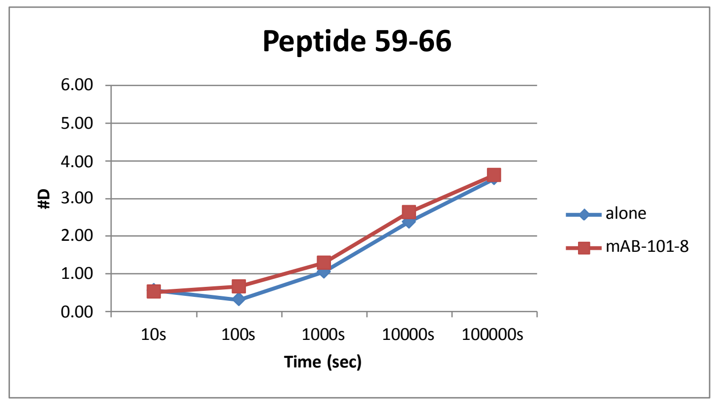

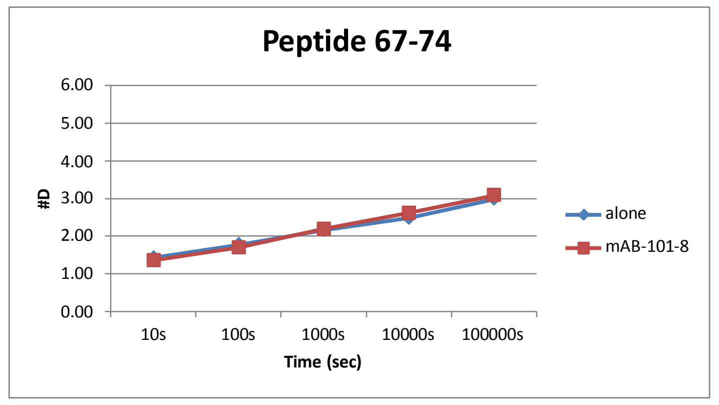

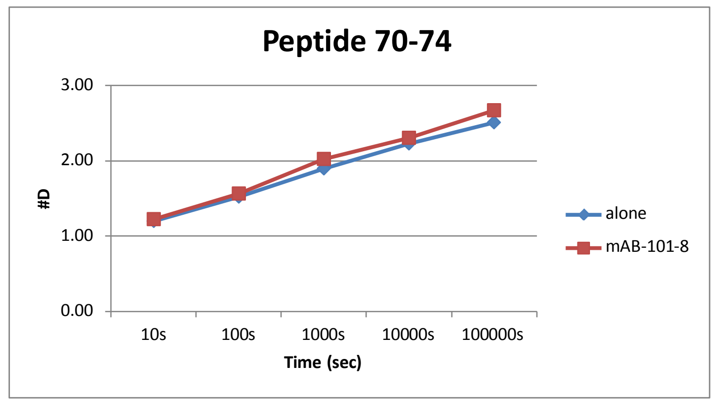

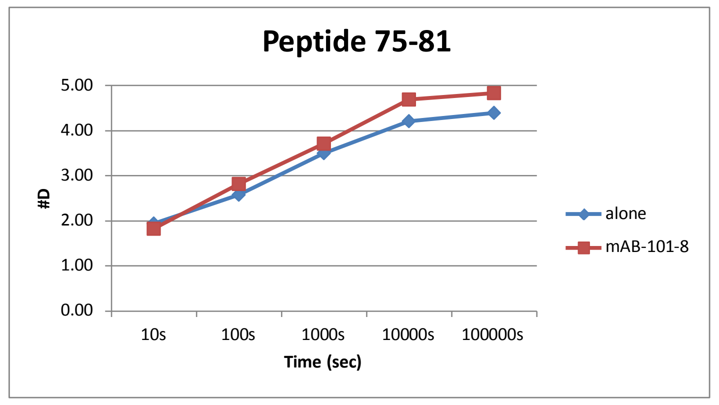

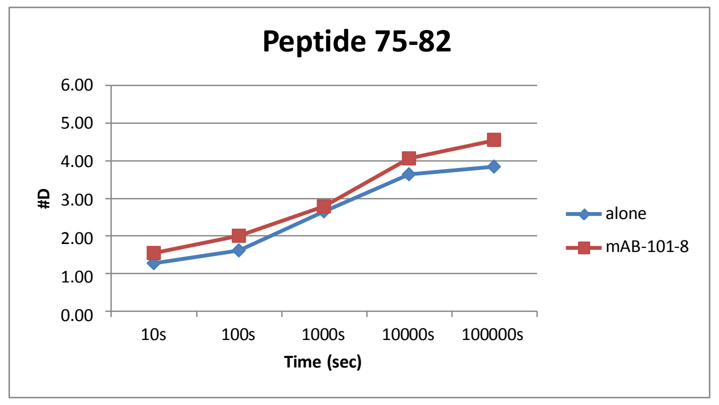

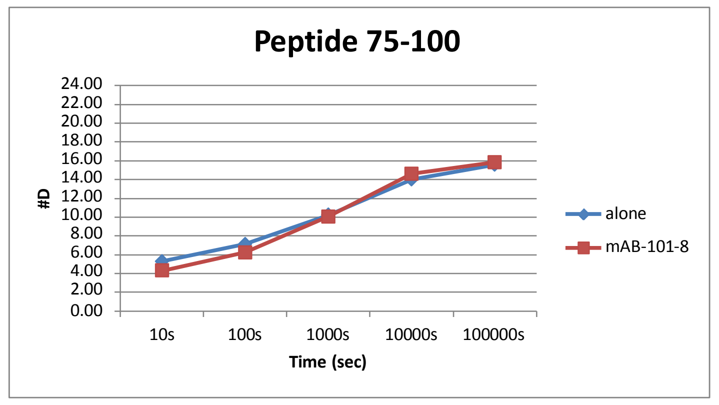

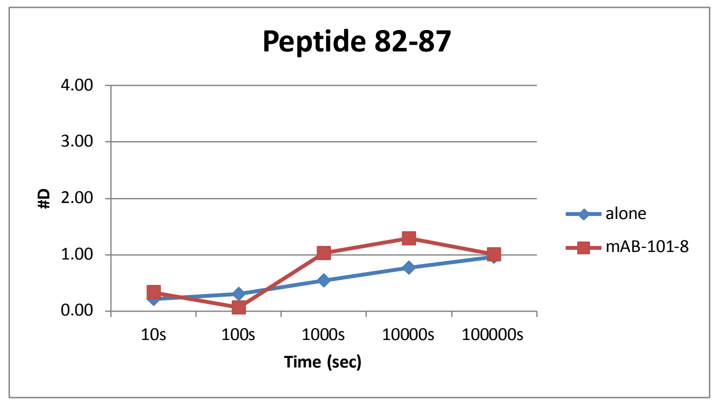

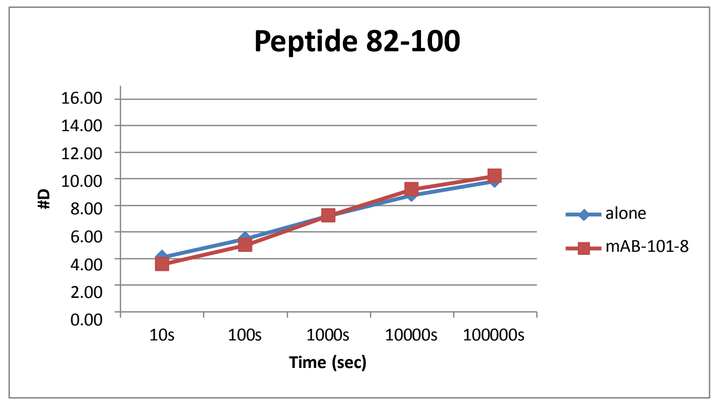

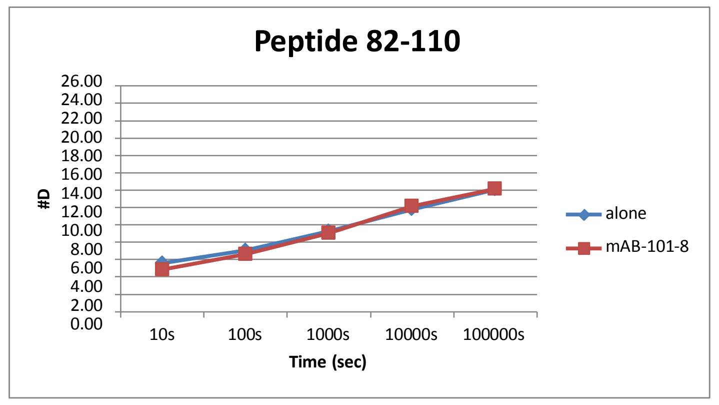

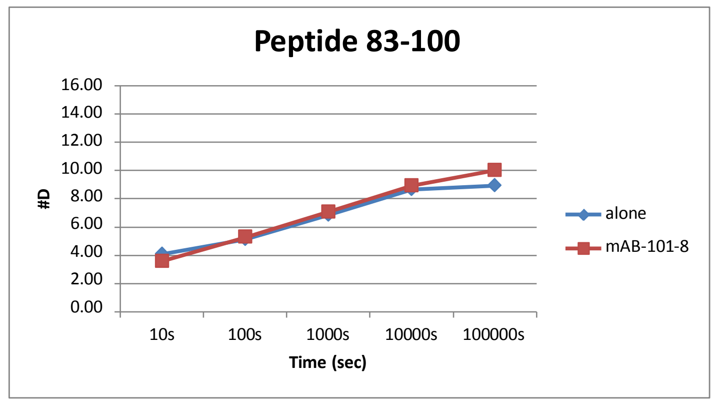

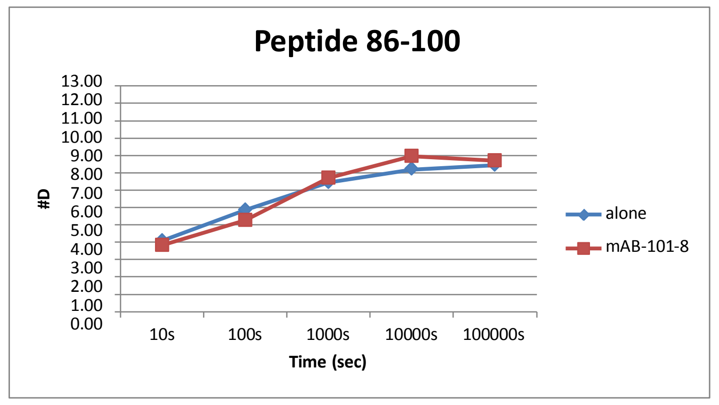

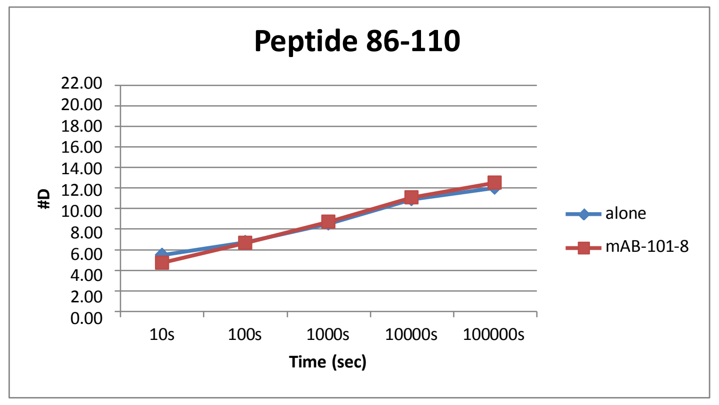

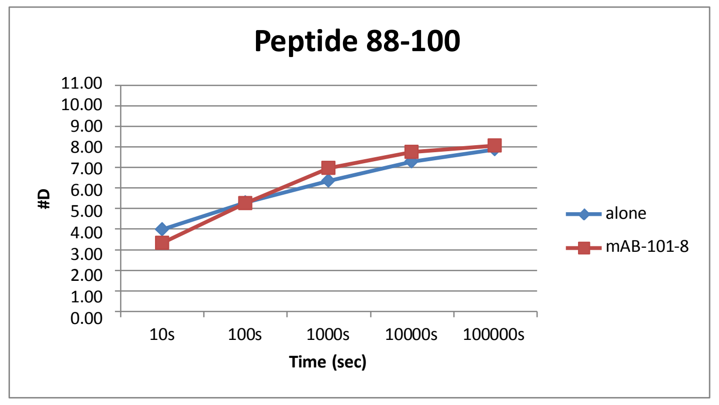

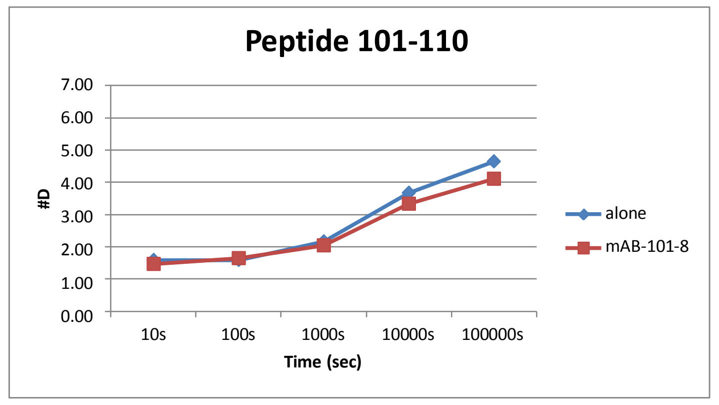

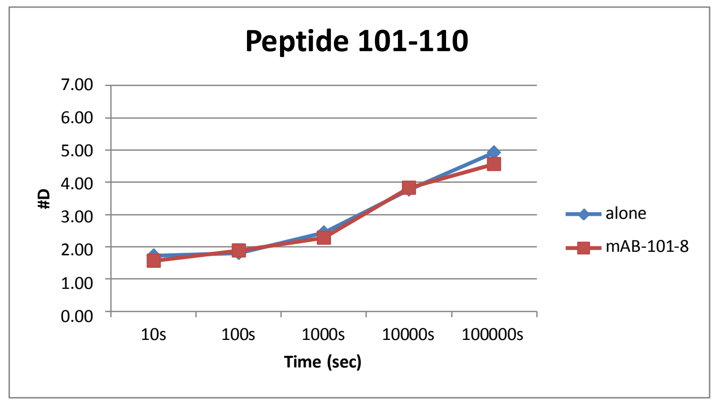

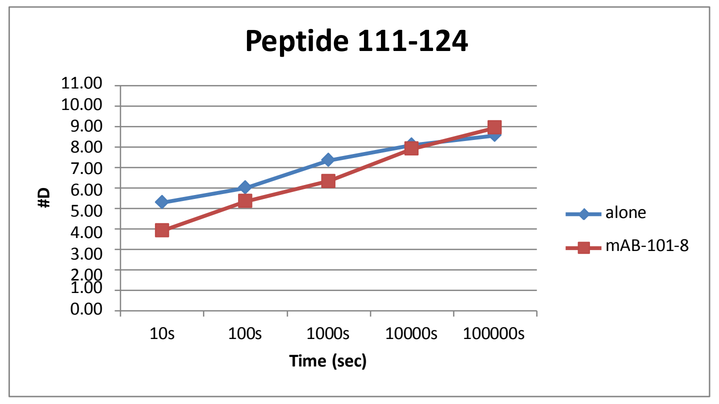

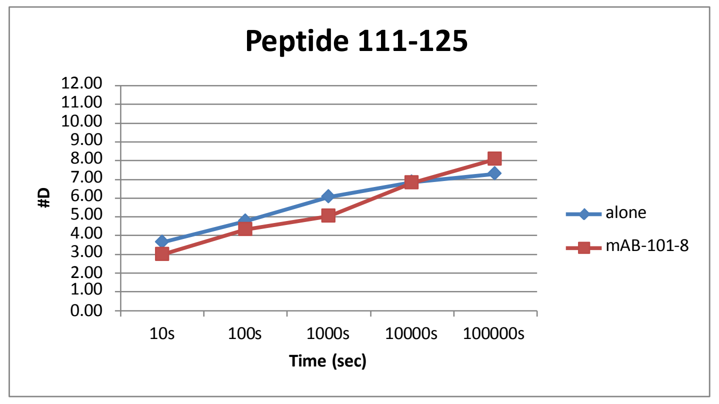

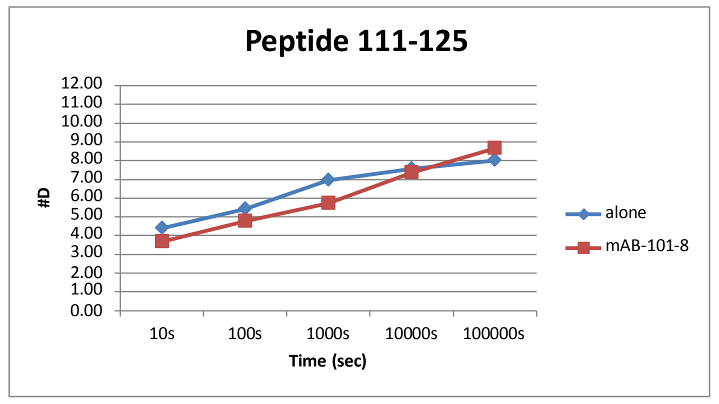

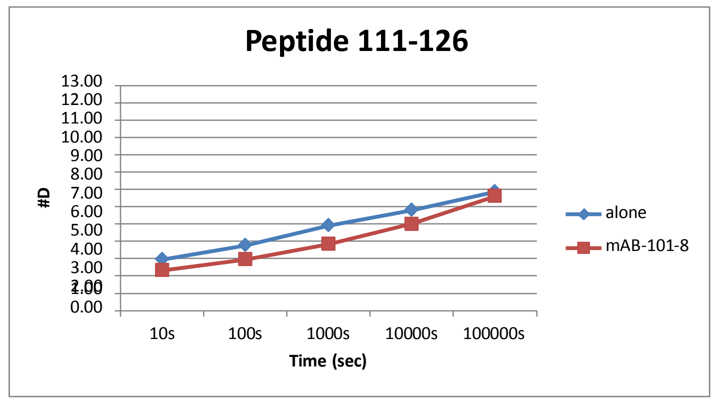

SUPPLEMENTARY FIGURE 2 (CONTINUED)

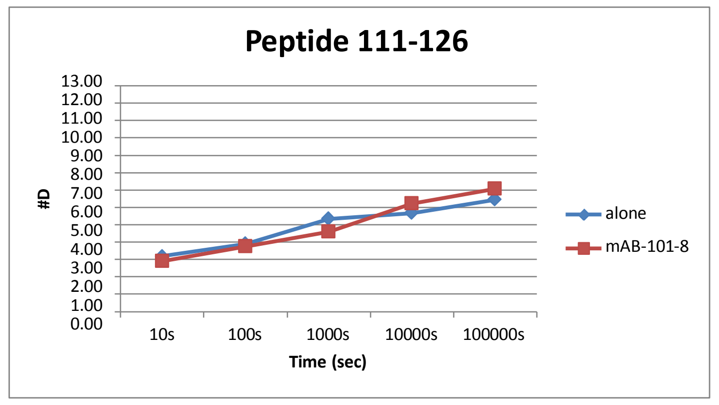

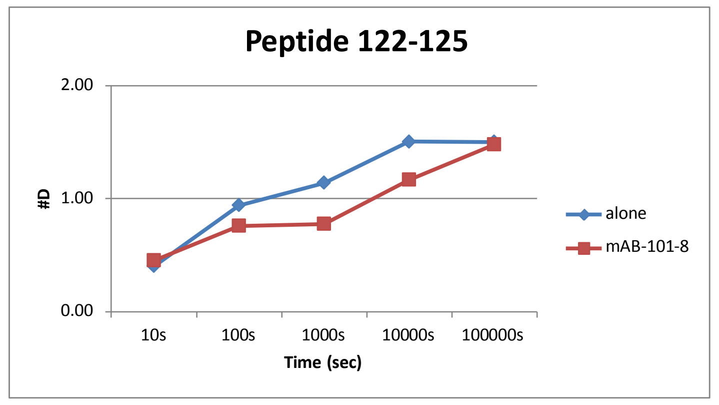

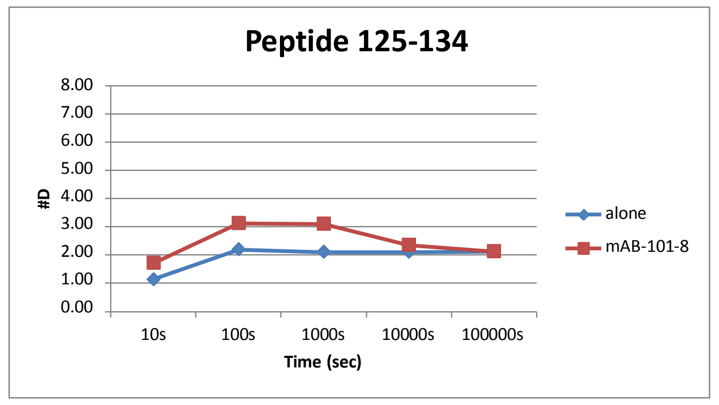

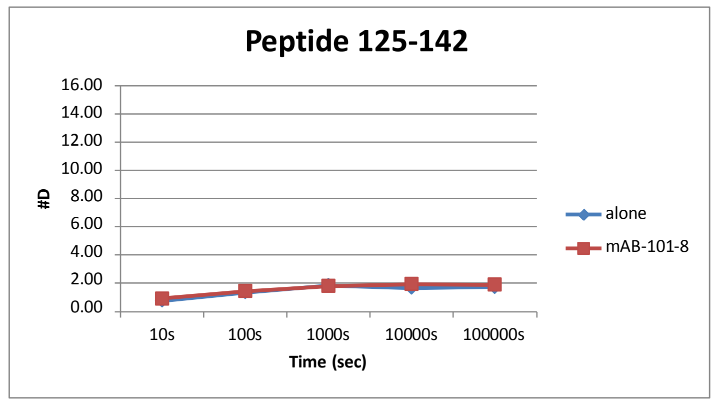

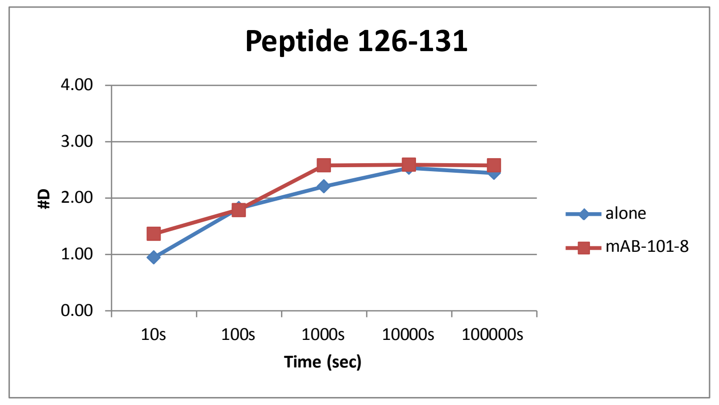

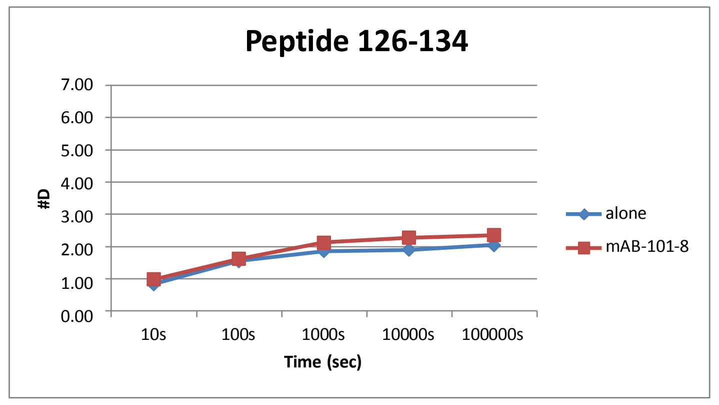

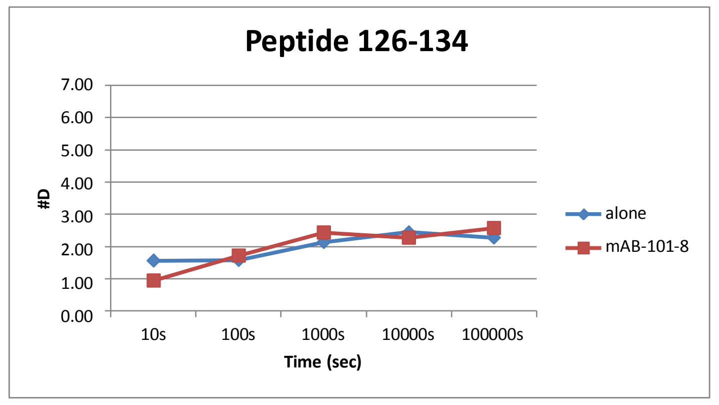

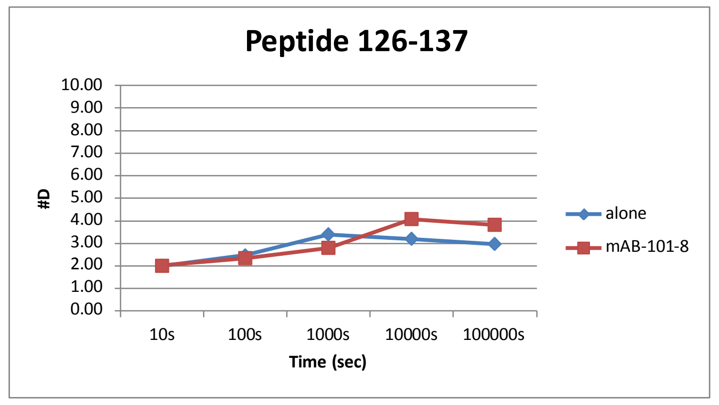

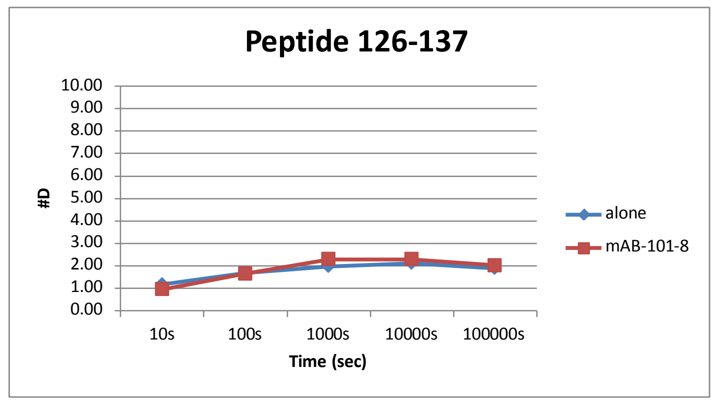

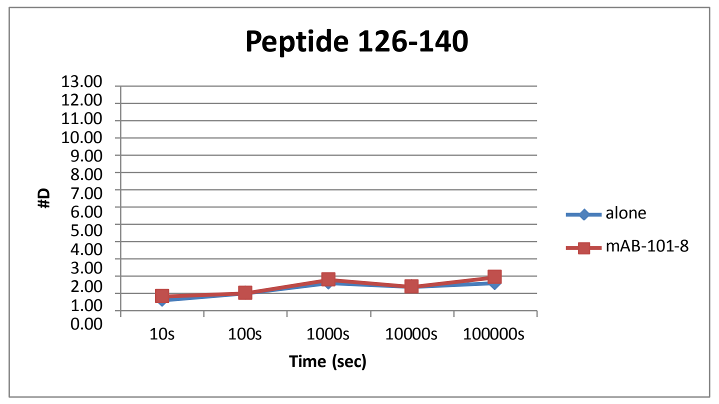

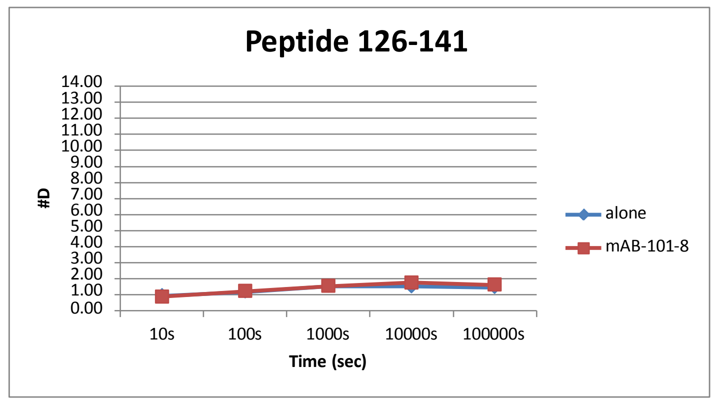

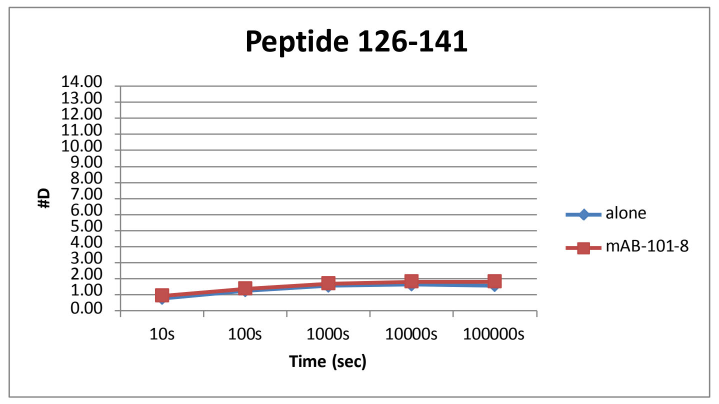

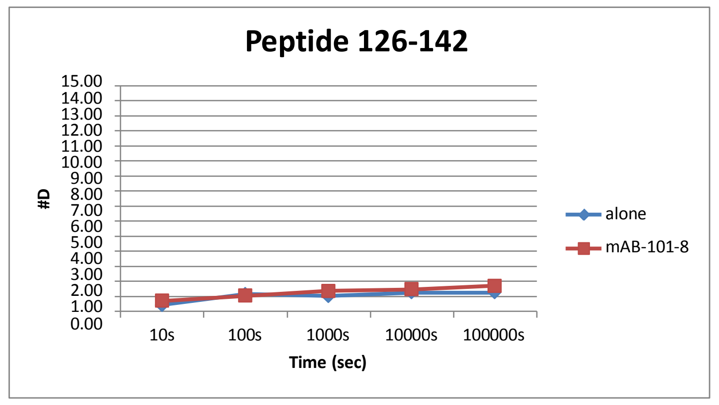

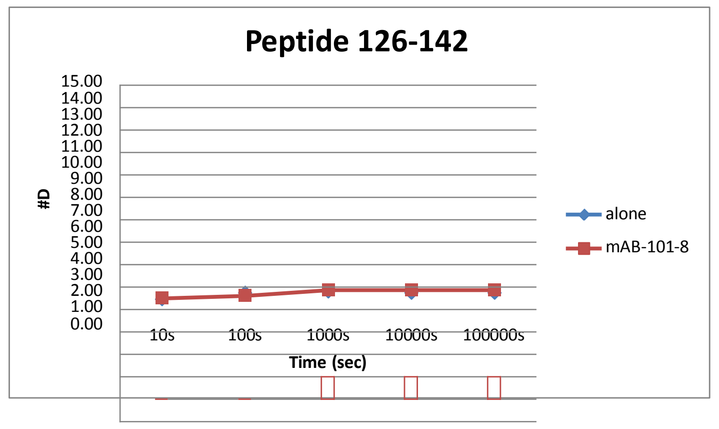

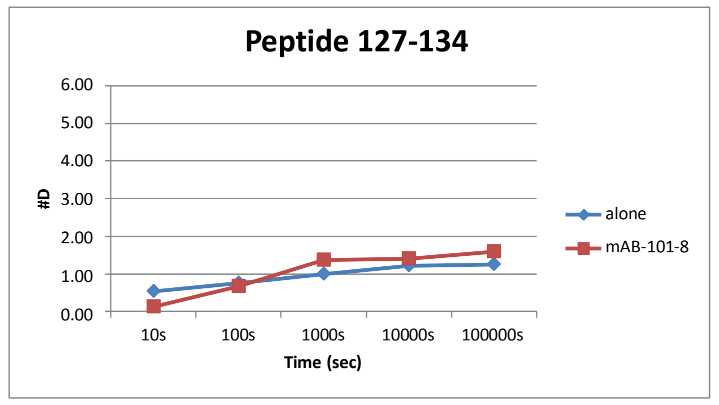

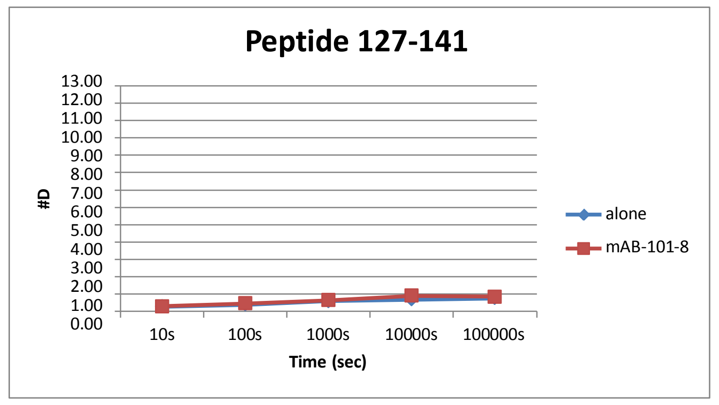

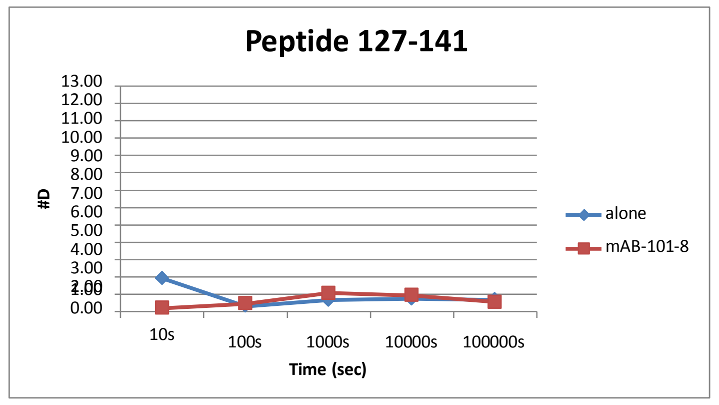

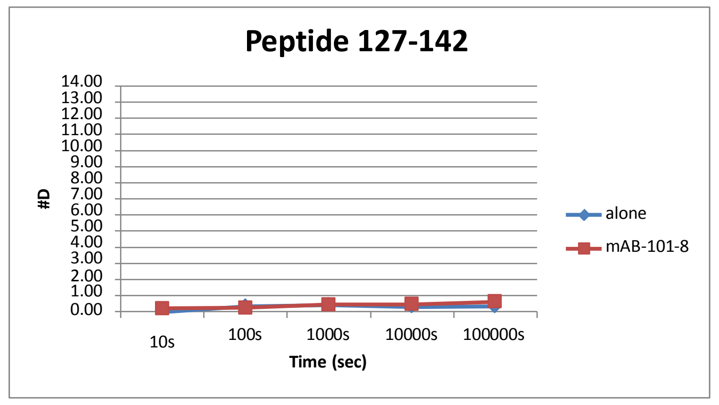

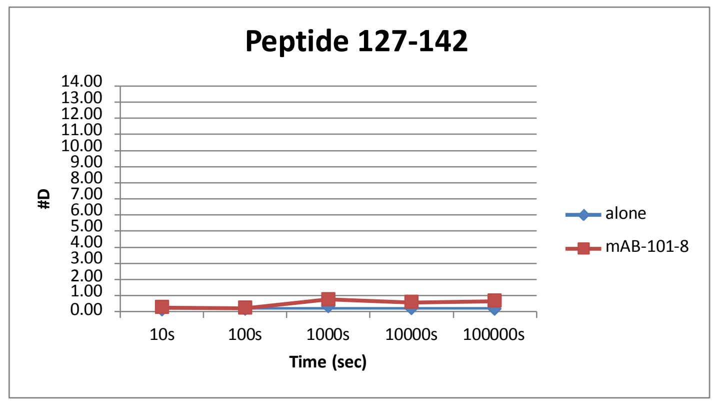

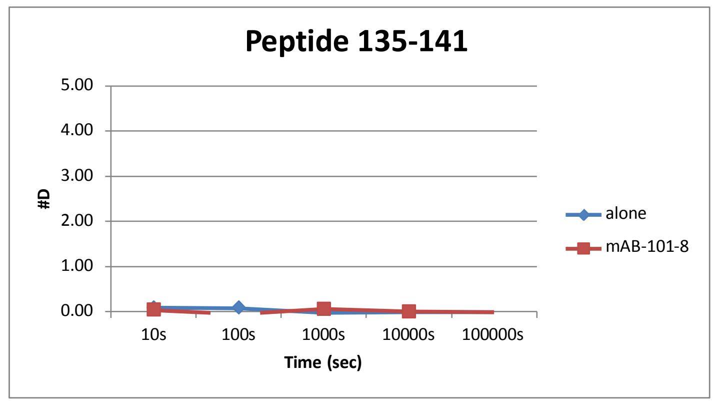

SUPPLEMENTARY FIGURE 2 (CONTINUED)

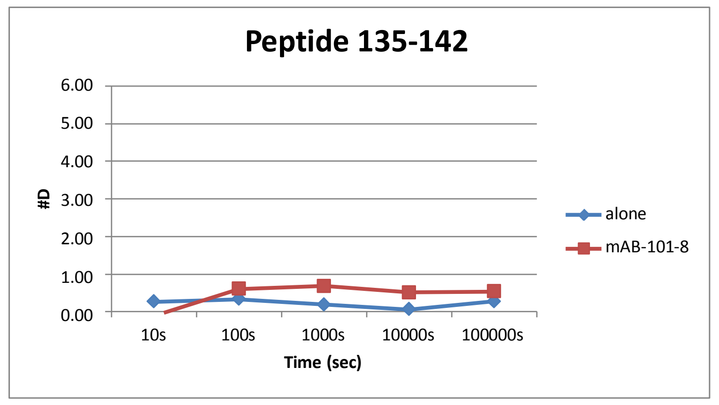

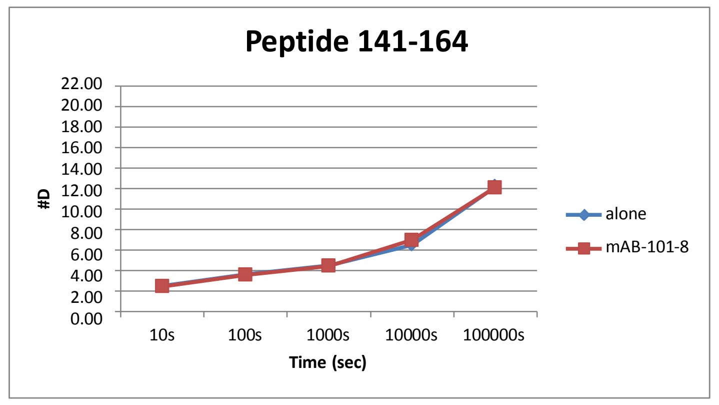

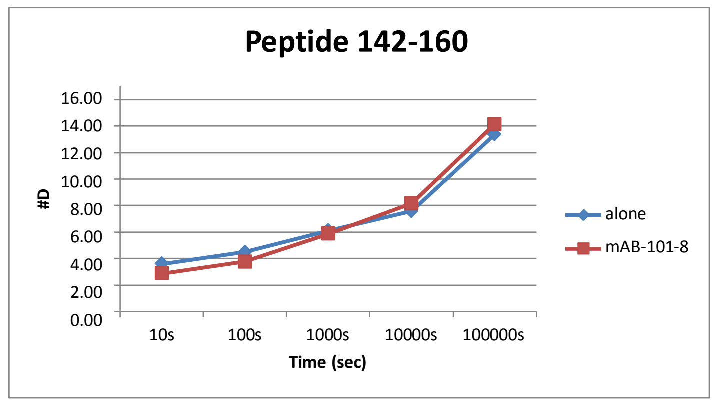

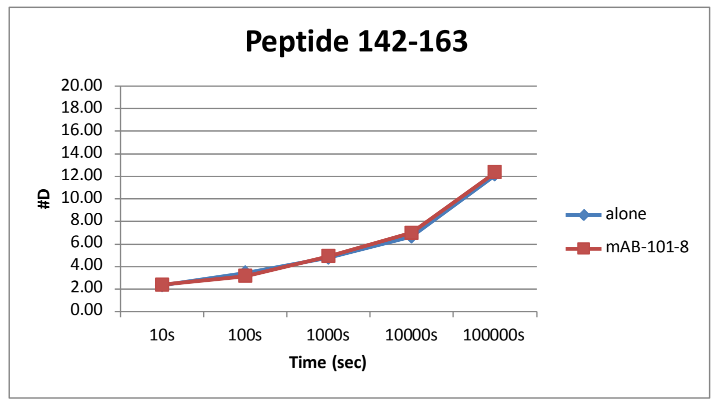

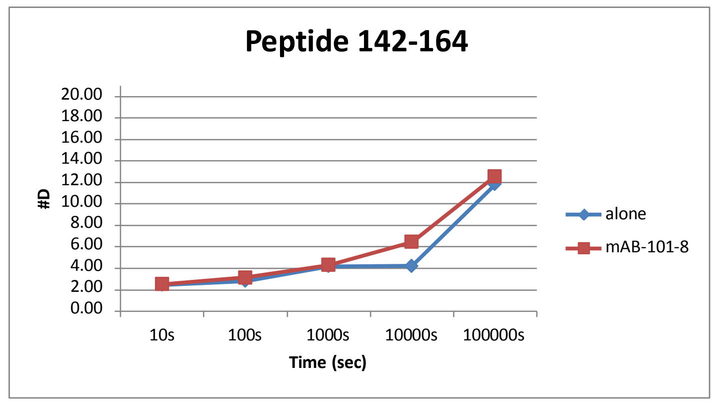

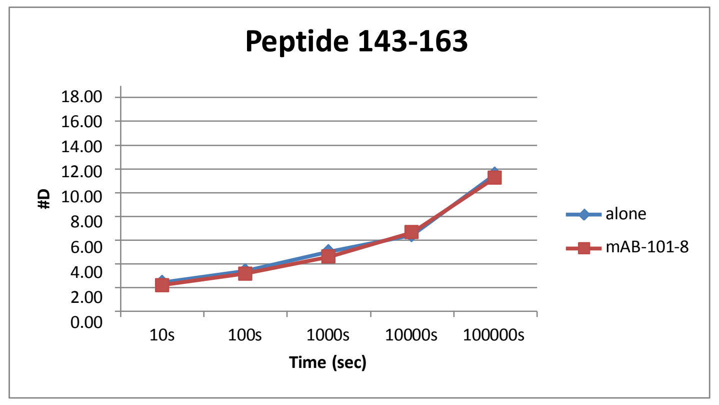

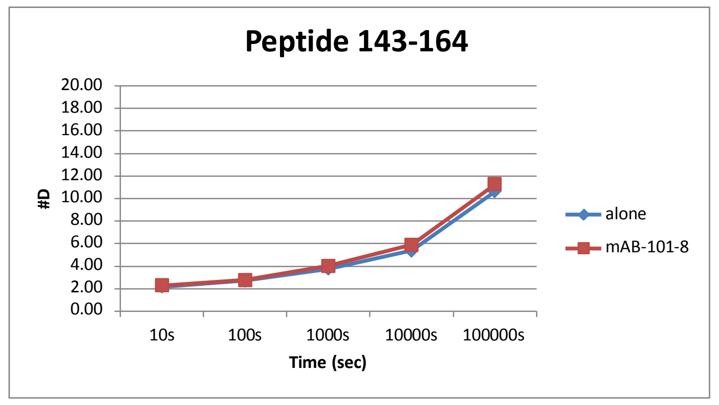

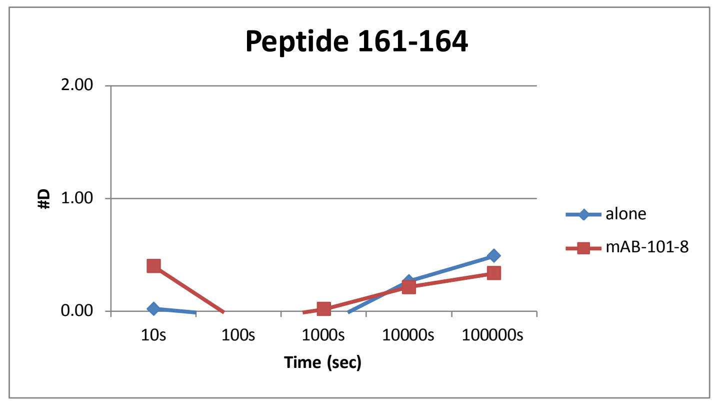

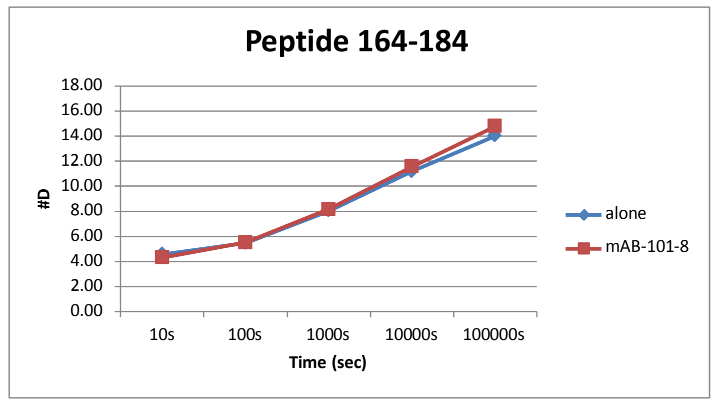

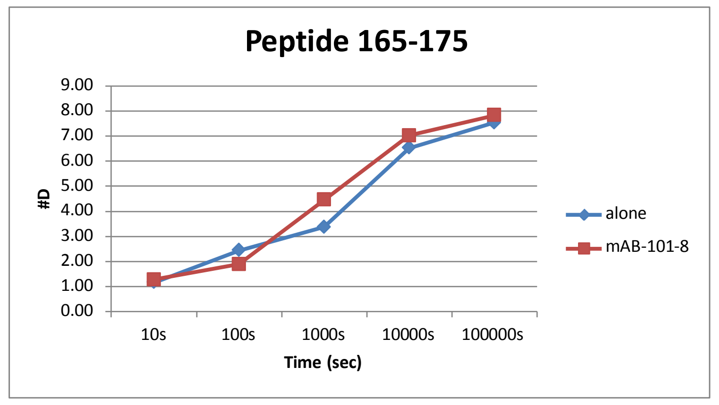

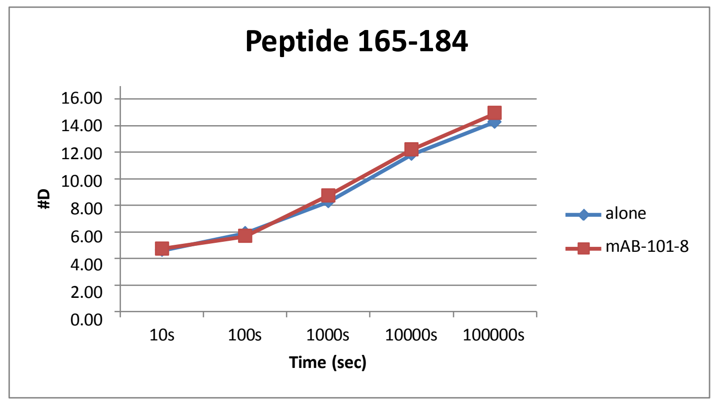

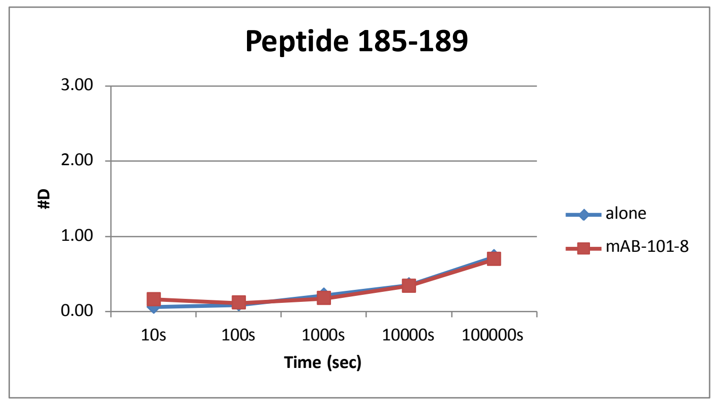

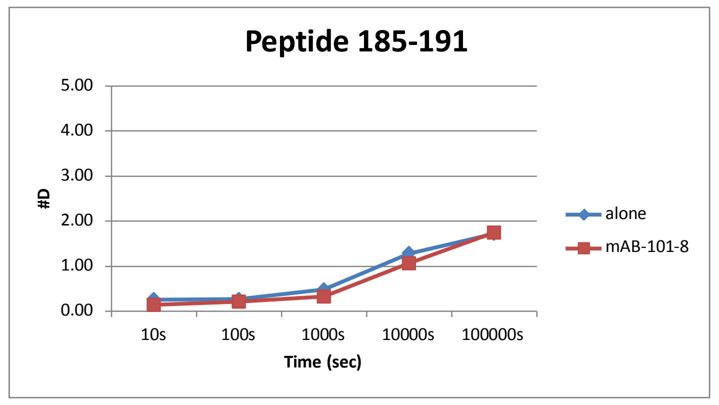

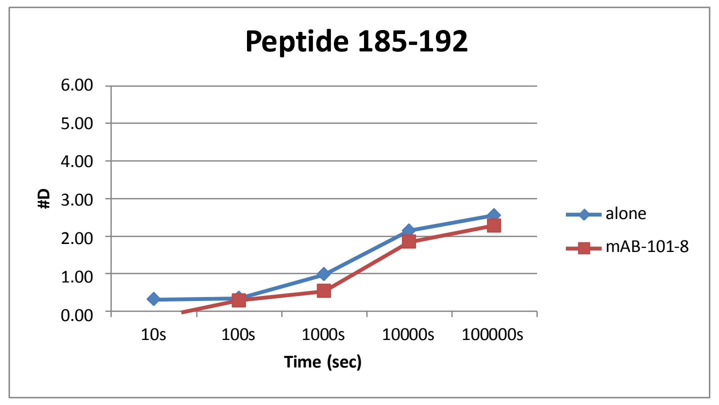

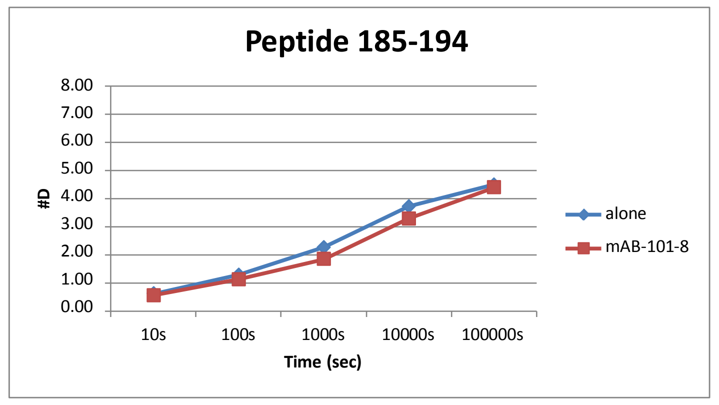

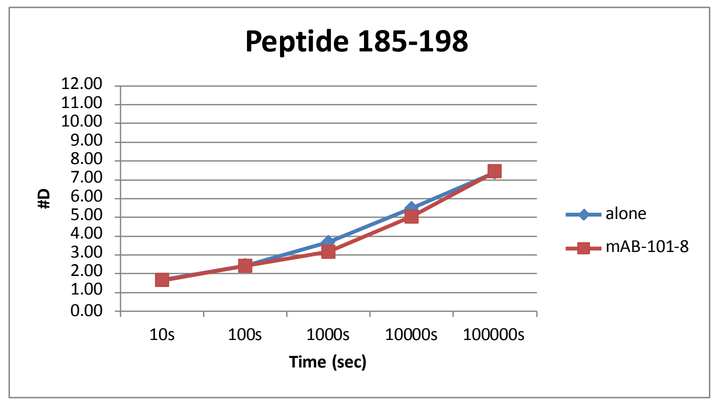

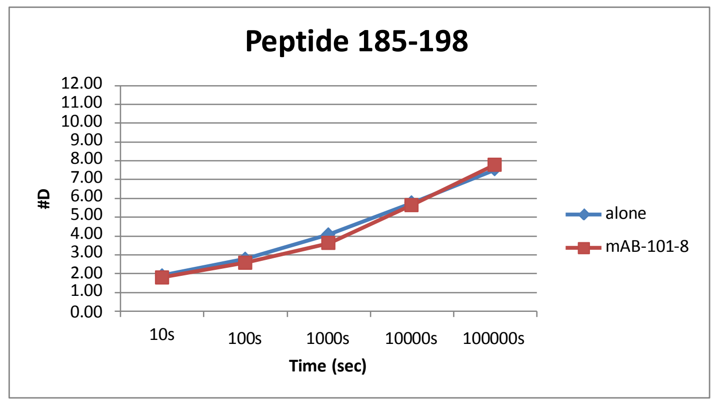

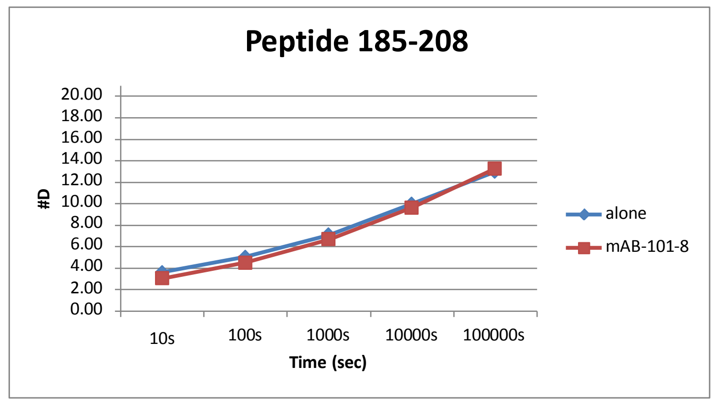

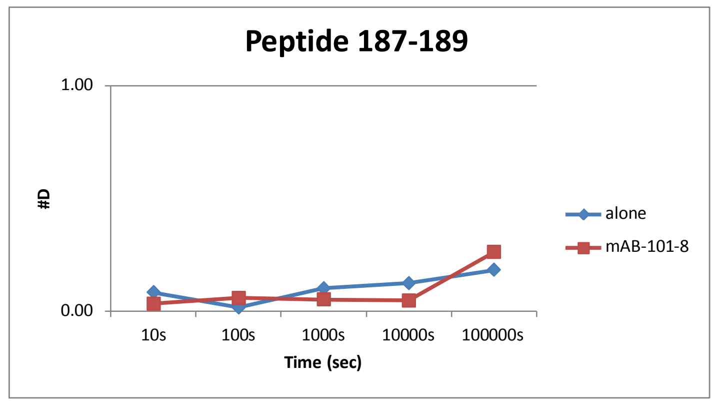

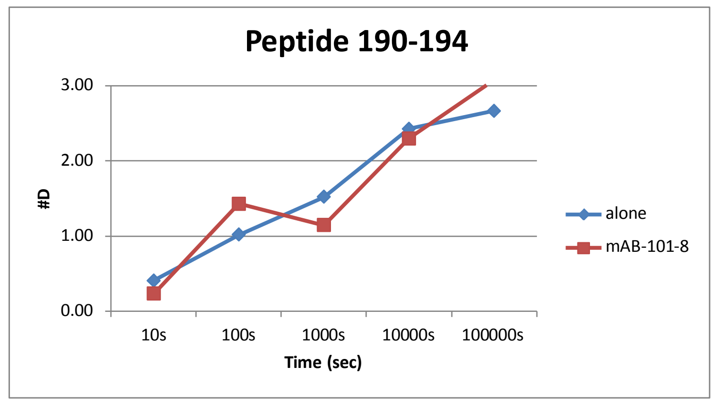

SUPPLEMENTARY FIGURE 2 (CONTINUED)

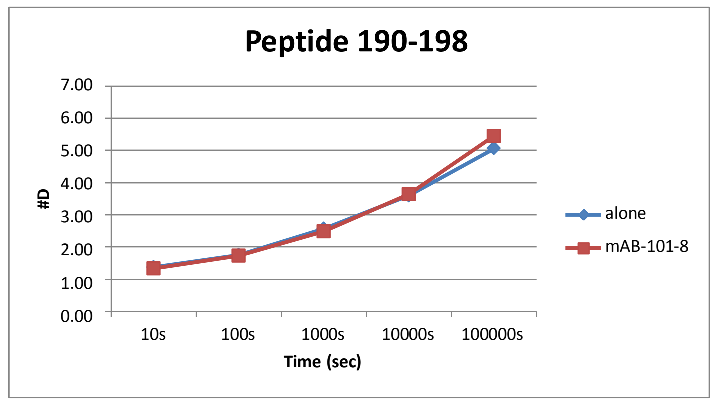

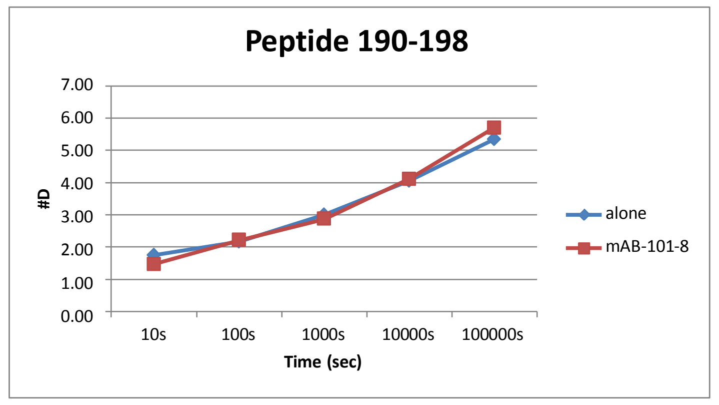

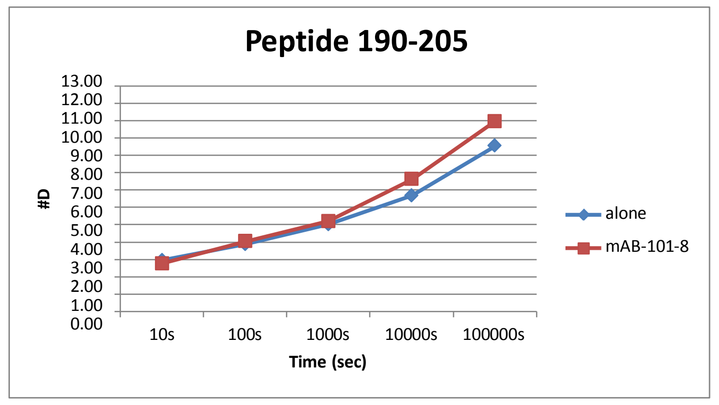

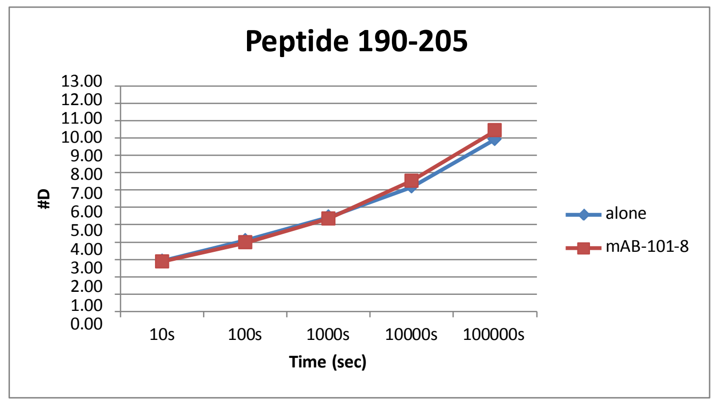

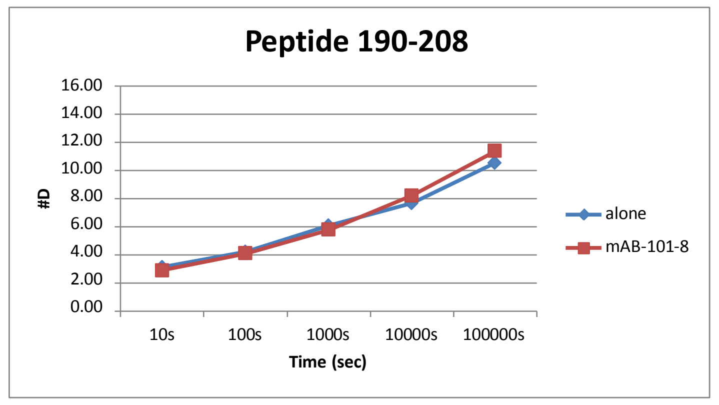

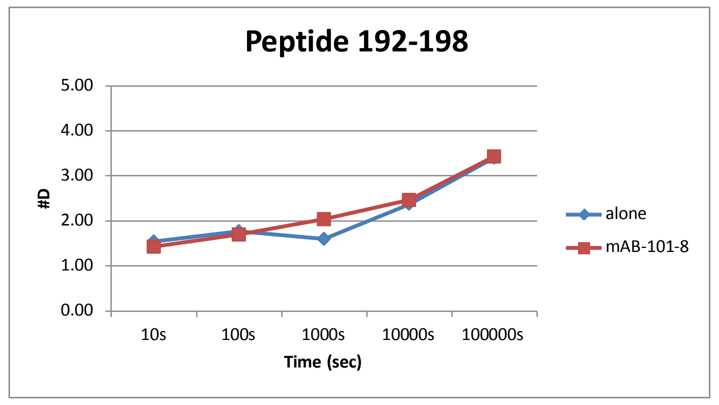

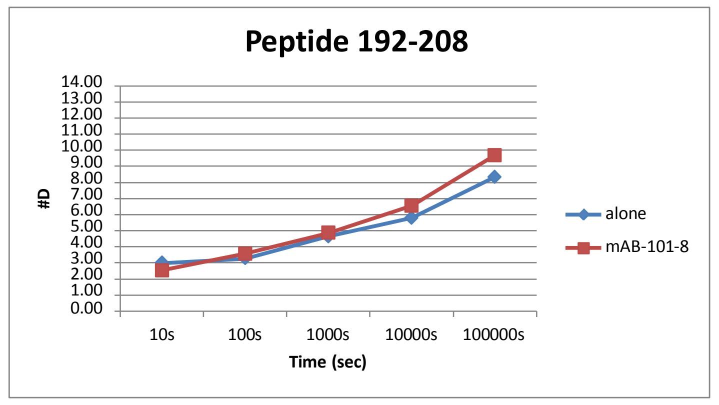

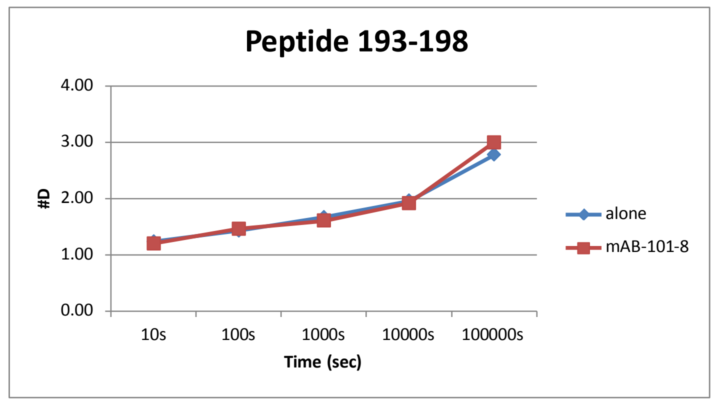

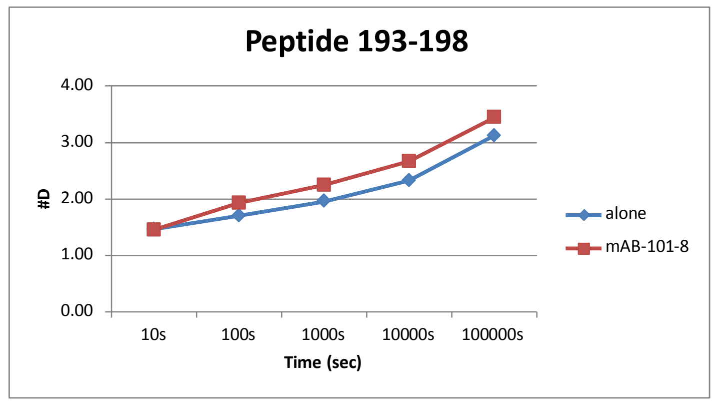

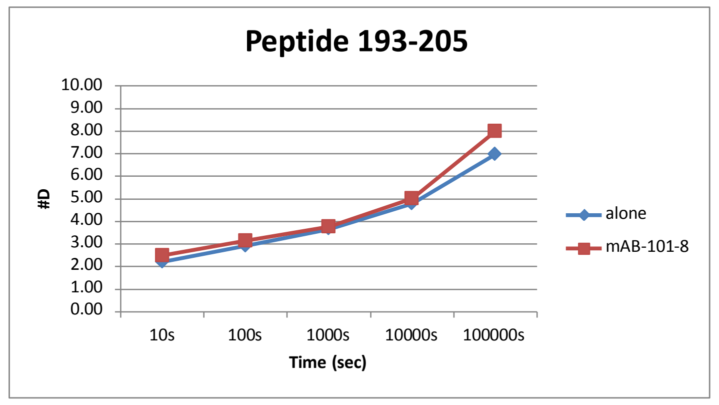

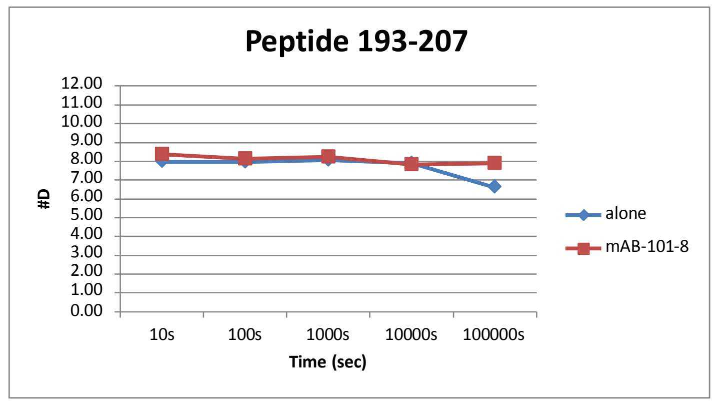

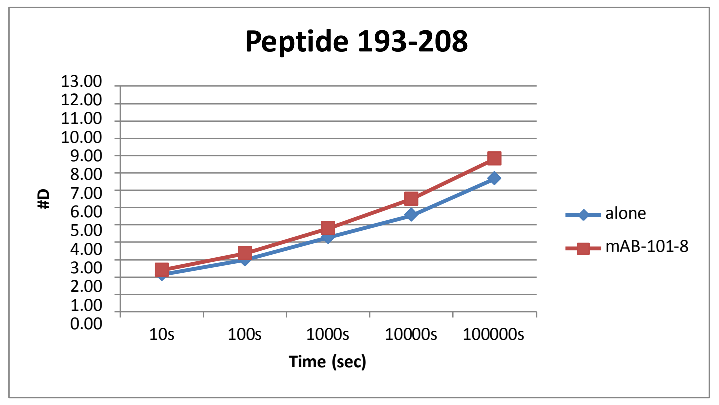

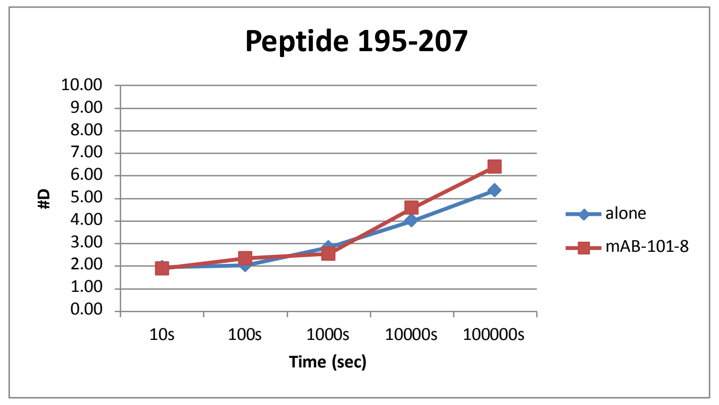

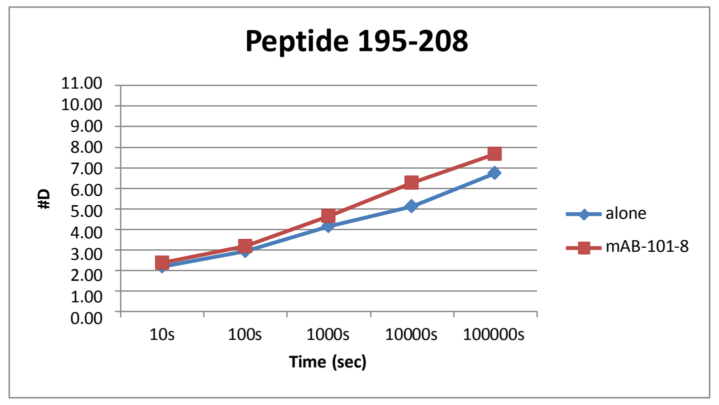

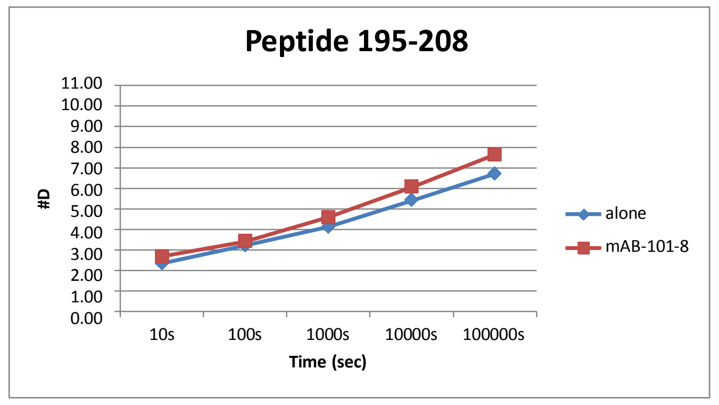

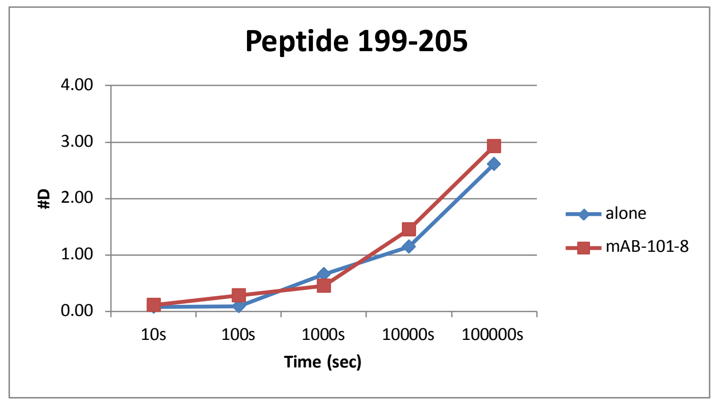

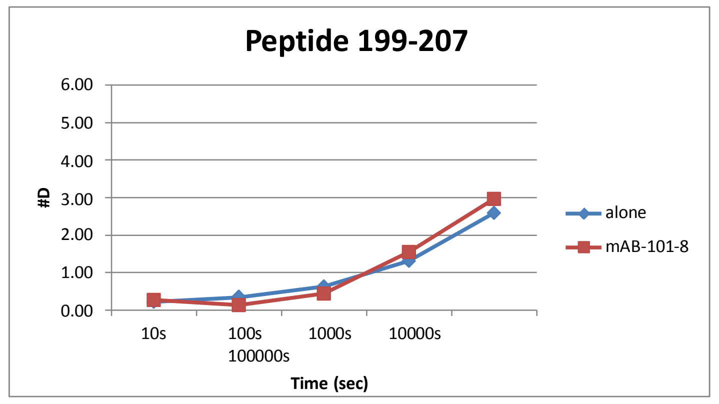

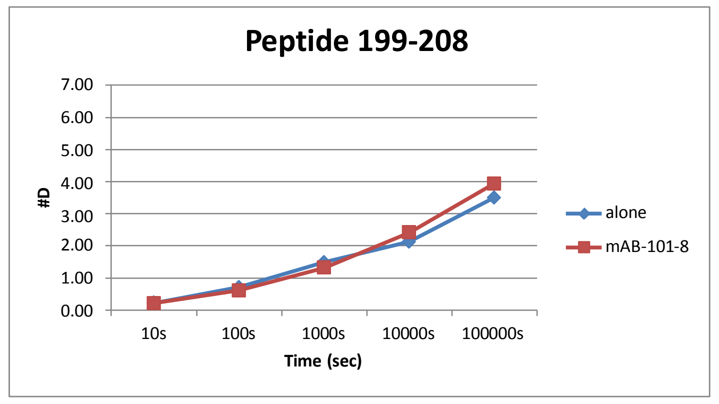

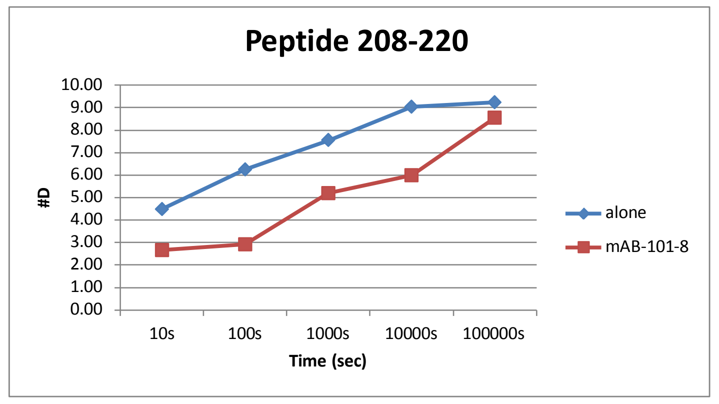

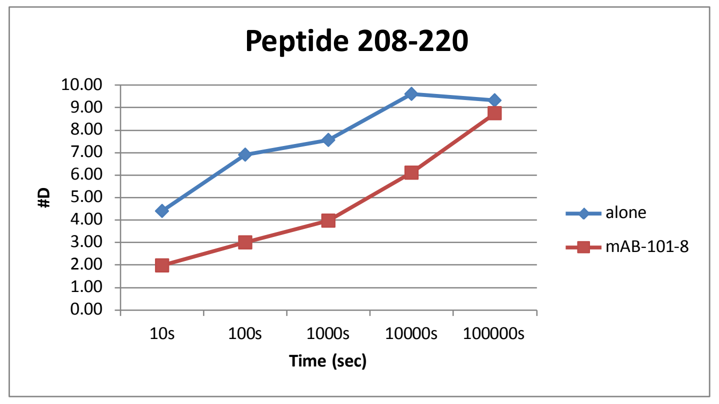

SUPPLEMENTARY FIGURE 2 (CONTINUED)

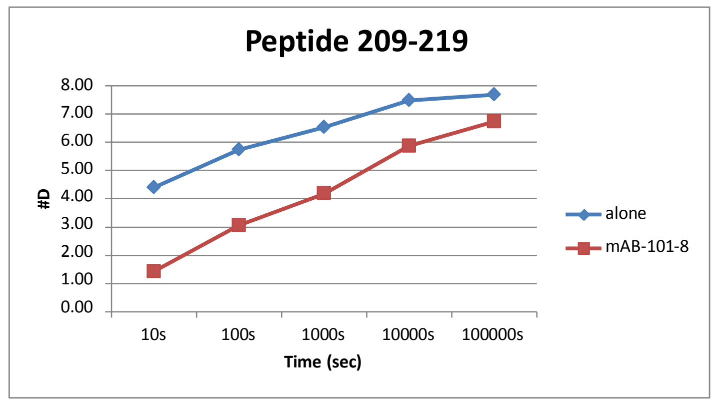

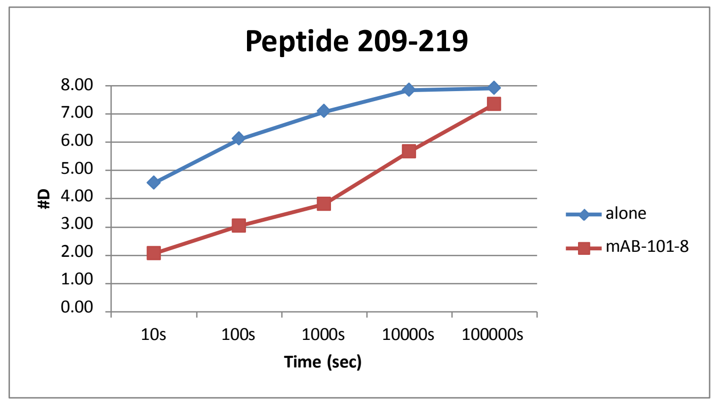

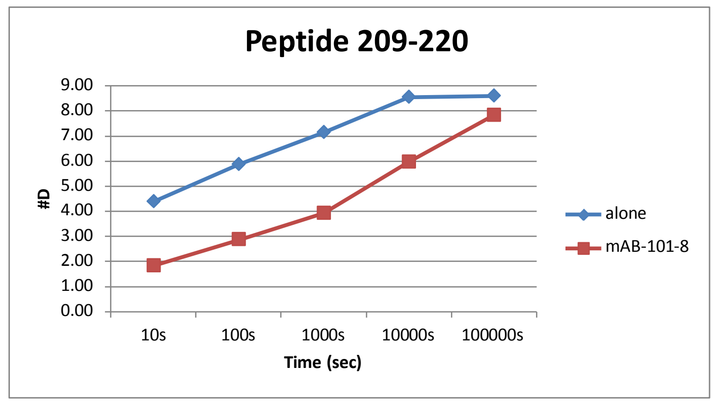

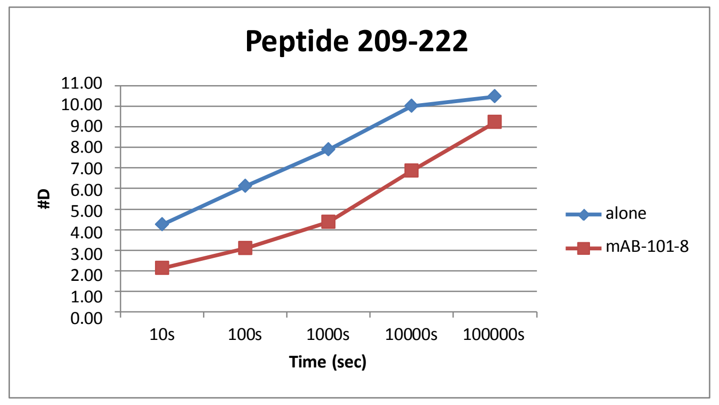

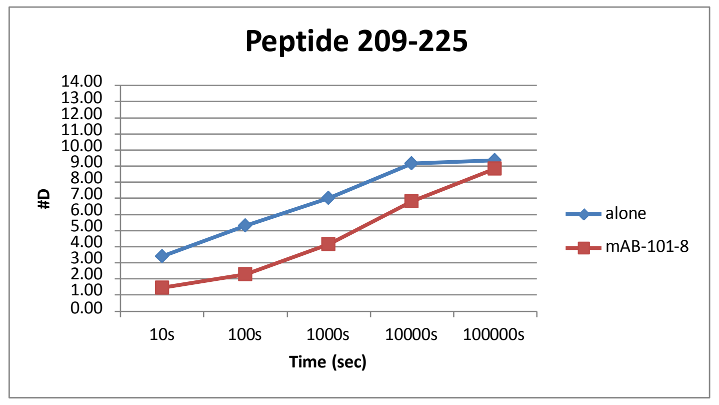

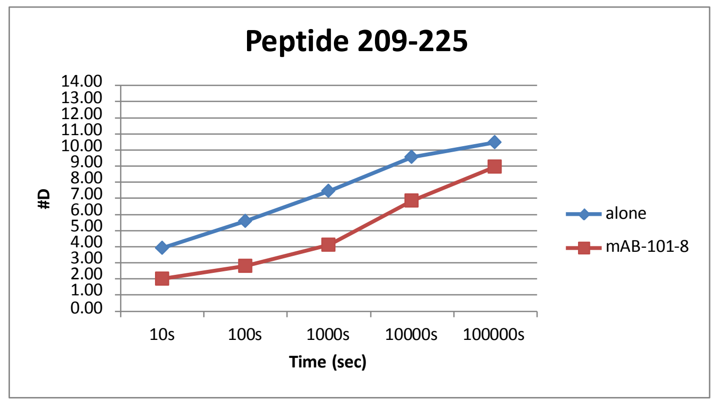

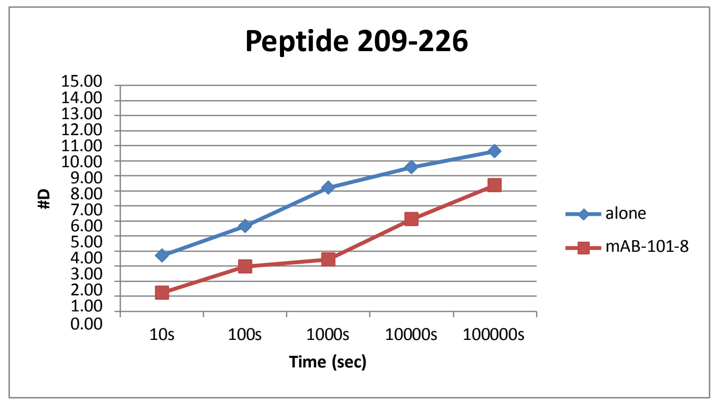

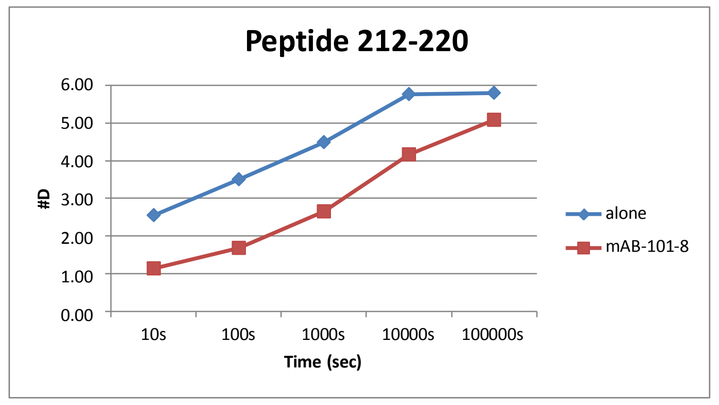

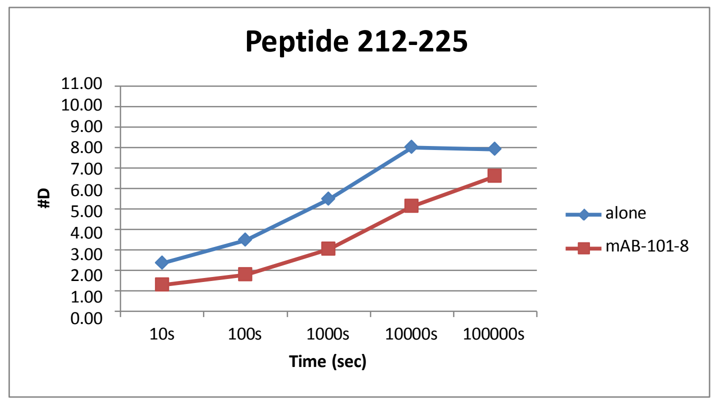

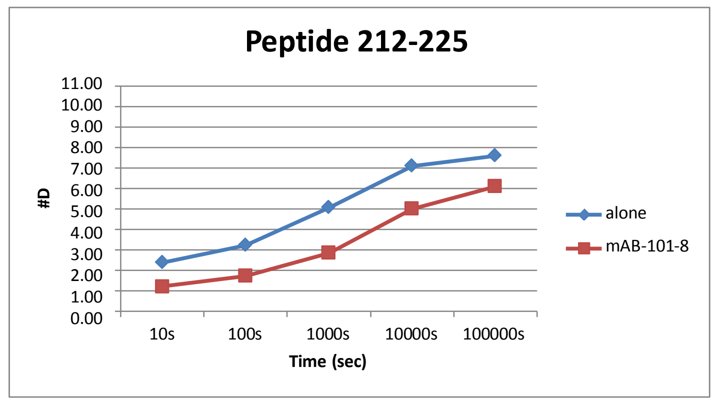

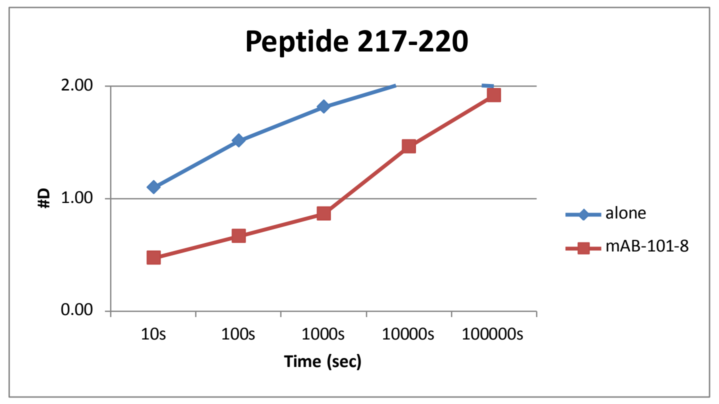

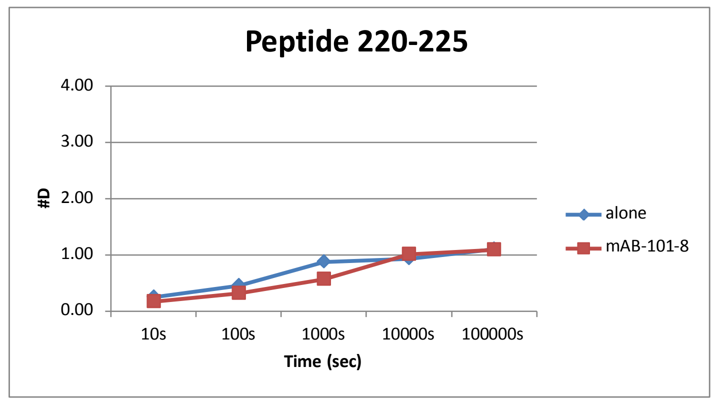

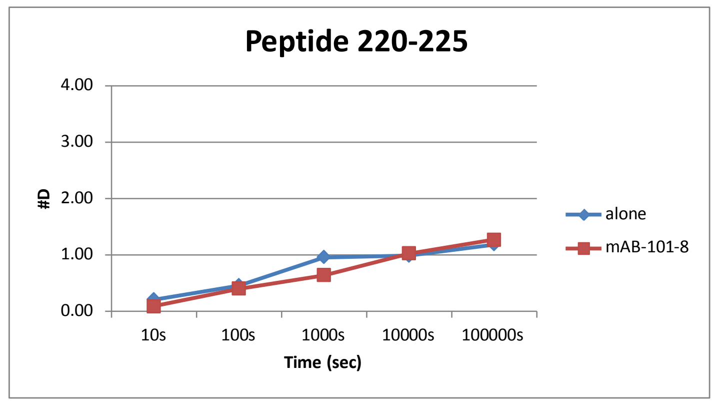

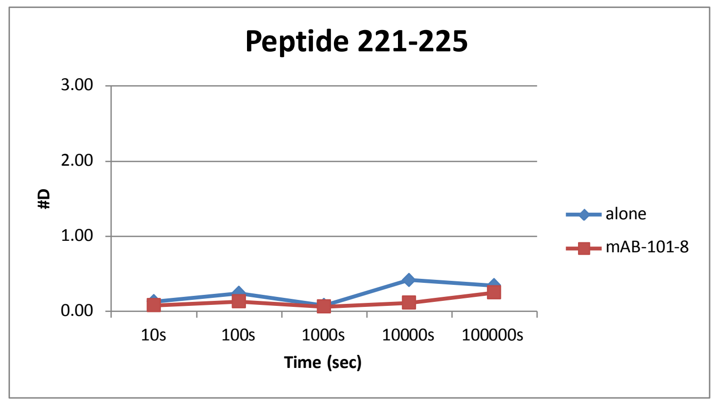

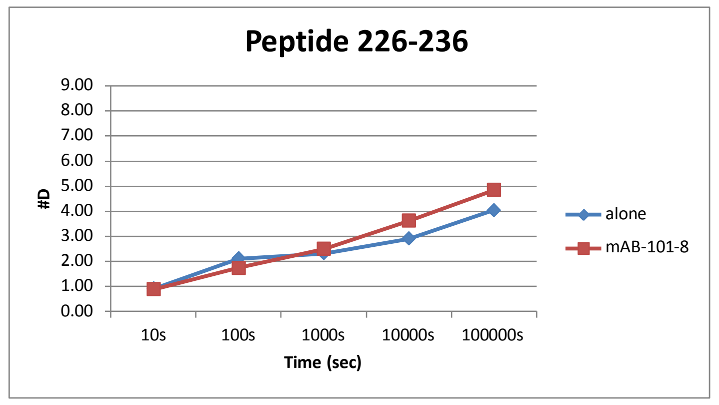

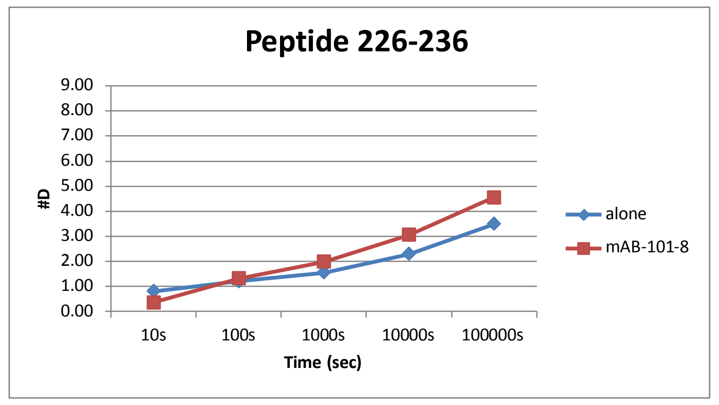

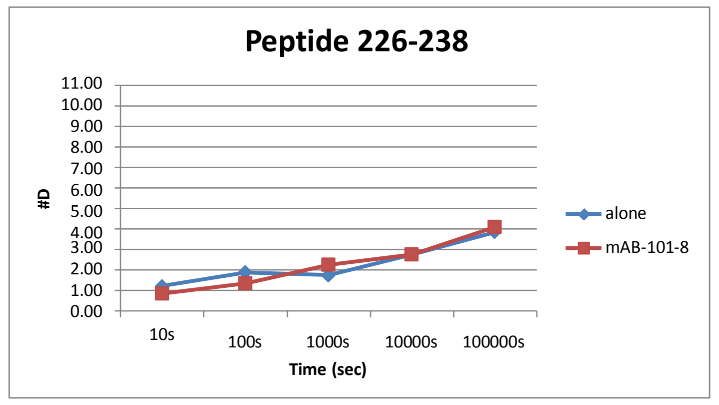

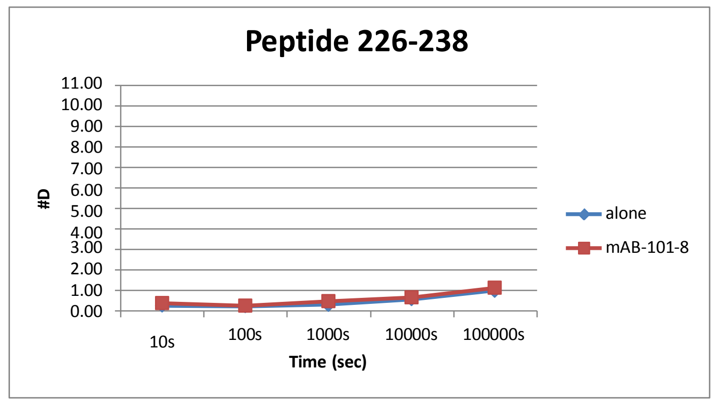

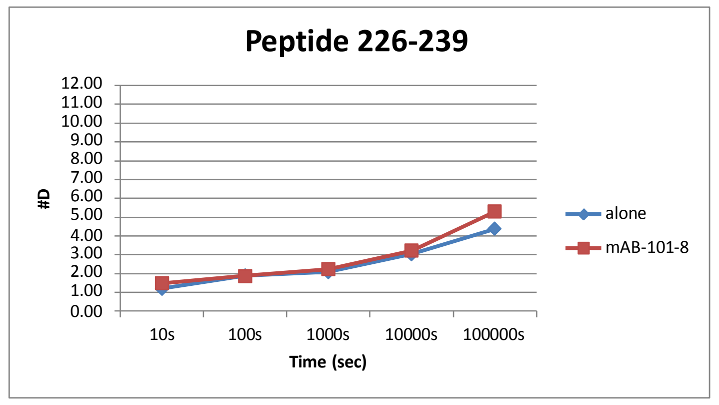

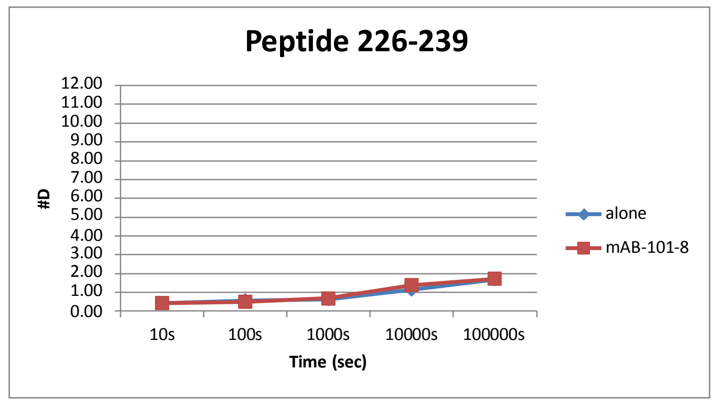

SUPPLEMENTARY FIGURE 2 (CONTINUED)

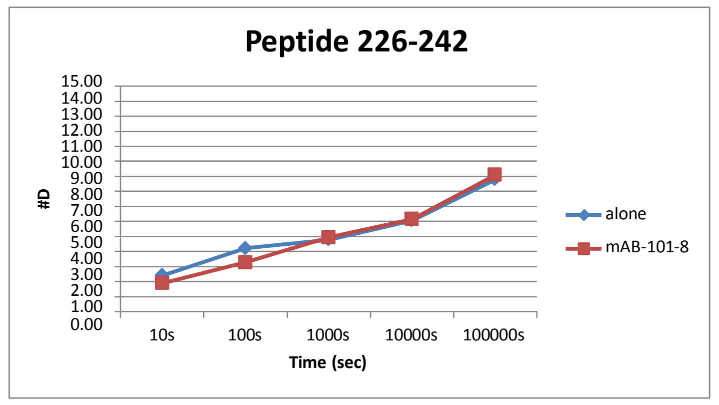

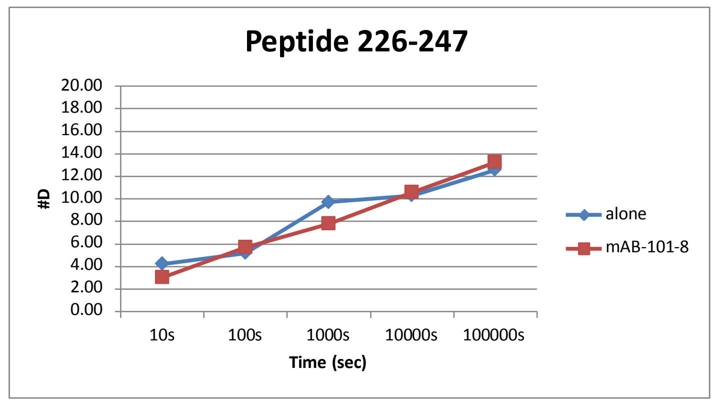

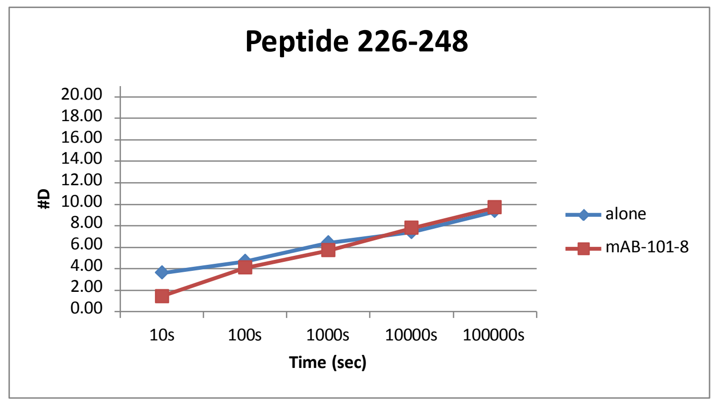

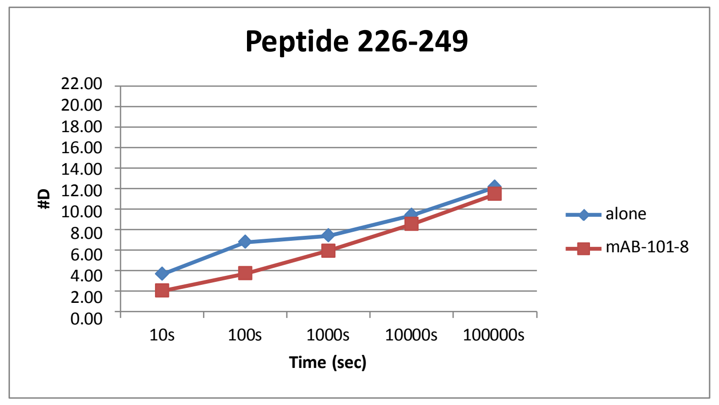

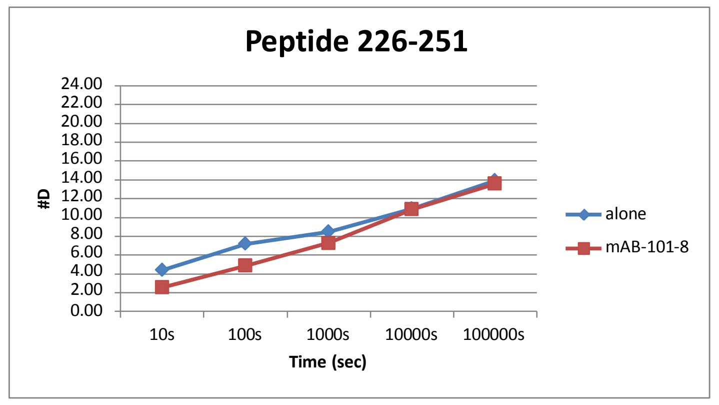

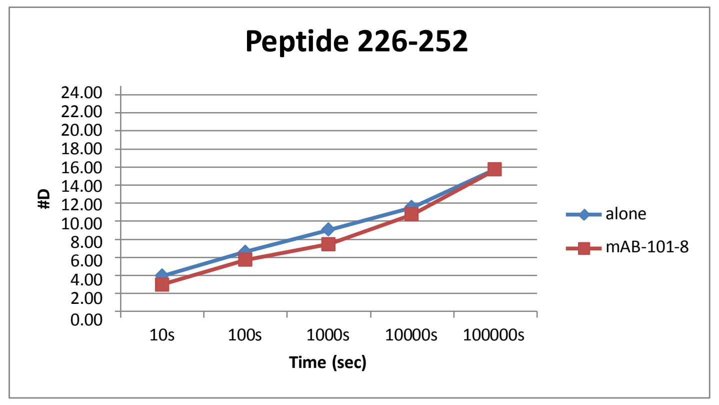

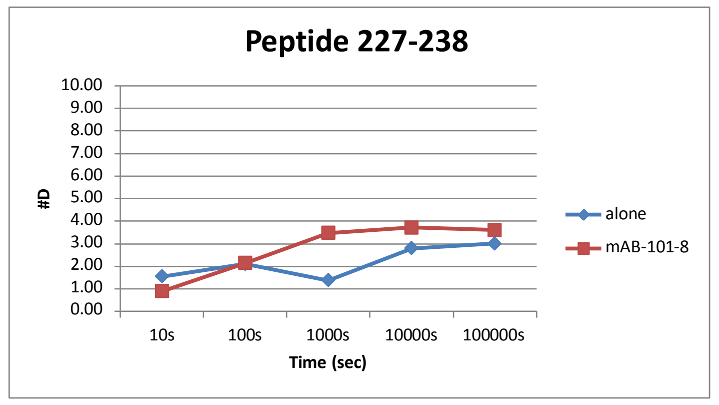

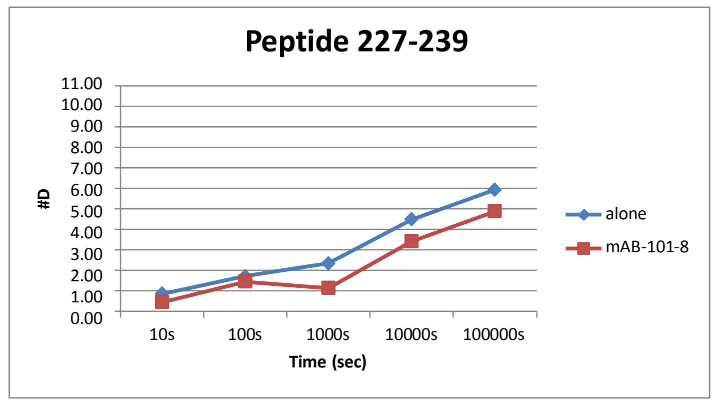

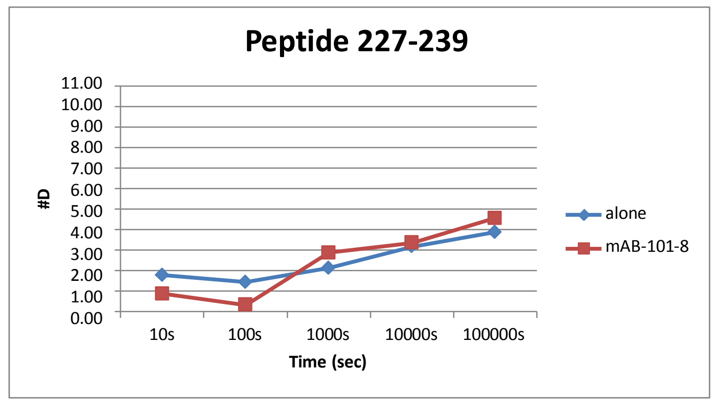

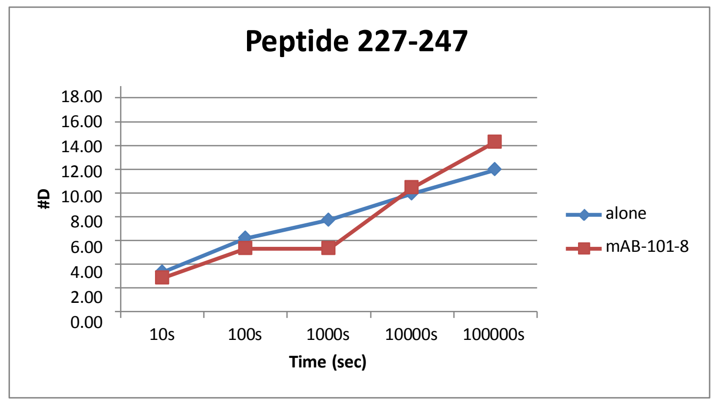

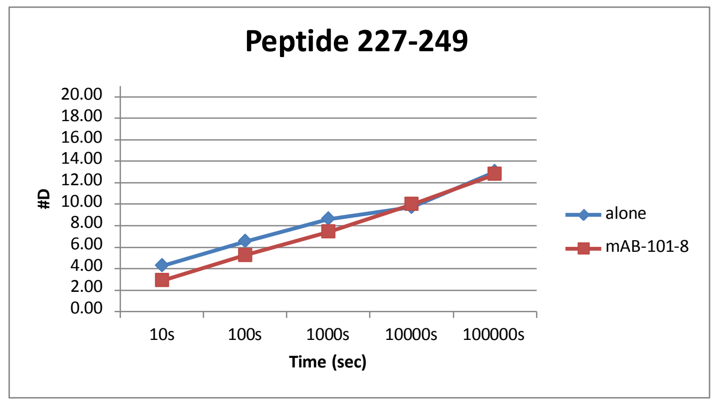

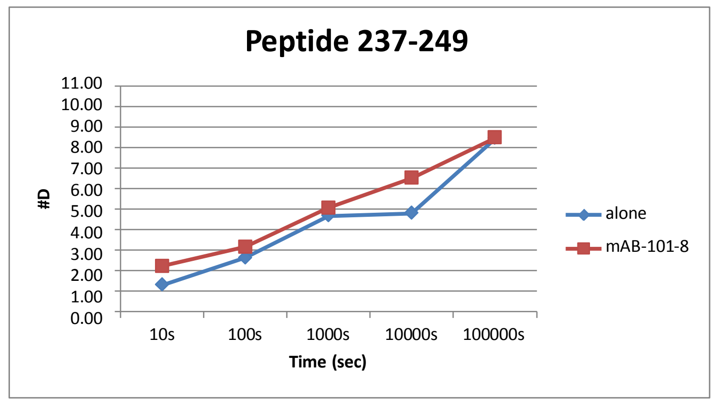

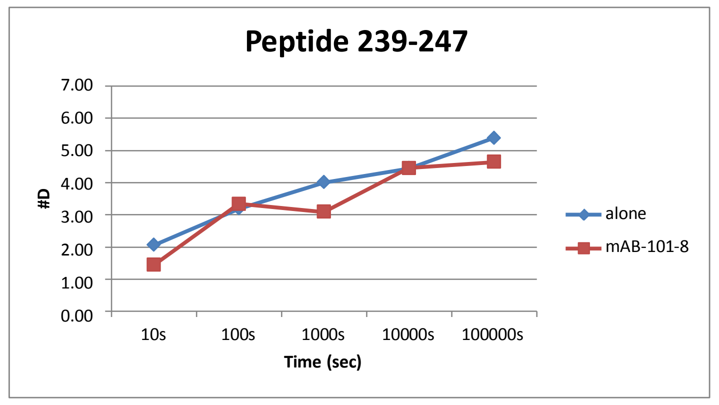

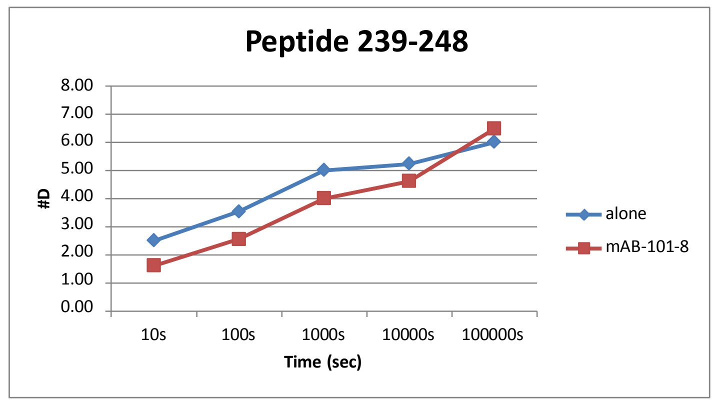

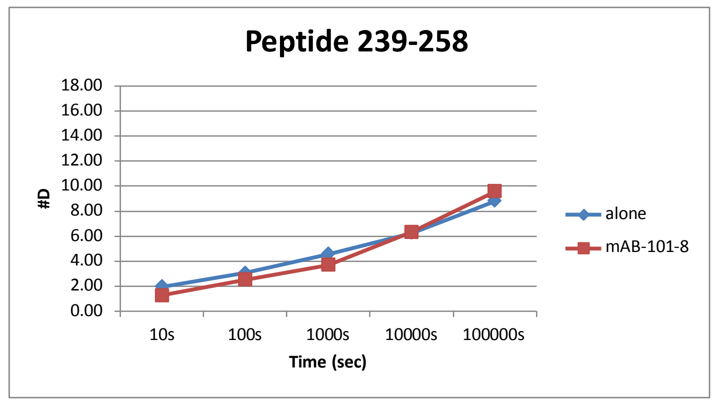

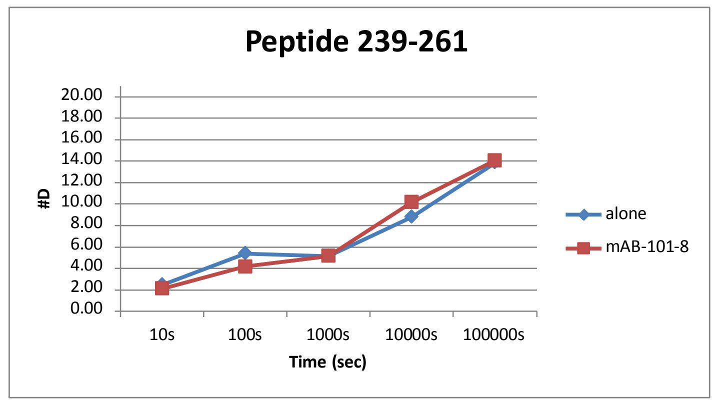

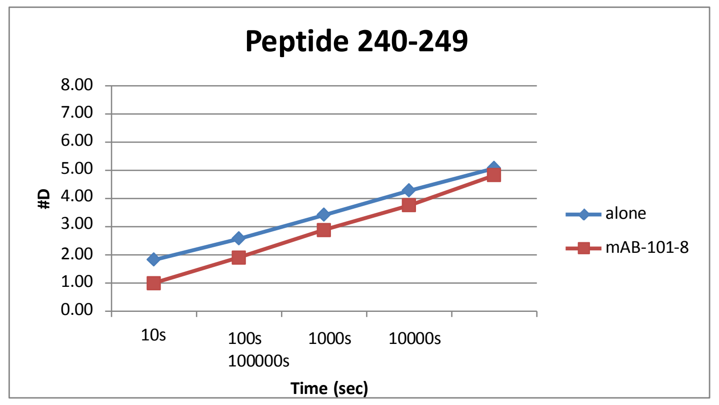

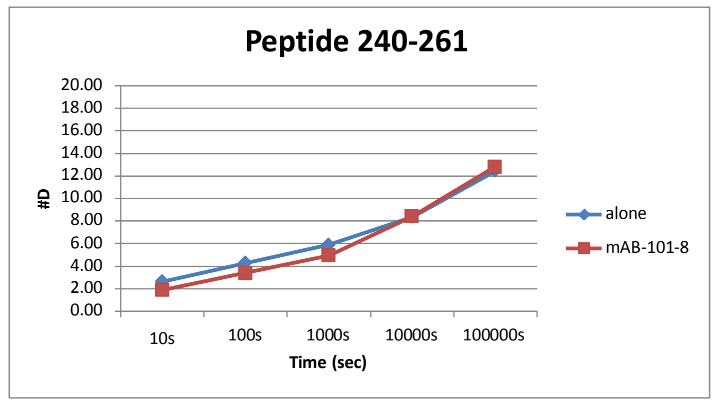

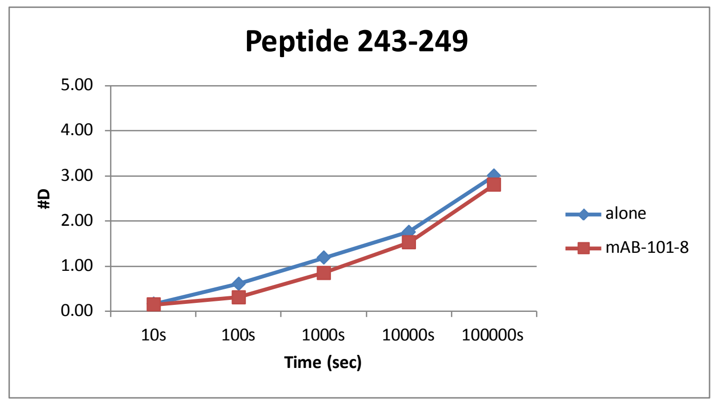

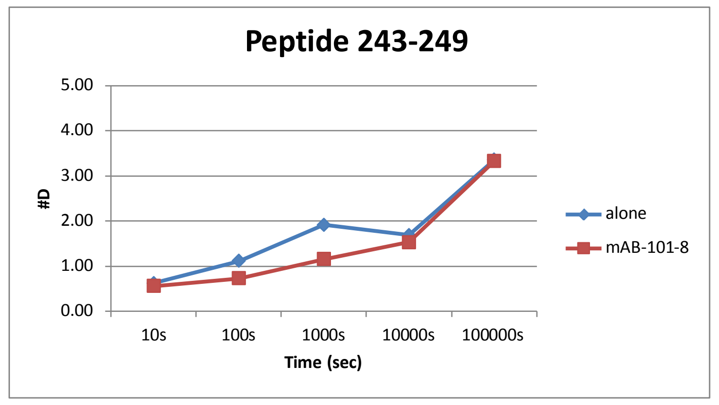

SUPPLEMENTARY FIGURE 2 (CONTINUED)

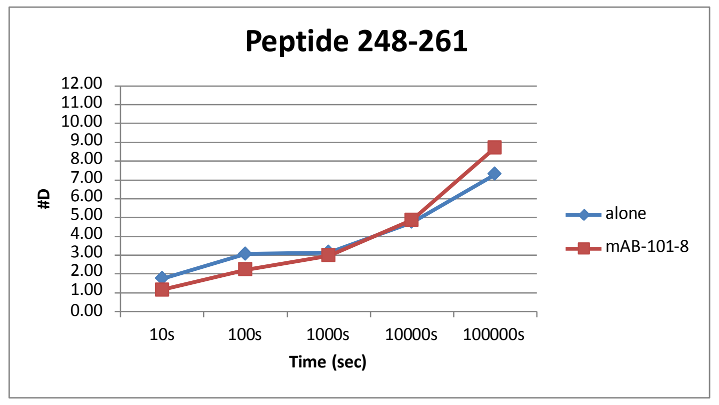

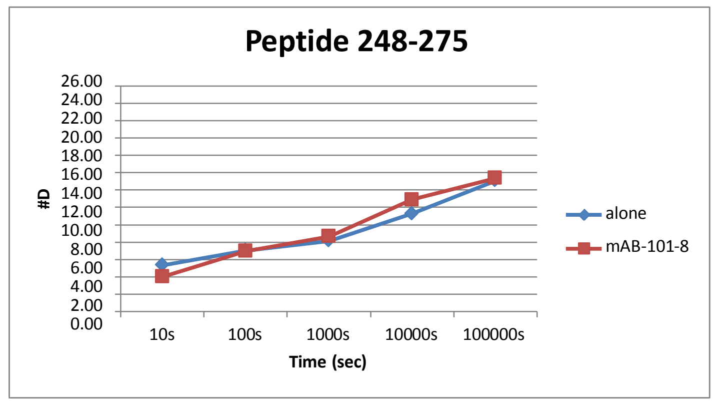

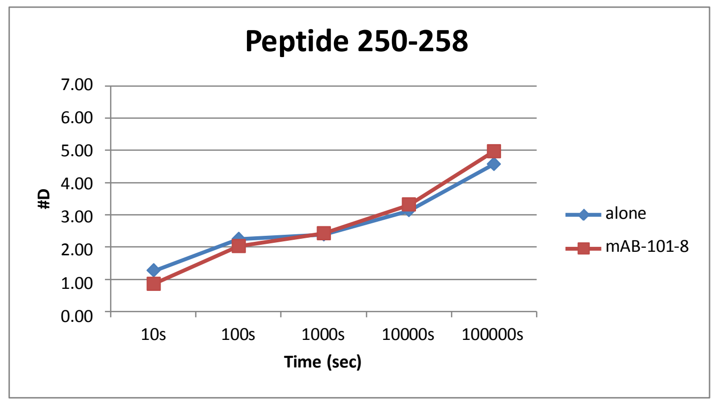

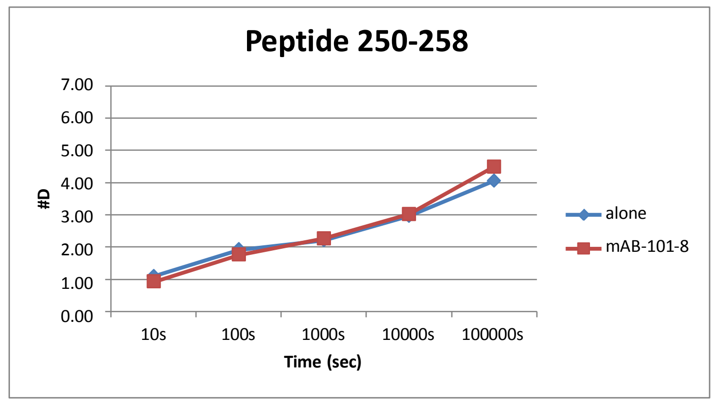

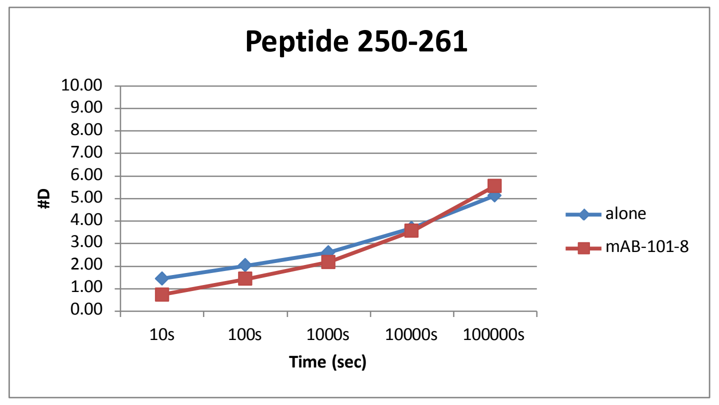

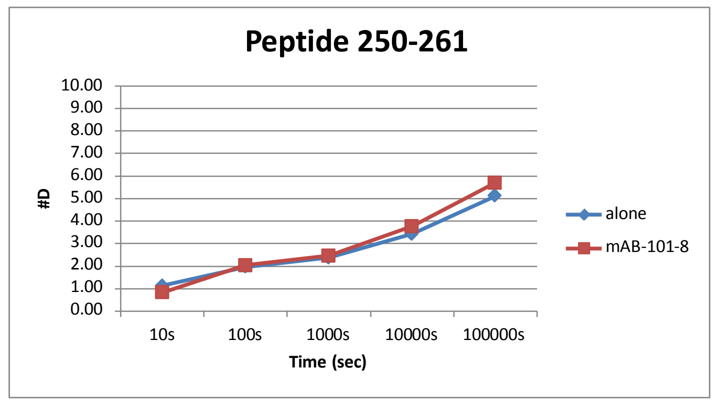

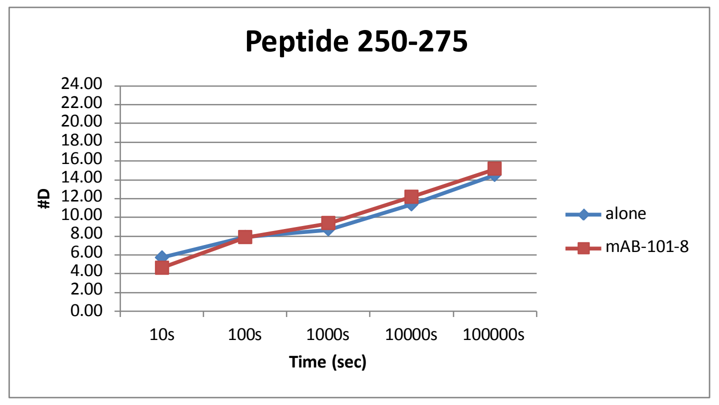

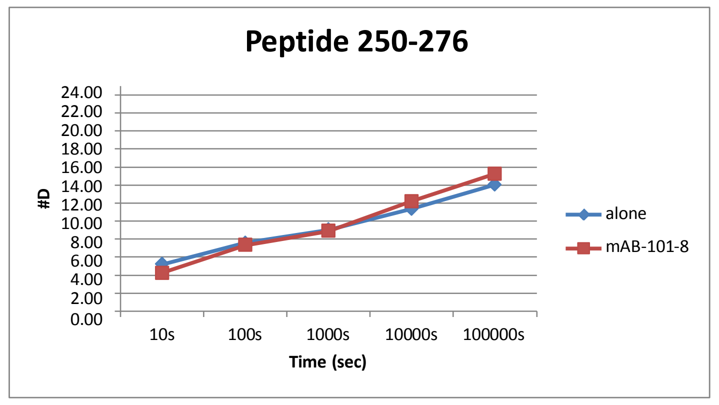

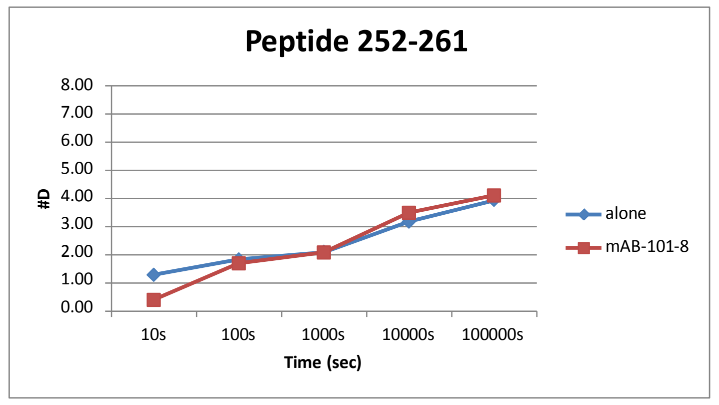

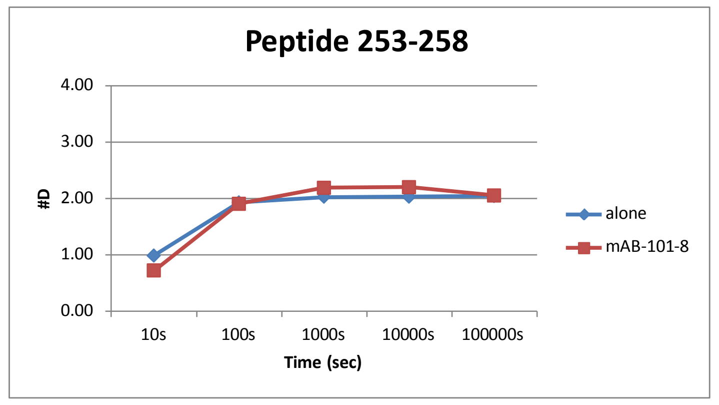

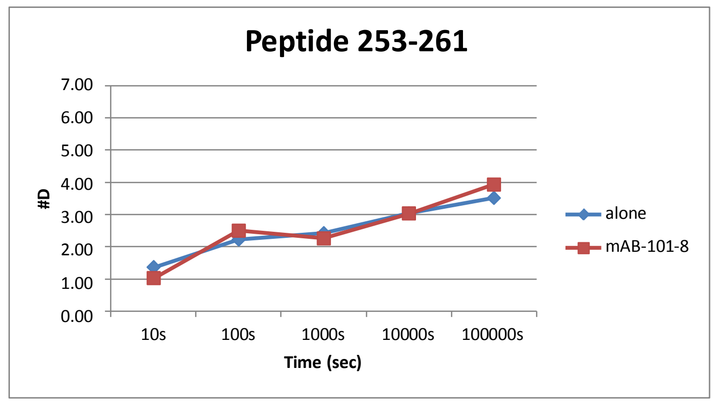

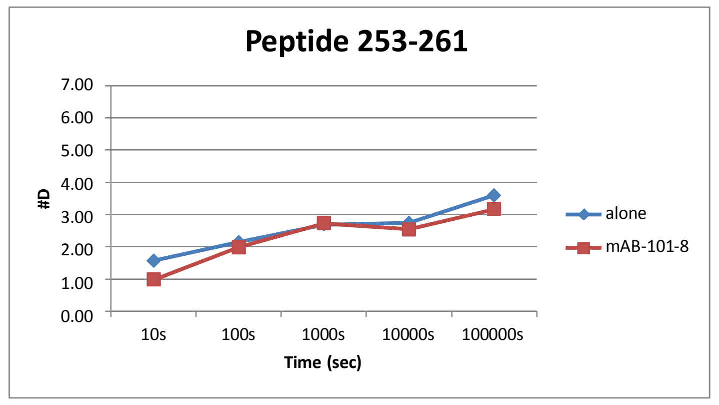

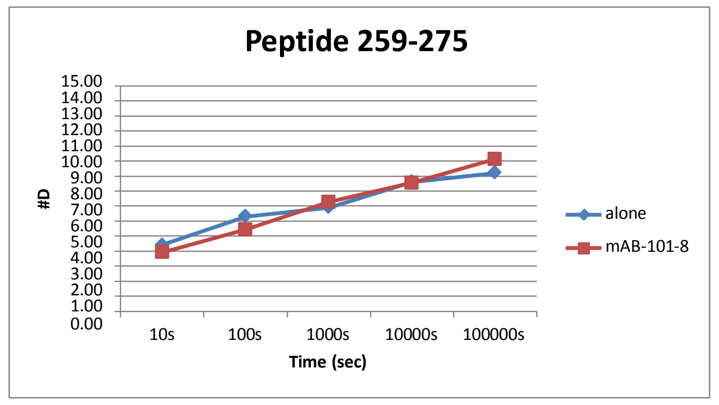

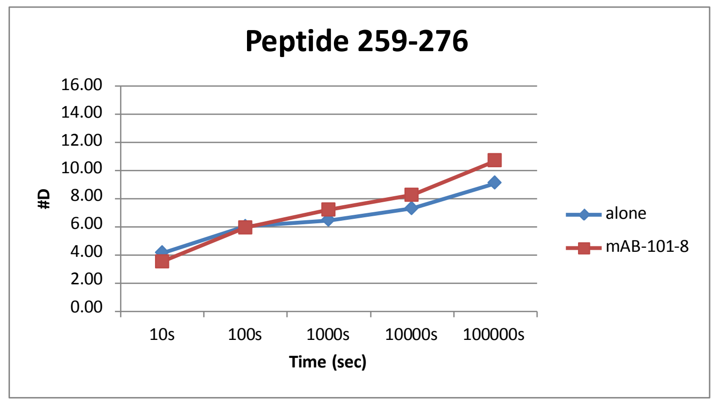

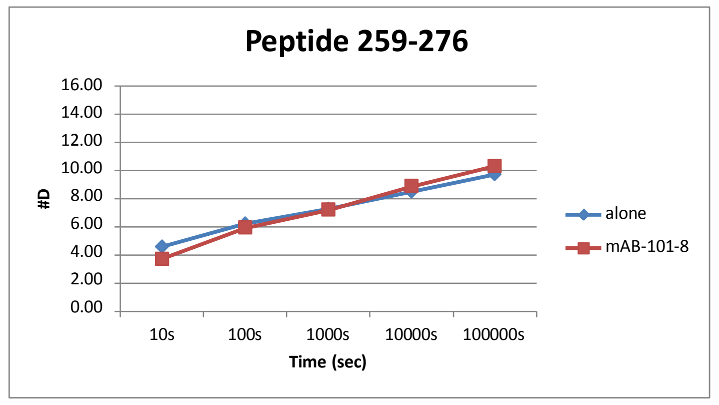

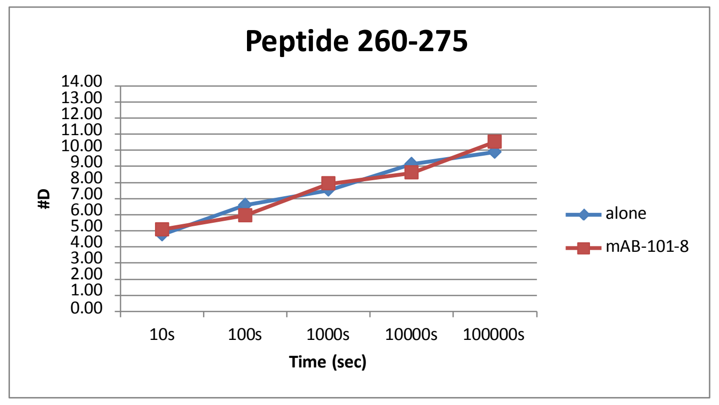

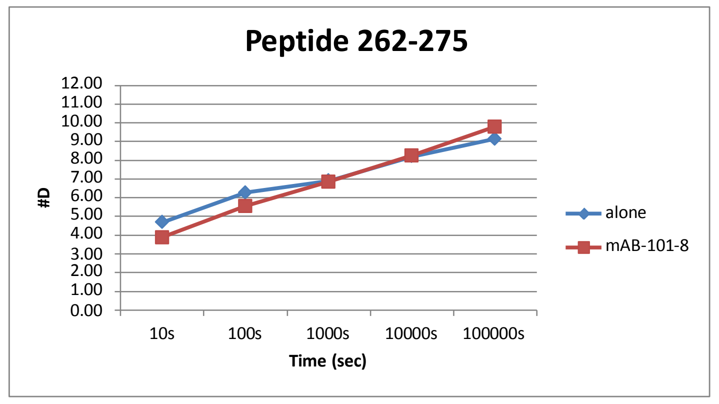

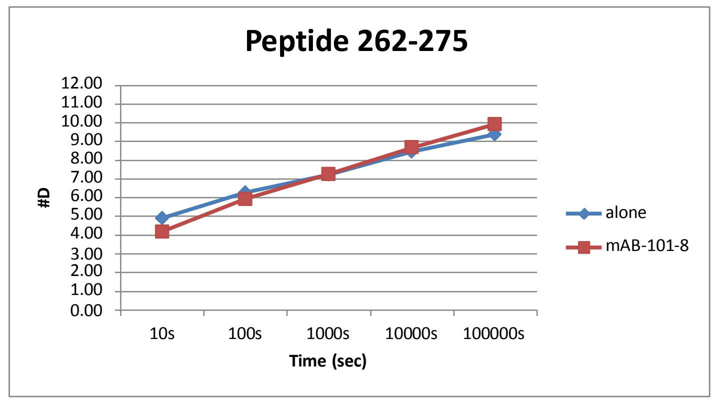

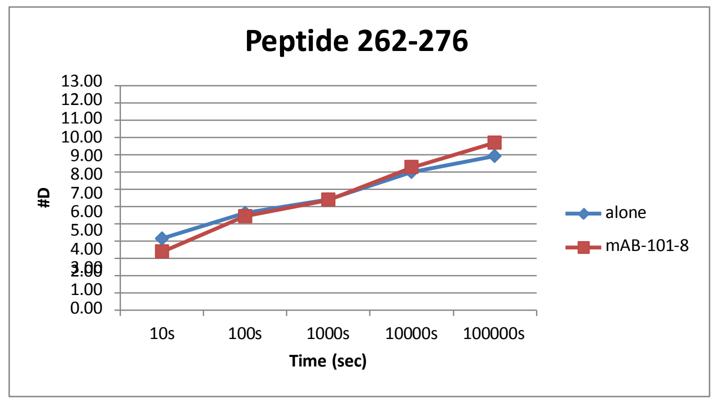

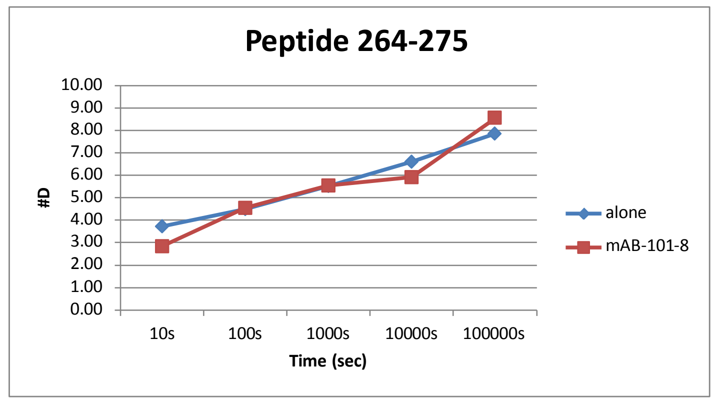

SUPPLEMENTARY FIGURE 2 (CONTINUED)

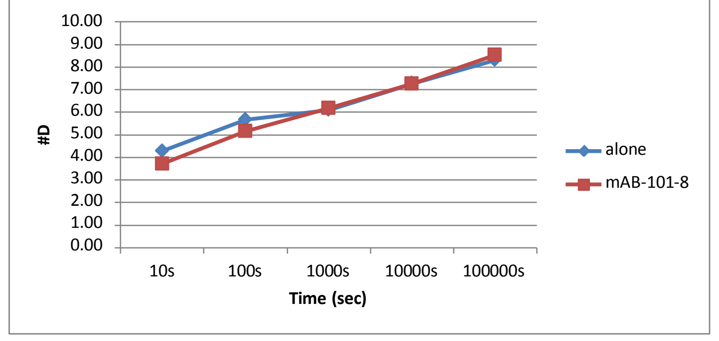

Peptide 264-275

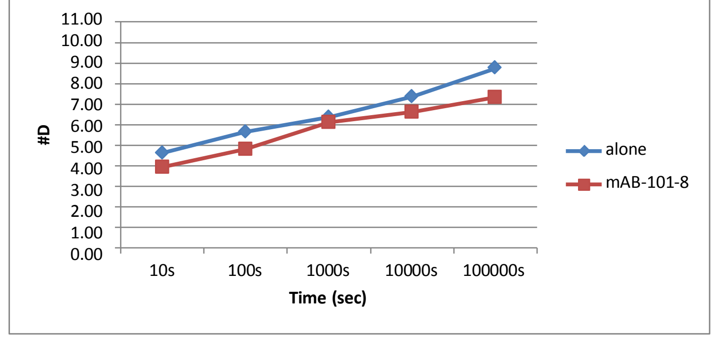

Peptide 264-276

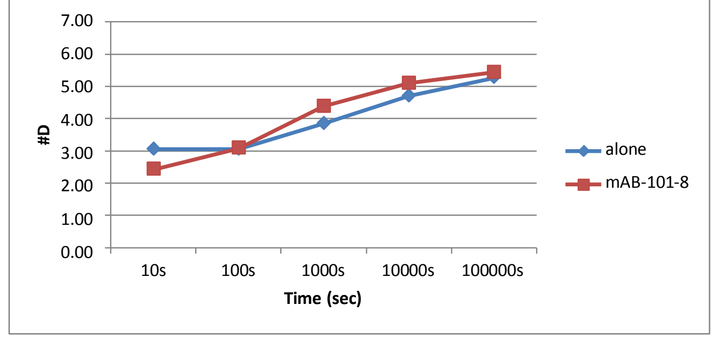

Peptide 267-275

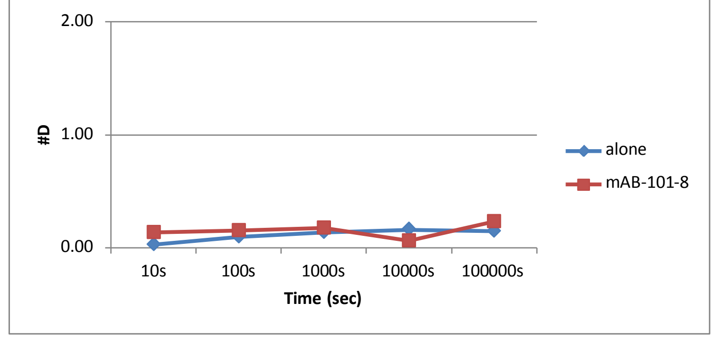

Peptide 276-279

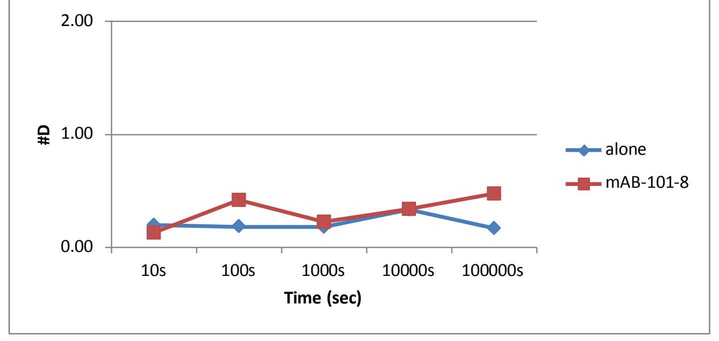

Peptide 276-279

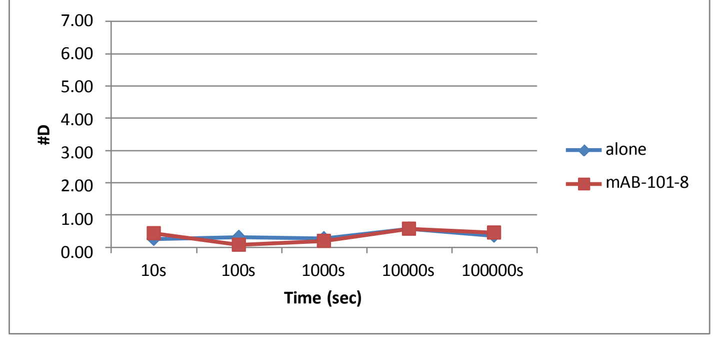

Peptide 276-284

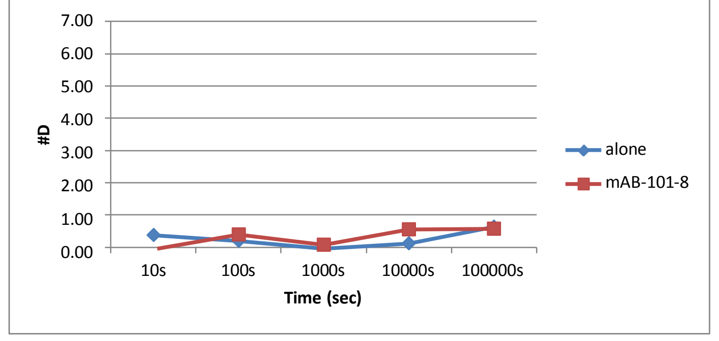

Peptide 276-284

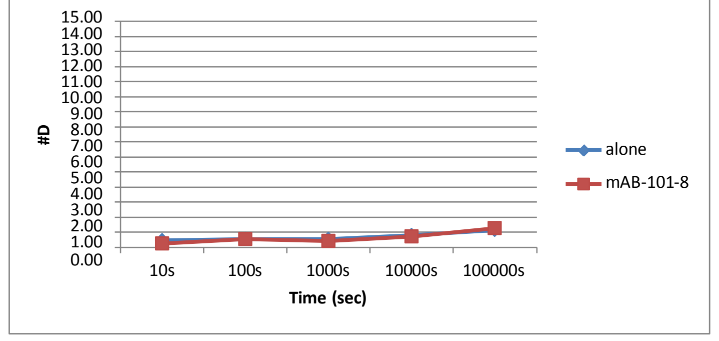

Peptide 276-292

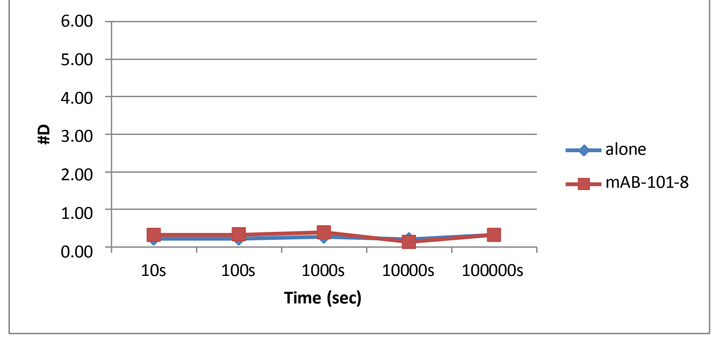

Peptide 277-284

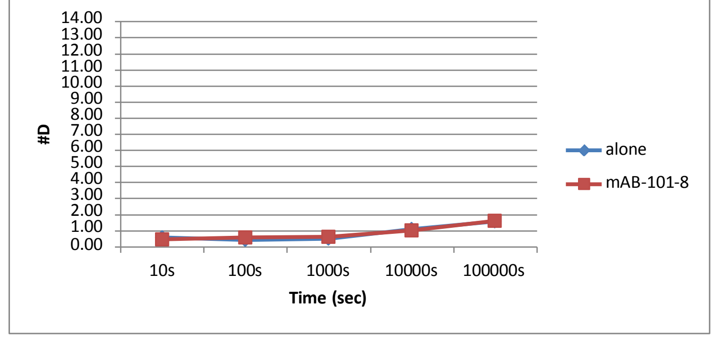

Peptide 277-292

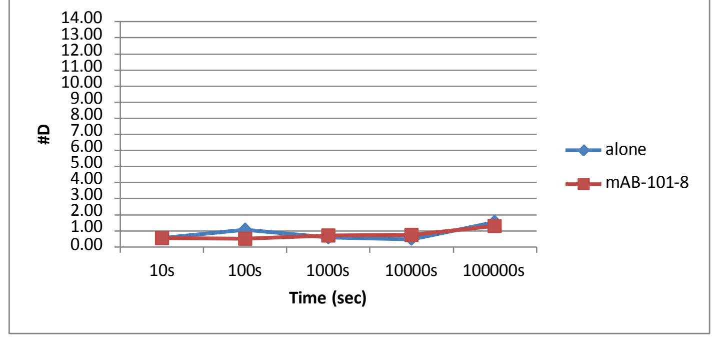

Peptide 277-292

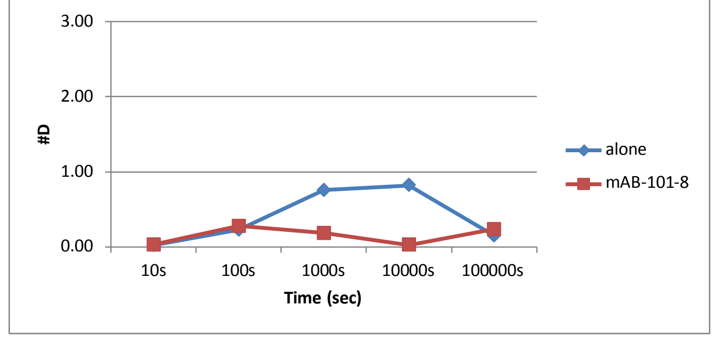

Peptide 280-284

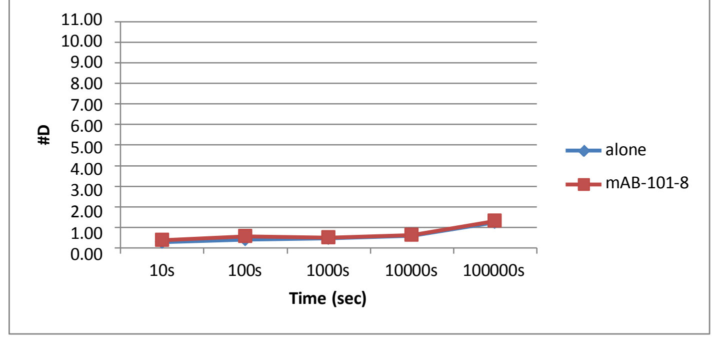

Peptide 280-292

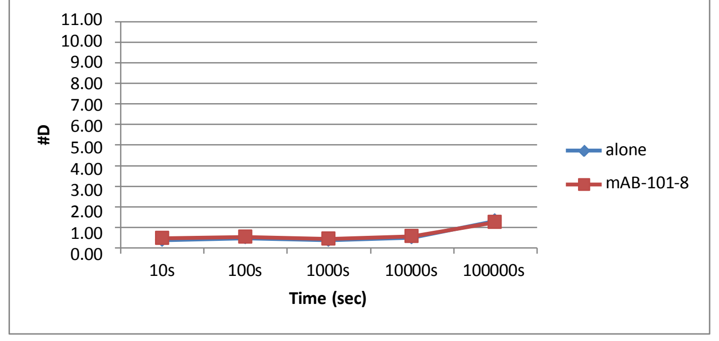

Peptide 280-292

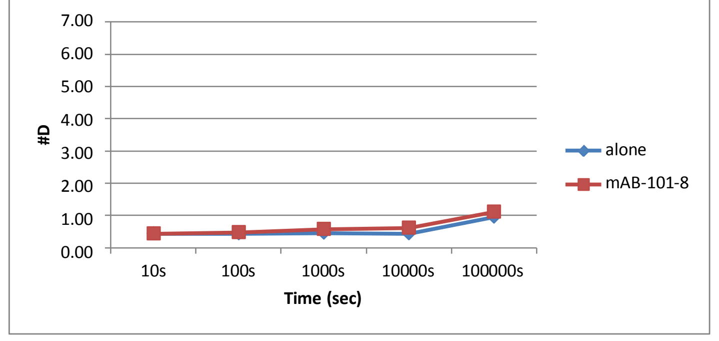

Peptide 284-292

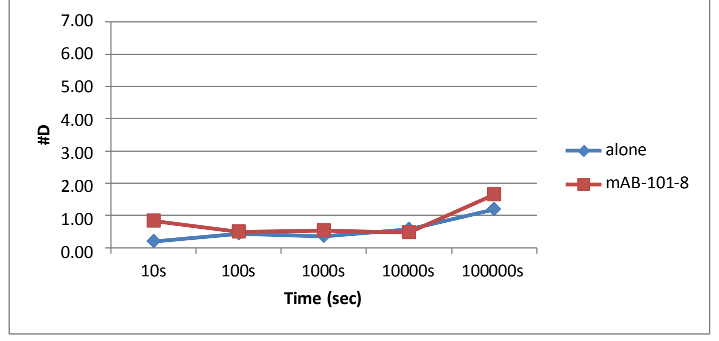

Peptide 284-292

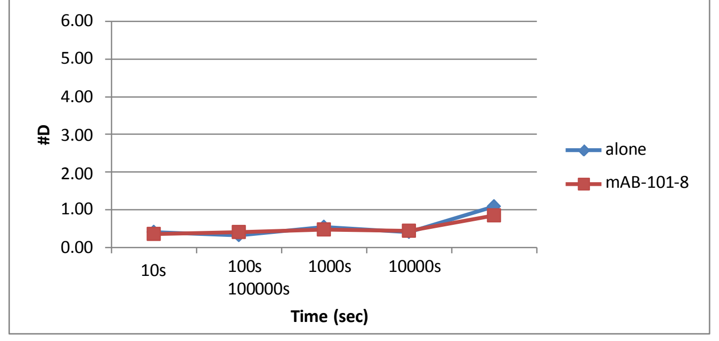

Peptide 285-292

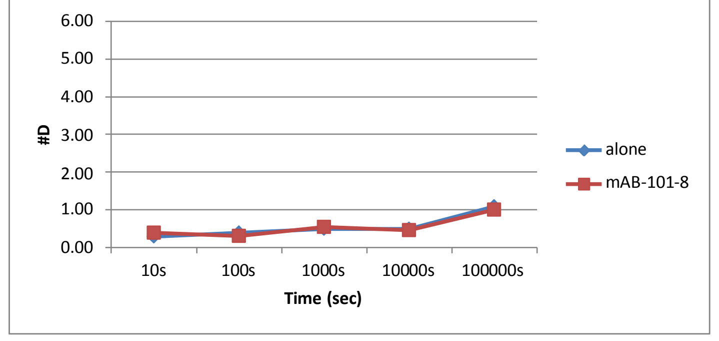

Peptide 285-292
