# Supplementary figures and images for: High Resolution Mapping of Bactericidal Monoclonal Antibody Binding Epitopes on Staphylococcus aureus Antigen MntC
Source: PLoS Pathog. 2016 Sep 30;12(9):e1005908. doi: 10.1371/journal.ppat.1005908 (PMC5045189; doi:10.1371/journal.ppat.1005908)

## SUPPLEMENTARY FIGURE 4

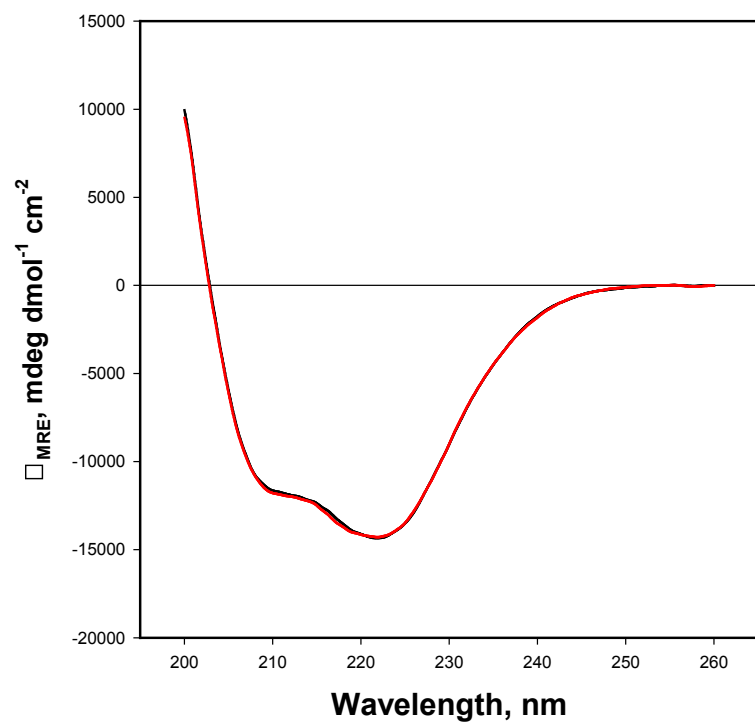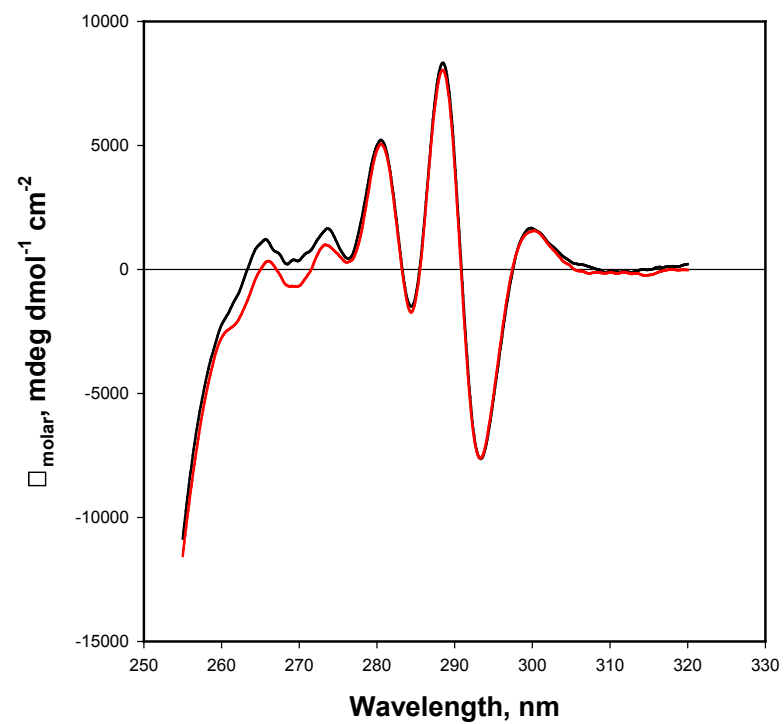

Supplement: S4 Fig — Far-UV (panel A) and near-UV (panel B) CD spectra. Black—spectra of the wild type protein, red—spectra of MntC-pLH94. (PDF) [file ppat.1005908.s004.pdf]

## SUPPLEMENTARY FIGURE 5

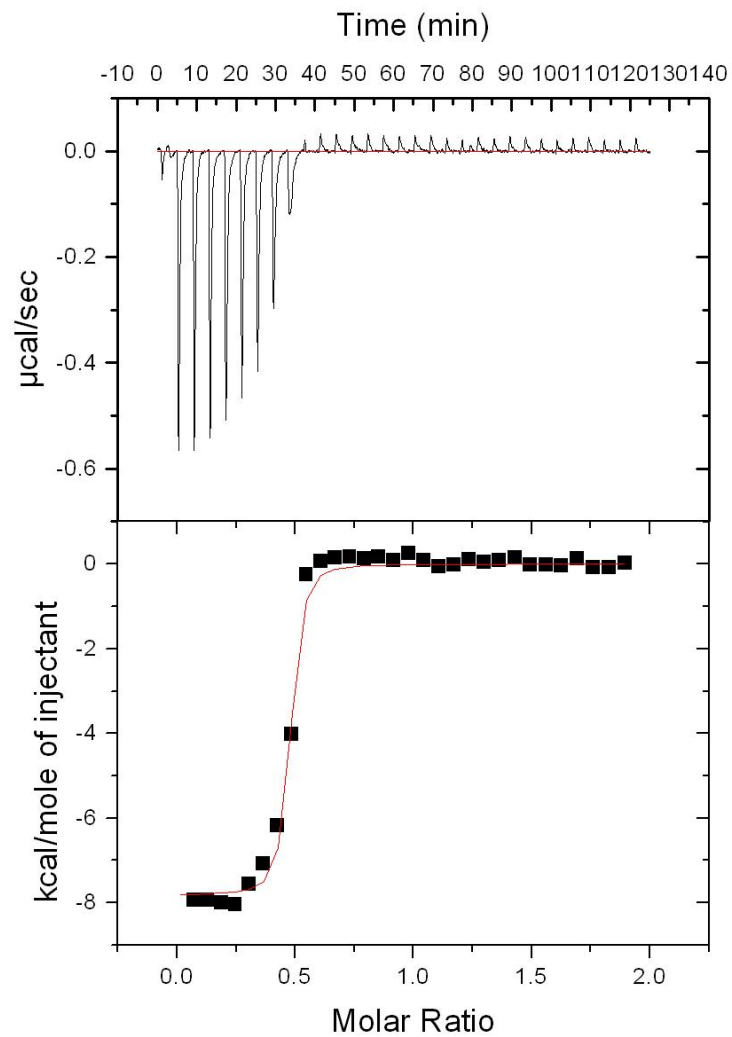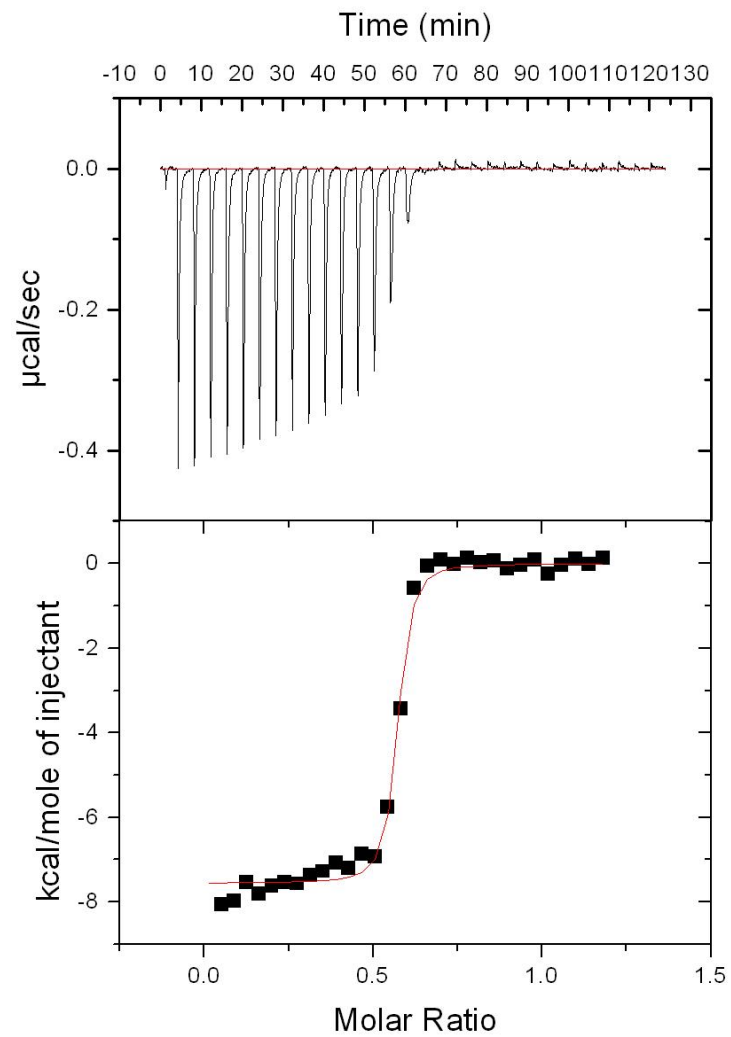

Supplement: S5 Fig — Mn2+ binding studies conducted with wild type MntC (Panel A) and MntC-pLH94 (Panel B) are illustrated. Upper panels show experimental heat flow and lower panels show the integrated heat of each individual injection (symbols). Solid lines in the lower panels show fits of the experimental data to the “single class of binding sites” model. (PDF) [file ppat.1005908.s005.pdf]

# SUPPORTING FIGURE 6

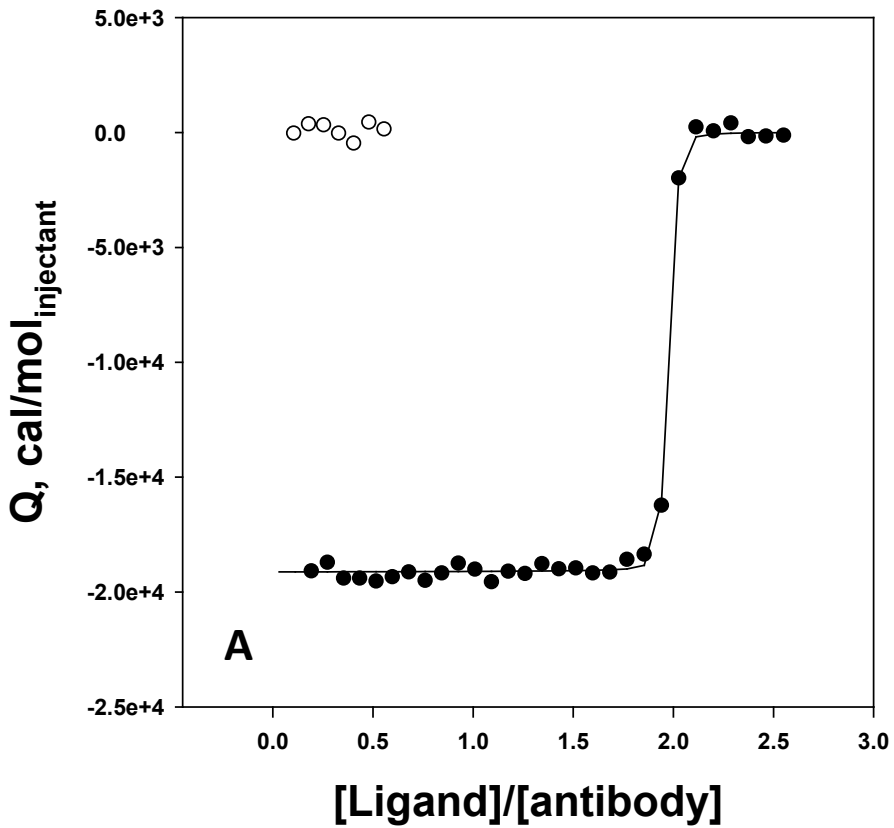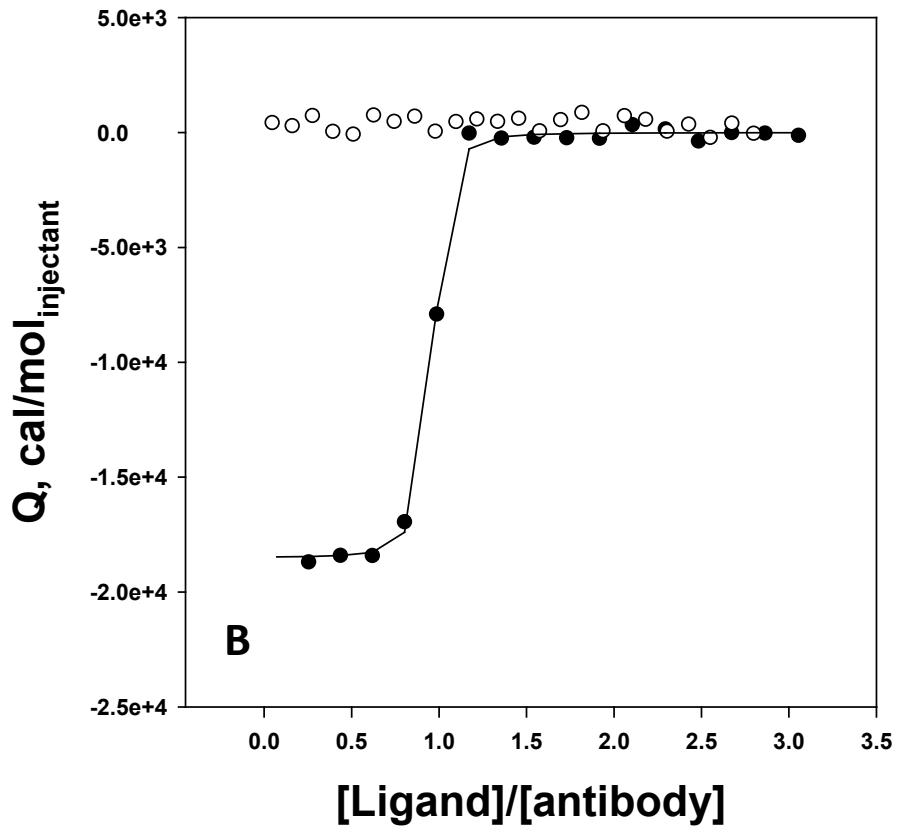

Supplement: S6 Fig — Panel A–experiments with mAB 305-78-7, panel B–experiments with mAB 305-101-8. Open circles: integrated heats of the corresponding peptide injections into the ITC cell containing an appropriate antibody, filled circles—integrated heats of the full length MntC injections into the ITC cell containing either mAB 305-78-7 or mAB 305-101-8. Solid lines—fits to the “single class of binding sites” model. mAB 305-78-7 titration with the synthetic peptide was aborted after 7 injections when it became evident that no heat exchange and, therefore, no interaction is taking place. (PDF) [file ppat.1005908.s006.pdf]
