# Supplementary material for: High Resolution Mapping of Bactericidal Monoclonal Antibody Binding Epitopes on Staphylococcus aureus Antigen MntC
Source: PLoS Pathog. 2016 Sep 30;12(9):e1005908. doi: 10.1371/journal.ppat.1005908 (PMC5045189; doi:10.1371/journal.ppat.1005908)
Supplement: S1 Table — (PDF) [file ppat.1005908.s007.pdf]

SUPPORTING TABLE 1.

Data collection	
Space group	C2
Unit cell dimensions (Å)	a = 179.71; b = 41.73; c =108.40
Resolution range (Å)	48.8 – 1.8
Total number of reflections	291,336
Unique reflections	43,900
Model refinement	
Maximum resolution (Å)	1.8
Number of reflections (free)	43860 (2.236)
R_{work}/R_{free}[a]	20.7/23.6
No. of protein atoms	5392
No. of waters	197
Iron ion	1
Other solvent	1 (glycerol)
Root mean square deviations	
Bonds (Å)	0.010
Angles (°)	1.23

[a] $R_{work} = \Sigma|F_{obs} - |F_{calc}||/\Sigma|F_{obs}|$, where R_{free} is equivalent to R_{work} but calculated for a randomly chosen 4.82% of reflections omitted from the refinement process.
