# Supplementary material for: High Resolution Mapping of Bactericidal Monoclonal Antibody Binding Epitopes on Staphylococcus aureus Antigen MntC
Source: PLoS Pathog. 2016 Sep 30;12(9):e1005908. doi: 10.1371/journal.ppat.1005908 (PMC5045189; doi:10.1371/journal.ppat.1005908)
Supplement: S2 Table — (PDF) [file ppat.1005908.s008.pdf]

Supporting Table 2. Sequences of oligonucleotides used in the study.

oLH553	CAA GCT ATT GAG TTT GTT AAA AAG CAC AAA TTA AAA TTC TTA TTA GTA GAA ACA AGT GTT GAT AA
oLH556	GAA ACA AGT GTT GAT AAG AAA GCA ATG TTA AGT TTA TCT GAA GAA ACG AAG AAA GA
oLH557	GGA AAG TTT ATC TGA AGA AAC GAT GAA AGA TAT CTT TGG TGA AGT GT
oLH569	GAA ACA AGT GTT GAT AAG AAA GCA ATG TTA AGT TTA TCT GAA GAA ACG ATG AAA GA
oLH570	TCT TTC ATC GTT TCT TCA GAT AAA CTT AAC ATT GCT TTC TTA TCA ACA CTT GTT TC
pLP1215 NdeI_S	GCA TAT GGG TAC TGG TGG TAA ACA AAG C
pLP1215 BamHI_AS	CGG ATC CCC AAG GGG TTA TGC TAG TTA TTG C
