# Supplementary material for: High Resolution Mapping of Bactericidal Monoclonal Antibody Binding Epitopes on Staphylococcus aureus Antigen MntC
Source: PLoS Pathog. 2016 Sep 30;12(9):e1005908. doi: 10.1371/journal.ppat.1005908 (PMC5045189; doi:10.1371/journal.ppat.1005908)
Supplement: S3 Table — N—interaction stoichiometry, Ka—association constant, ΔH—enthalpy change upon binding, ΔS—entropy change upon binding, Kd—dissociation constant. (PDF) [file ppat.1005908.s009.pdf]

Supporting Table 3. Thermodynamic parameters of Mn^{2+} binding to the wild type MntC and MntC-pLH94. N – interaction stoichiometry, K_a – association constant, ΔH – enthalpy change upon binding, ΔS – entropy change upon binding, K_d – dissociation constant

	Wt-MntC	MntC-pLH94
**N**	0.45	0.56
**K_a, M^{-1}**	$(2.4\pm0.8)\times10^{7}$	$(4.5\pm1.1)\times10^{7}$
**ΔH, kcal/mol**	-7.8±0.1	-7.6±0.1
**ΔS,(cal/mol) °C^{-1}**	-23.4	-28.3
**K_d, nM**	42	22
